# Efficacy and safety of the investigational complement C5 inhibitor zilucoplan in patients hospitalized with COVID-19: an open-label randomized controlled trial

**DOI:** 10.1186/s12931-022-02126-2

**Published:** 2022-08-09

**Authors:** Elisabeth De Leeuw, Karel F. A. Van Damme, Jozefien Declercq, Cedric Bosteels, Bastiaan Maes, Simon J. Tavernier, Laurent Detalle, Trevor Smart, Sophie Glatt, Nincy Debeuf, Julie Deckers, Sahine Lameire, Stefaan J. Vandecasteele, Nikolaas De Neve, Ingel K. Demedts, Elke Govaerts, Christiane Knoop, Karolien Vanhove, Michel Moutschen, Wim Terryn, Pieter Depuydt, Eva Van Braeckel, Filomeen Haerynck, Tine C. J. Hendrickx, Vanessa Parrein, Marianna Lalla, Claire Brittain, Bart N. Lambrecht

**Affiliations:** 1 Laboratory of Mucosal Immunology, VIB-UGent Center for Inflammation Research, Ghent University, Technologiepark-Zwijnaarde 71, 9052 Ghent, Belgium; 2 Department of Internal Medicine and Pediatrics, Faculty of Medicine and Health Sciences, Ghent University, Ghent, Belgium; 3 Department of Respiratory Medicine, Ghent University Hospital, Ghent, Belgium; 4 Primary Immunodeficiency Research Lab, Faculty of Medicine and Health Sciences, Ghent University, Ghent, Belgium; 5 UCB Biopharma SRL, Braine-l’Alleud, Belgium; 6 UCB Pharma, Slough, UK; 7 Department of Infectious Diseases, AZ Sint-Jan Brugge-Oostende, Brugge, Belgium; 8 Department of Anesthesiology and Intensive Care Medicine, OLV Hospital, Aalst, Belgium; 9 Department of Respiratory Medicine, AZ Delta Roeselare-Menen, Roeselare, Belgium; 10 Department of Pulmonary Medicine, AZ Sint-Lucas Gent, Ghent, Belgium; 11 Department of Pulmonary Medicine, CHU Erasme Université Libre de Bruxelles, Brussels, Belgium; 12 Department of Pneumology and Respiratory Oncology, AZ Vesalius, Tongeren, Belgium; 13 Department of Infectious Diseases and General Internal Medicine, CHU Sart-Tilman, Université de Liège, Liège, Belgium; 14 Department of General Internal Medicine and Nephrology, Jan Yperman Hospital, Ieper, Belgium; 15 Intensive Care Unit, Ghent University Hospital, Ghent, Belgium; 16 Clinical Trial Center, Pharmacy Department, AZ Sint-Lucas Gent, Ghent, Belgium; 17 Sillar Clinical NV, Sint-Martens-Latem, Belgium

**Keywords:** COVID-19, Complement system, Complement 5, Systemic inflammation

## Abstract

**Background:**

The efficacy and safety of complement inhibition in COVID-19 patients is unclear.

**Methods:**

A multicenter randomized controlled, open-label trial. Hospitalized COVID-19 patients with signs of systemic inflammation and hypoxemia (PaO_2_/FiO_2_ below 350 mmHg) were randomized (2:1 ratio) to receive standard of care with or without the C5 inhibitor zilucoplan daily for 14 days, under antibiotic prophylaxis. The primary outcome was improvement in oxygenation at day 6 and 15.

**Results:**

81 patients were randomly assigned to zilucoplan (n = 55) or the control group (n = 26). 78 patients were included in the safety and primary analysis. Most were men (87%) and the median age was 63 years. The mean improvement in PaO_2_/FiO_2_ from baseline to day 6 was 56.4 mmHg in the zilucoplan group and 20.6 mmHg in the control group (mean difference + 35.8; 95% confidence interval (CI) − 9.4 to 80.9; p = 0.12), an effect also observed at day 15. Day 28 mortality was 9% in the zilucoplan and 21% in the control group (odds ratio 0.4; 95% CI 0.1 to 1.5). At long-term follow up, the distance walked in a 6-min test was 539.7 m in zilucoplan and 490.6 m in the control group (p = 0.18). Zilucoplan lowered serum C5b-9 (p < 0.001) and interleukin-8 (p = 0.03) concentration compared with control. No relevant safety differences between the zilucoplan and control group were identified.

**Conclusion:**

Administration of zilucoplan to COVID-19 patients in this proof-of-concept randomized trial was well tolerated under antibiotic prophylaxis. While not reaching statistical significance, indicators of respiratory function (PaO_2_/FiO_2_) and clinical outcome (mortality and 6-min walk test) suggest that C5 inhibition might be beneficial, although this requires further research in larger randomized studies*.*

**Supplementary Information:**

The online version contains supplementary material available at 10.1186/s12931-022-02126-2.

## Background

*What is already known on this topic: *Dysregulated complement activation has been implicated in the pathophysiology of COVID-19. Despite many ongoing trials, only one interventional trial targeting the complement system has been published so far, which was not powered to assess efficacy endpoints (PANAMO trial, NCT04333420).

*What this study adds:* While the improved oxygenation upon C5 blockade did not reach statistical significance, a Bayesian approach suggested that COVID-19 patients with hypoxia and systemic inflammation on zilucoplan had a 89% chance to fare better. No safety signals for C5 inhibition emerged under prophylactic antibiotics.

*How this study might affect research, practice or policy:* This study supports further research on C5 blockade in COVID-19.

## Introduction

Coronavirus disease 2019 (COVID-19) caused by severe acute respiratory syndrome coronavirus 2 (SARS-CoV-2) can progress from an initially mild upper airway disease to acute respiratory failure accompanied by excessive inflammation in the lung alveoli, and by microthrombi in the alveolar capillaries that impede pulmonary gas exchange [1]. Despite vaccination and public health efforts to prevent spread, variants of the virus continue to cause morbidity and mortality and after 2 years of extensive research, there are still few effective treatments that prevent or improve respiratory failure due to COVID-19.

Evidence implicates excessive activation of the complement system in the progression from mild COVID-19 disease to frank respiratory failure with thrombo-inflammation [2–12]. The complement system is part of the ancient innate immune system. Three different pathogen sensing pathways (the classical, alternative and lectin pathway) converge on the activation of C5 convertase, that cleaves C5 in two components. The anaphylatoxin C5a recruits inflammatory cells into tissues [2] and C5b sparks a further complement cascade reaction building up the membrane attack complex (C5b-9) that can kill damaged cells and opsonized pathogens, and also triggers formation of microthrombi on endothelial cells [4, 5, 10, 13–28]. In the clinical setting, several studies showed that increased complement activation is associated with a worse clinical outcome in COVID-19 patients [5, 20–27]. Not surprisingly, complement blockade has emerged as a potential therapy for critically ill COVID-19 patients [11, 29]. Initial case series and non-randomized trials showed promising results of various complement inhibition strategies [30–36], but outcomes of randomized controlled trials have not been reported.

Zilucoplan is an investigational macrocyclic peptide inhibitor of the terminal complement protein C5 that prevents both the formation of active C5a and the membrane attack complex C5b-9 and has been clinically tested in neurological disease [37]. Here, we report the results of a proof-of-concept phase II randomized controlled open-label trial to evaluate the feasibility, efficacy and safety of zilucoplan administration in COVID-19 patients with respiratory failure and signs of systemic inflammation.

## Methods

### Trial design and oversight

We conducted a proof-of-concept phase 2, prospective, randomized, open-label study across 9 hospitals in Belgium. The trial was approved by the Ethical Committee of Ghent University Hospital and conducted in accordance with Good Clinical Practice guidelines and the Declaration of Helsinki. Study design, coordination, monitoring and data management was performed under the responsibility of the Health Innovation and Research Institute UZ Gent (HIRUZ). UCB provided study medication and assistance with data analysis and funded the study. Long-term follow-up of the patients was funded with the ClinicalTrials.COV grant (BOFCOV2020000801) from University Hospital Ghent. An independent data safety monitoring board monitored participant safety. Every patient or their legal representative provided informed consent before participation. All authors take responsibility for the integrity of the trial and the publication.

### Patients

Eligible patients were over the age of 18, had a laboratory diagnosis of COVID-19 with symptoms appearing between 6 and 16 days at inclusion, a ratio of the partial pressure of oxygen (PaO_2_) to the fraction of inspired oxygen (FiO_2_; PaO_2_/FiO_2_) of less than 350 mmHg and bilateral pulmonary infiltrates on chest computed tomography (CT) within the last two days prior to randomization. Presence of systemic inflammation was defined [38] by a single ferritin over 2000 µg/L at inclusion in patients immediately requiring intensive respiratory support. In those without immediate respiratory failure, a ferritin over 1000 µg/L and rising over 24 h needed to be documented, or alternatively lymphopenia below 800/mL with two of the following criteria, any of those rising over 24 h: (1) a rising ferritin above 700 µg/L, (2) a rising lactate dehydrogenase above 300 IU/L, (3) a rising C-reactive protein above 70 mg/L or (4) rising D-dimers above 1000 ng/mL.

Exclusion criteria included mechanical ventilation for more than 24 h at randomization, a clinical frailty score > 3 prior to SARS-CoV-2 infection [39], an unlikelihood to survive beyond 48 h as judged by the treating physician, an active co-infection defined on clinical grounds, thrombocytopenia below 50,000/µL or neutropenia below 1500/µL, active treatment with complement-inhibiting drugs; weight below 54 kg or above 150 kg; high-dose systemic steroid or immunosuppressive drug use for a COVID-19-unrelated disorder. The full list of in- and exclusion criteria can be found in the study protocol (Additional file 1).

### Randomization

Participants were allocated in a 2:1 ratio to the zilucoplan or control arm using simple randomization, stratified by center. Randomization and subsequent data collection were done in an interactive Web Response System (REDCap) [40].

### Procedures

Patients allocated to the zilucoplan group received daily 32.4 mg zilucoplan subcutaneously for 14 days or until discharge (whichever came first) and 2 g ceftriaxone i.v. for maximum 28 days (i.e. during zilucoplan treatment and an additional 14 days after cessation of zilucoplan) as prophylaxis for meningococcal infections, which occur more frequently in C5 deficient states [41]. On clinical grounds, ceftriaxone could be switched to any other antibiotic covering *Neisseria meningitidis*. Following hospital discharge, antibiotics were switched to 500 mg ciprofloxacin once daily until 14 days after the last zilucoplan administration. To control for the effect of antibiotics, patients in the control group also received daily i.v. 2 g ceftriaxone for 7 days or until discharge (whichever occurred first).

### Outcomes

The primary efficacy endpoint was the change in oxygenation (PaO_2_/FiO_2_, P(A-a) O_2_ gradient and a/A PO_2_) from baseline to day 6 and to day 15 or discharge (whichever occurred first). The methods to derive the parameters for oxygenation are described in the Statistical Analysis Plan (Additional file 2).

Secondary objectives were to study the effects of zilucoplan on clinical outcomes (defined by duration of hospital stay, 6-point ordinal scale (1: death; 2: invasive mechanical ventilation; 3: non-invasive ventilation or high flow oxygen devices; 4: hospitalized and requiring supplemental oxygen; 5: hospitalized and not requiring supplemental oxygen; 6: not hospitalized), time to defervescence, supplemental oxygen use and severity of organ failure assessment (SOFA) score), on progression to mechanical ventilation, ARDS, on duration of ICU stay, on all-cause mortality rates, on the rate of nosocomial infections, and on ferritin and C-reactive protein (CRP) serum levels. A follow-up visit was scheduled between 12- and 22-weeks post-randomization to study long-term clinical evolution. A detailed list of the secondary endpoints is provided in the study protocol (Additional file 1).

Certain predefined endpoints were not analyzed due to insufficient and/or missing data, i.e. duration of mechanical ventilation in ventilated patients, time since randomization until first use of high-flow oxygen devices or mechanical ventilation in non-ventilated patients and duration of ICU stay (Additional file 3).

Key safety endpoints included incidence of adverse events (AE) and serious adverse events (SAE) from first day of study treatment until day 28.

### Biomarker quantification

Serum cytokines (IL-6, IL-8, IL-18, IL-1RA, CXCL-10) were quantified using Mesoscale Discovery. Soluble membrane attack complex (sC5b-9) was quantified in cell-free plasma using the MicroVue Complement sC5b-9 Plus ELISA kit (Quidel).

### Statistical analysis

#### Sample size

The target difference was the change from baseline measured in PaO_2_/FiO_2_ (at day 6 and day 15) between the control and the treated group. Given a a priori calculated sample size of 81 participants, 54 on the zilucoplan arm and 27 on the control arm, there was > 85% power to show a significant difference from standard of care (SoC) at the 2 sided 5% level if the underlying treatment difference was an 80-mmHg difference (25% of 320 mmHg, being the mean at hospital admission prior to this study) in the PaO_2_/FiO_2_. This assumed a standard deviation of 105 mmHg and a dropout rate of less than 10%.

#### Efficacy and safety analyses

All efficacy analyses were carried out on the full analyses data set consisting of all participants who received at least 1 dose of zilucoplan, when randomized to the zilucoplan group, and who had at least one dose of intravenous prophylactic antibiotics, when randomized to the control group. The primary endpoints were analyzed separately using a Mixed Model Repeated Measures (MMRM) analysis with fixed effects for baseline treatment, nominal day (using day 6 and day 15), baseline*nominal day interaction and treatment*nominal day interaction. Participant was fitted as a random effect and an unstructured covariance was used. A last observation carried forward (LOCF) approach was utilized for the participants who were discharged early. If a patient died or withdrew, no data was imputed. The mean change from baseline to day 6 and day 15 for both treatment arms and differences between treatments in mean change from baseline to day 6 and day 15, their 95% CI and p-values were estimated directly from the model. PaO_2_/FiO_2_, and a/A PO_2_ were analyzed on the natural scale. P(A-a)O_2_ gradient required a log transformation therefore the differences from baseline and between treatment are expressed as ratios.

Logistic regression models, including treatment as a factor, were fitted for mortality. The estimated odds ratio, 95% CI and p-value were computed.

The above analyses were augmented with a series of Bayesian analyses which provide a useful, additional interpretation of the results (Additional file 4).

Safety data were analyzed descriptively in all patients who received at least one dose of zilucoplan in the treated group and all patients who had at least one dose of prophylactic antibiotics in the control group (safety population).

Baseline was defined as the last measure prior to dosing, with the exception of clinical laboratory parameters and cytokines. Blood samples were not always prioritized prior to starting dosing hence values up to 2 h post-first dose were included for clinical laboratory parameters and 30 min post-dose for cytokines.

No adjustments were made for multiplicity.

Statistical analysis was performed using SAS version 9·4 and R. The full statistical analysis plan is available as an online supplement (Additional file 2).

## Results

### Patients

**Fig. 1:**
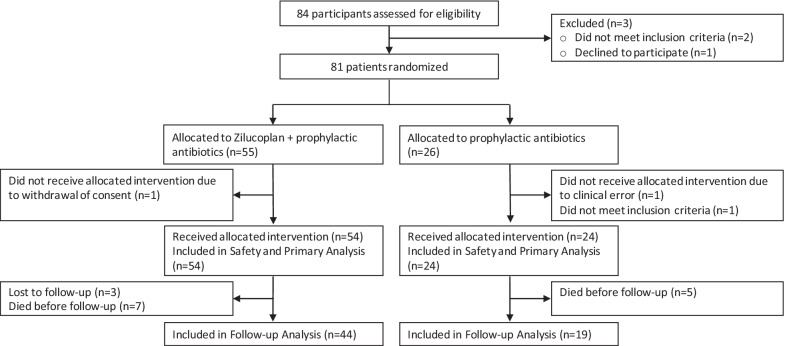
Enrollment and randomization

Between August 15th and December 16th, 2020, 81 patients were enrolled at 9 participating centers, with the last patient last visit on May 27th 2021. 55 patients were allocated to zilucoplan and prophylactic antibiotics, and 26 to prophylactic antibiotics (Fig. 1). In the zilucoplan group, 54 patients received the intervention and one withdrew consent prior to dosing. In the control group, 2 patients were excluded from the primary analysis due to one clinical error (patient did not receive any antibiotics) and one patient not meeting inclusion criteria. All patients were followed until clinical improvement or death, except for one patient with withdrawal of consent, one patient transferred to a non-participating hospital and one patient left the hospital against medical advice.

68 patients (87%) were male and 10 patients (13%) were female (Table 1). The median age was 63 years (range 35–85) and the majority of patients (87%) were Caucasian. At randomization a total of 10 patients (13%) received invasive mechanical ventilation (IMV), while 27 patients (34.6%) received either non-invasive mechanical ventilation or were breathing through a high flow oxygen delivery device. 67 patients (85.9%) were receiving glucocorticoids and 9 patients (12%) remdesivir. Biochemical signs of systemic inflammation, coagulation and complement activation were present.

**Table 1:** Characteristics of the patients at baseline according to allocated treatment

	Zilucoplan	Control	All patients
	N = 54	N = 24	N = 78
Male sex—no. (%)	49 (91)	19 (79)	68 (87)
Ethnicity—no. (%)			
African	4 (7)	0	4 (5)
Arabian	3 (6)	1 (4)	4 (5)
Asian	1 (2)	0	1 (1)
Caucasian	46 (85)	22 (92)	68 (87)
Other	0	1 (4)	1 (1)
Age at baseline—median (min, max), years	63 (35, 83)	64 (50, 85)	63 (35, 85)
BMI—mean (SD)	28 (4)	30 (4)	29(4)
Co-existing conditions—no. (%)			
Arterial hypertension	26 (48)	10 (42)	36 (46)
Diabetes mellitus	14 (26)	4 (17)	18 (23)
Cardiovascular disease	9 (17)	10 (42)	19 (24)
Chronic kidney disease	4 (7)	0	4 (5)
6-point ordinal scale at baseline—no. (%)			
2 Invasive mechanical ventilation	8 (15)	2 (8)	10 (13)
3 Non-invasive ventilation or high flow oxygen devices	19 (35)	8 (33)	27 (35)
4 Hospitalized, requiring supplemental oxygen	26 (48)	14 (58)	40 (51)
5 Hospitalized, not requiring supplemental oxygen	1 (2)	0	1 (1)
Admitted to ICU at randomisation—no. (%)	30 (56)	12 (50)	42 (54)
Days of symptoms at randomisation—median (min, max)	10 (7, 16)	10 (6, 13)	10 (6, 16)
Days of hospitalization at randomisation—median (min, max)	3 (1, 8)	2 (1, 11)	2 (1, 11)
PaO_2_/FiO_2_ ratio at baseline—mean (SD), mmHg	169 (94)	175 (93)	171 (93)
A-a gradient at baseline—mean (SD), mmHg	272 (211)	237 (208)	261 (209)
SOFA score at baseline—no. (%)			
1–2	29 (57)	14 (61)	43 (58)
3–4	14 (28)	7 (30)	21 (28)
5–6	1 (2)	2 (9)	3 (4)
7–8	7 (14)	0	7 (10)
Not done	3	1	4
Laboratory values at baseline—mean (SD)			
CRP (mg/L)	142 (89)	135 (61)	140 (81)
Lymphocyte count (10^9^/L)	0.9 (0.8)	0.7 (0.4)	0.8 (0.7)
Ferritin (µg/L)	2608 (1792)	2258 (1206)	2500 (1635)
D-dimers (ng/mL)	1100 (894)	1249 (1338)	1147 (1046)
LDH (IU/L)	479 (178)	492 (167)	483 (174)
C5 (µg/L)	114 (24)	98 (25)	110 (25)
Concomitant medication—no. (%)			
Glucocorticoids (at randomisation)	49 (91)	18 (75)	67 (86)
Glucocorticoid use (during first 28 days)	49 (91)	18 (75)	67 (86)
Anticoagulants (at randomisation)	50 (93)	22 (92)	72 (92)
Antibiotics (at randomisation)	16 (30)	2 (8)	18 (23)
Remdesivir (at randomisation)	7 (13)	2 (8)	9 (12)

Body mass index (BMI) was calculated as weight in kilograms divided by height in meters squared. Sex and ethnicity were reported by study participant. *SD* standard deviation, *CRP* C-reactive protein, *LDH* lactate dehydrogenase, *C5* complement 5, *SOFA* severity of organ failure assessment, *PaO*_*2*_ partial pressure of arterial oxygen, *FiO*_*2*_ fraction of inspired oxygen

Overall patient characteristics including comorbidities were similar. In the zilucoplan group, 15% of patients were on invasive mechanical ventilation at day of randomization compared with 8% in the control group. Seven patients in the zilucoplan arm had a SOFA score of 7 or more compared with none in the control group. More patients were on antibiotics and glucocorticoids at day of randomization in the zilucoplan group compared with the control group. In the zilucoplan group 17% of patients had prior cardiovascular disease compared with 42% in the control group.

### Primary endpoint

**Fig. 2:**
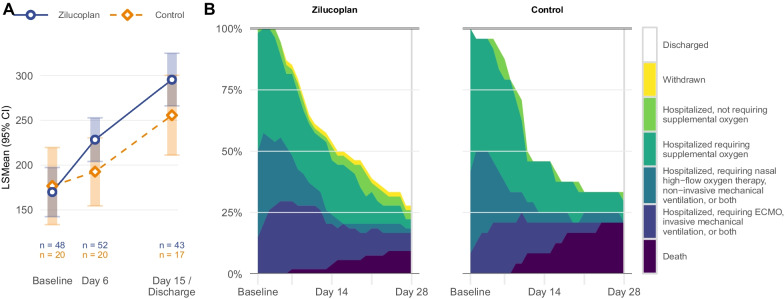
Primary outcome and secondary clinical outcome. *ECMO* extracorporeal membrane oxygenation, *FiO*_*2*_ fraction of inspired oxygen, *LSMean* Least Square Means, *PaO*_*2*_ partial pressure of arterial oxygen

The improvement in PaO_2_/FiO_2_ from baseline to day 6 had a least square mean (LSmean) change of 56.4 mmHg (95% CI 31.9 to 80.9) in the zilucoplan group and 20.6 mmHg (95% CI − 17.3 to 58.5) in the control group, corresponding to a difference between groups of 35.8 mmHg, which failed to reach statistical significance (95% CI − 9.4 to 80.9; p = 0.12) (Fig. 2 and Additional file 7: Table S1). A similar non-significant difference was seen at day 15, since the LSmean change from baseline in PaO_2_/FiO_2_ was 123.5 mmHg (95% CI 94.3 to 152.7) in the zilucoplan group and 83.7 mmHg (95% CI 39.0 to 128.4) in the control group. This corresponds to a difference in mean change from baseline to day 15 of 39.8 mmHg (95% CI − 13.6 to 93.2; p = 0.14) between groups. Similar numerical improvements were observed for the other measurements of oxygenation such as P(A-a) O_2_ gradient and A/a PO_2_ gradient, favoring the zilucoplan group over the control group but failing to reach conventional thresholds for statistical significance (Additional file 5: Fig. S1). In a Bayesian statistical analysis, the posterior probability that zilucoplan led to an improvement in oxygenation compared with the control group was > 89% for each of the three parameters at day 6 and day 15.

### Secondary endpoints

At day 28 post randomization, 5 out of 54 patients (9%) in the zilucoplan group and 5 out of 24 patients (21%) in the control group had died, corresponding with an odds ratio of 0.4 (95% confidence interval (CI) 0.1 to 1.5) (Fig. 2 and Table 2). Similar results were observed at 12–22 weeks follow-up, when 7 of 54 patients (13%) in the zilucoplan group and 5 of 24 patients (21%) in the control group had died, corresponding with an odds ratio of 0.6 (95% CI 0.2 to 2.0). In a Bayesian analysis, the posterior probability of survival in the zilucoplan group being superior to the survival in the control group was 91% at day 28 and 81% at 12–22 weeks follow-up.

**Table 2:** Mortality and safety endpoints in the safety population

	Zilucoplan	Control
	no. (%)	no. (%)
Number of patients	54	24
Mortality		
All-cause mortality at day 28	5 (9)	5 (21)
All-cause mortality at 12–22 weeks	7 (13)	5 (21)
Serious adverse events (SAEs)		
Incidence of SAEs at day 28	7 (13)	5 (21)
Incidence of SAEs at 12–22 weeks	10 (19)	5 (21)
*All SAEs leading to mortality at 12–22 weeks*		
Covid-19	4 (7)	3 (13)
Infectious disorder (not COVID-19)	2 (4)	1 (4)
Thrombosis	0	1 (4)
Multi-organ failure	1 (2)	0
All SAEs not leading to mortality at 12–22 weeks*		
Infectious disorder (not COVID-19)	3 (6)	0
Acute kidney injury	1 (2)	1 (4)
Cardiac disorder	1 (2)	0
Nosocomial or invasive fungal infection^a^		
Incidence at day 28	10 (19)	4 (17)
Adverse events		
Incidence at day 28	39 (72)	17 (71)
*Adverse events with incidence* > *10%*		
Constipation	7 (13)	5 (21)
Hypertension	8 (15)	2 (8)

*SAE* serious adverse events, *COVID-19* coronavirus disease 2019

^*^Progression and symptoms of COVID-19 were excluded from reporting

^a^Requiring treatment

No differences in evolution on 6-point ordinal scale were observed between both groups (Additional file 7: Table S1). There was a slightly faster recovery in the control group compared with the zilucoplan group in terms of duration of hospital stay and time to absence of fever and to independence of supplemental oxygen, however not statistically significant (Additional file 7: Table S1). No differences in progression to mechanical ventilation and to ARDS were observed.

At 12–22 weeks follow-up, there was a non-significant improvement towards a better result of a 6-min walk test in the zilucoplan (539.7 with standard deviation (SD) 107.7 m) compared with control (490.6 and SD 131.7 m) group (p = 0.18) (Additional file 7: Table S2). Participants did not develop lung fibrosis post COVID-19 infection, as indicated by lung function testing and imaging on follow-up (median time = 15.3 weeks) [13].

### Laboratory parameters and biomarkers

**Fig. 3:**
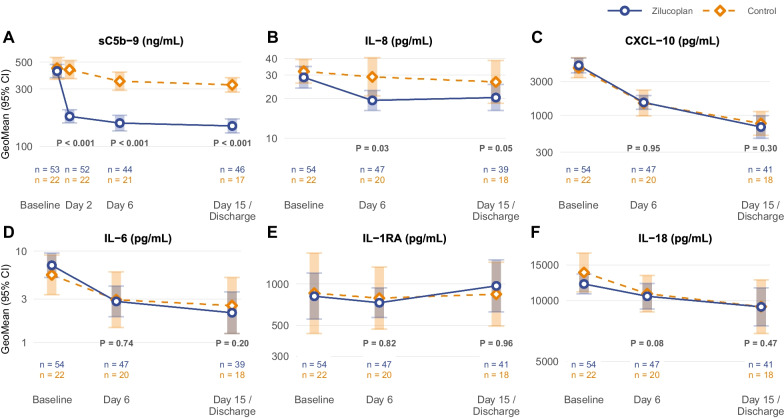
Cytokines and chemokines. *GeoMean* geometric mean. No adjustments for multiplicity were made

Zilucoplan administration was associated with a significant decrease in sC5b-9 membrane attack complexes to levels comparable to healthy controls (Fig. 3), confirming target engagement [37]. Zilucoplan administration also lowered the serum concentration of IL-8 compared with control, whereas concentrations of CXCL-10, IL-6, IL-1RA, and IL-18 were not altered (Fig. 3). Serum C-reactive protein, ferritin, lactate dehydrogenase and D-dimer levels declined over 15 days, without difference between treatment groups (Additional file 6: Fig. S2).

### Safety

C5 inhibition with zilucoplan did not lead to an increase in overall or infectious SAEs and also for the AEs the rate of in-hospital infections was similar across both treatment groups (Table 2). No Neisseria infections, serious adverse reactions (SARs) or suspected unexpected serious adverse reactions (SUSARs) occurred in this study. Overall, no unexpected safety findings and no relevant safety differences between the zilucoplan and control group were identified.

## Discussion

In this proof-of-concept phase II trial, complement C5 inhibition with zilucoplan led to numerically relevant respiratory and clinical improvements in hypoxemic COVID-19 patients with systemic inflammation, associated with a drop in sC5b-9 and IL-8 concentration, thus indicating engagement of zilucoplan on its target protein in this population with high circulating C5 levels. Despite improvements across multiple clinical oxygenation parameters at multiple time points, the group differences in mean change oxygenation parameters did not reach a priori defined statistical significance. A lower than anticipated mean baseline PaO_2_/FiO_2_ (171 mmHg measured as opposed to 320 mmHg predicted), and more than double the expected level of unavailable data (due to death, study dropout or study measures not taken) impacted the ability to detect a mean 25% improvement in oxygenation over control. In this trial no unexpected safety findings were identified when prophylactic antibiotics were administered. At odds with the encouraging effects of zilucoplan on oxygenation, there was a slightly improved median time to discharge and a faster independence from supplemental oxygen in the control group. This difference might be explained by the random allocation of more patients with higher SOFA scores at baseline in the zilucoplan group. However, more patients had survived by day 28 in the zilucoplan group, although this was non-significant since the trial was not sufficiently powered for this secondary endpoint. As a unique feature, we report long-term outcomes in our cohort, showing overall low prevalence of persistent lung abnormalities 12–22 weeks after COVID-19. On average, zilucoplan treated patients walked 49.1 m longer during a 6-min walk test at follow-up, a difference usually considered clinically significant, but failing to reach statistical significance. These promising effects of zilucoplan on multiple endpoints including survival at day 28 support further investigation of zilucoplan in larger trials with COVID-19 patients.

Sparked by enthusiasm from anecdotal case reports and non-controlled trials [30–36], several randomized controlled trials are currently investigating the effect of complement blockade in COVID-19 patients. There is significant variation between the ongoing trials, both in terms of complement target, timing of intervention and patient category targeted. A trial (NCT04369469) investigating the C5-blocking antibody ravulizumab in mechanically ventilated COVID-19 patients was stopped due to lack of efficacy, as reported by a press release [42]. Other trials with C5 blocking antibodies ravulizumab and eculizumab (NCT04346797, NCT04288713, NCT04355494, NCT04390464, NCT04570397) are still recruiting or have not yet reported results. Currently 2 additional RCT trials using zilucoplan (ACCORD trial, EudraCT 2020-001736-95; COMMUNITY trial, NCT04590568) are ongoing, which may allow for future meta-analysis.

The contribution of complement to the clinical course of COVID-19 is likely complex and varies over time [12]. Early in the disease, the complement system contributes favourably to host defence, by opsonizing and neutralizing pathogens and recruiting neutrophils and monocytes to the lung, so early complement blockade might be detrimental. In later stages, inappropriate complement activation might drive excessive inflammation and contribute to thrombo-inflammation [2, 12], and complement blockade could be beneficial [4, 6, 14]. In Covid-19 patients with at least 6 days of symptoms, zilucoplan effectively inhibited the formation of sC5b-9, most likely via blocking C5 activation in the lungs, the site most inflamed in COVID-19. It is difficult to speculate how precisely zilucoplan impacted oxygenation since we did not sample the lung compartment during treatment. The profound reduction in sC5b-9 that was observed in circulation might be a reflection of reduced MAC formation in lung capillaries, vital for gas-exchange. Most cytokines were not suppressed by zilucoplan, with the notable exception of IL-8, a chemokine released by damaged endothelial cells in response to C5b-9 complex formation on the cell surface, a process recently shown to be triggered by SARS-CoV-2-specific T cells [43]. This suggests that endothelial injury was tempered by zilucoplan. Endothelial C5b-9 deposition is a well-known trigger of microthrombus formation in various forms of thrombotic micro-angiopathy [41], although D-dimer levels were unaltered by treatment. Some clinical trials could reveal if C5 blockade also works by inhibiting C5a formation and function in COVID-19. Indeed, two large trials of anaphylatoxin C5a antibody are ongoing (NCT04333420; NCT04449588), one having reported favorable interim results with a positive trend on survival [44]. However, a phase II trial with the receptor C5aR1 antibody avdoralimab (NCT04371367) in mechanically ventilated patients was discontinued for lack of efficacy, so the precise role of C5a in COVID-19 remains unclear. One explanation is that blockade of C5aR1 alone is not sufficient to halt the detrimental effects of complement in COVID-19 since C5b-9 is still formed or because C5 can still signal through alternative receptors.

This trial has limitations. First, an open-label design was adopted given the logistical challenges at the start of the pandemic. Secondly, patients required prophylactic antibiotics for up to 14 days after the last zilucoplan dose since there was no time for vaccination against meningococci. This might have impacted outcomes. While we controlled for this bias by treating the control group with 7 days of the same prophylactic antibiotic regimen (essentially eradicating bacterial carriage in the upper airways), treatment duration was shorter. Thirdly, the trial took place in the Belgian health care setting and our patient population was predominantly composed of white men, limiting the generalization of our results. Fourthly, the parameters to evaluate oxygenation are subject to limitations. The FiO_2_ needs to be estimated based on the method of oxygen delivery and oxygen flow in patients on supplementary oxygen, which was done in a standardized manner across all participating hospitals. Finally, there were differences in baseline characteristics between groups. Less patients in the zilucoplan group had prior cardiovascular disease (17% vs 42% in control) and more patients in the zilucoplan group were on antibiotics (30% vs 8% in control) at the day of randomization. The difference in baseline antibiotic use is likely irrelevant, since all patients received ceftriaxone according to study protocol following randomization. Patients in the zilucoplan group were more severely ill as reflected by the higher SOFA score at baseline. Despite the higher organ failure scores at baseline, a Bayesian analysis indicated a lower likelihood for mortality in the zilucoplan arm.

In conclusion, this study shows safety and target engagement after administration of zilucoplan in patients with COVID-19, while failing to show statistically significant changes in mean oxygenation parameters after 6 and 15 days of treatment. Future studies in larger patient populations will have to elucidate the clinical efficacy of such treatment and evaluate if patients with more severe disease presentation or on glucocorticoids benefit more from zilucoplan treatment.

## Supplementary Information

**Additional file 1.** Study protocol.

**Additional file 2.** Statistical analysis plan.

**Additional file 3.** Overview secondary and exploratory endpoints.

**Additional file 4.** Additional statistical explanation.

**Additional file 5: Figure S1.** Primary Outcome. GeoMean, Geometric Mean; LSGeoMean, Least Square Geometric Means; LSMean, Least Square Means.

**Additional file 6: Figure S2.** Laboratory Values. Medians are represented by triangles, with an upward point for Zilucoplan and a downward point for Control, respectively. CRP, C-reactive protein; LDH, lactate dehydrogenase.

**Additional file 7: Table S1**. Primary and supportive endpoints in the full analysis data set. LSMean, least square mean; PaO_2_, arterial partial pressure of oxygen; FiO_2_, fraction of inspired oxygen; PaO_2_, partial pressure of arterial oxygen; ARDS, acute respiratory distress syndrome; CRP, C-reactive protein; CI, confidence interval; SD, standard deviation. *Based on the highest temperature in 24 h. **Table S2**. Follow-up endpoints. SD, standard deviation; DLCO, diffusing capacity of lung for carbon monoxide; HRCT, high-resolution computed tomography; WHO, world health organisation. 6-point ordinal scale: 2 on invasive mechanical ventilation; 3 on non-invasive ventilation or high flow oxygen devices; 4 hospitalized, requiring supplemental oxygen; 5 hospitalized, not requiring supplemental oxygen, 6 not hospitalized.

## Data Availability

De-identified individual participant data will be available on approval of a proposal. The shared data can be used for the analysis mentioned in the approved proposal. Proposals should be directed to the corresponding author and will be subjected to ethical assessment.

## Abbreviations

AE: Adverse event
ARDS: Acute respiratory distress syndrome
C5: Complement 5
C5aR1: Complement 5a receptor 1
CI: Confidence interval
Covid-19: Coronavirus disease 2019
CRP: C-reactive protein
CT: Computed tomography
HIRUZ: Health innovation and research institute UZ Gent
ICU: Intensive care unit
i.v.: Intravenous
LOCF: Last observation carried forward
LSmean: Least square mean
MMRM: Mixed model repeated measures
PaO_2_/FiO_2_: Partial pressure of oxygen (PaO_2_) to the fraction of inspired oxygen (FiO_2_)
REDCap: Research electronic data capture
SAE: Severe adverse event
SAR: Serious adverse reaction
SARS-CoV-2: Severe acute respiratory syndrome coronavirus 2
sC5b-9: Soluble membrane attack complex
SD: Standard deviation
SoC: Standard of care
SOFA: Severity of organ failure assessment
SUSAR: Suspected unexpected serious adverse reaction

## References

[1] Ackermann M, Verleden SE, Kuehnel M (2020). Pulmonary vascular endothelialitis, thrombosis, and angiogenesis in COVID-19. N Engl J Med.

[2] Carvelli J, Demaria O, Vely F (2020). Association of COVID-19 inflammation with activation of the C5a–C5aR1 axis. Nature.

[3] Ma L, Sahu SK, Cano M (2021). Increased complement activation is a distinctive feature of severe SARS-CoV-2 infection. Sci Immunol.

[4] Magro C, Mulvey JJ, Berlin D (2020). Complement associated microvascular injury and thrombosis in the pathogenesis of severe COVID-19 infection: a report of five cases. Transl Res.

[5] Satyam A, Tsokos MG, Brook OR, Hecht JL, Moulton VR, Tsokos GC (2021). Activation of classical and alternative complement pathways in the pathogenesis of lung injury in COVID-19. Clin Immunol.

[6] Perico L, Benigni A, Casiraghi F, Ng LFP, Renia L, Remuzzi G (2021). Immunity, endothelial injury and complement-induced coagulopathy in COVID-19. Nat Rev Nephrol.

[7] Lo MW, Kemper C, Woodruff TM (2020). COVID-19: complement, coagulation, and collateral damage. J Immunol.

[8] Java A, Apicelli AJ, Liszewski MK (2020). The complement system in COVID-19: friend and foe?. JCI Insight.

[9] Yan B, Freiwald T, Chauss D (2021). SARS-CoV-2 drives JAK1/2-dependent local complement hyperactivation. Sci Immunol.

[10] Skendros P, Mitsios A, Chrysanthopoulou A (2020). Complement and tissue factor-enriched neutrophil extracellular traps are key drivers in COVID-19 immunothrombosis. J Clin Invest.

[11] Risitano AM, Mastellos DC, Huber-Lang M (2020). Complement as a target in COVID-19?. Nat Rev Immunol.

[12] Afzali B, Noris M, Lambrecht BN, Kemper C (2021). The state of complement in COVID-19. Nat Rev Immunol.

[13] Wang EY, Mao T, Klein J (2021). Diverse functional autoantibodies in patients with COVID-19. Nature.

[14] Lam LKM, Reilly JP, Rux AH (2021). Erythrocytes identify complement activation in patients with COVID-19. Am J Physiol Lung Cell Mol Physiol.

[15] Holter JC, Pischke SE, de Boer E (2020). Systemic complement activation is associated with respiratory failure in COVID-19 hospitalized patients. Proc Natl Acad Sci U S A.

[16] Yu J, Yuan X, Chen H, Chaturvedi S, Braunstein EM, Brodsky RA (2020). Direct activation of the alternative complement pathway by SARS-CoV-2 spike proteins is blocked by factor D inhibition. Blood.

[17] Ali YM, Ferrari M, Lynch NJ (2021). Lectin pathway mediates complement activation by SARS-CoV-2 proteins. Front Immunol.

[18] Jiang Y, Zhao G, Song N (2018). Blockade of the C5a–C5aR axis alleviates lung damage in hDPP4-transgenic mice infected with MERS-CoV. Emerg Microbes Infect.

[19] Gralinski LE, Sheahan TP, Morrison TE (2018). Complement activation contributes to severe acute respiratory syndrome coronavirus pathogenesis. MBio.

[20] Zinellu A, Mangoni AA (2021). Serum complement C3 and C4 and COVID-19 severity and mortality: a systematic review and meta-analysis with meta-regression. Front Immunol.

[21] Marcos-Jimenez A, Sanchez-Alonso S, Alcaraz-Serna A (2021). Deregulated cellular circuits driving immunoglobulins and complement consumption associate with the severity of COVID-19 patients. Eur J Immunol.

[22] Lin P, Chen W, Huang H (2021). Delayed discharge is associated with higher complement C3 levels and a longer nucleic acid-negative conversion time in patients with COVID-19. Sci Rep.

[23] Henry BM, Szergyuk I, de Oliveira MHS (2021). Complement levels at admission as a reflection of coronavirus disease 2019 (COVID-19) severity state. J Med Virol.

[24] Cugno M, Meroni PL, Gualtierotti R (2021). Complement activation and endothelial perturbation parallel COVID-19 severity and activity. J Autoimmun.

[25] Alosaimi B, Mubarak A, Hamed ME (2021). Complement anaphylatoxins and inflammatory cytokines as prognostic markers for COVID-19 severity and in-hospital mortality. Front Immunol.

[26] Ramlall V, Thangaraj PM, Meydan C (2020). Immune complement and coagulation dysfunction in adverse outcomes of SARS-CoV-2 infection. Nat Med.

[27] Shen B, Yi X, Sun Y (2020). Proteomic and metabolomic characterization of COVID-19 patient sera. Cell.

[28] Valenti L, Griffini S, Lamorte G (2021). Chromosome 3 cluster rs11385942 variant links complement activation with severe COVID-19. J Autoimmun.

[29] Polycarpou A, Howard M, Farrar CA (2020). Rationale for targeting complement in COVID-19. EMBO Mol Med.

[30] Zelek WM, Cole J, Ponsford MJ (2020). Complement inhibition with the C5 blocker LFG316 in severe COVID-19. Am J Respir Crit Care Med.

[31] Urwyler P, Moser S, Charitos P (2020). Treatment of COVID-19 with Conestat Alfa, a regulator of the complement, contact activation and Kallikrein-Kinin system. Front Immunol.

[32] Mastellos DC, Pires da Silva BGP, Fonseca BAL (2020). Complement C3 vs C5 inhibition in severe COVID-19: Early clinical findings reveal differential biological efficacy. Clin Immunol.

[33] Mastaglio S, Ruggeri A, Risitano AM (2020). The first case of COVID-19 treated with the complement C3 inhibitor AMY-101. Clin Immunol.

[34] Laurence J, Mulvey JJ, Seshadri M (2020). Anti-complement C5 therapy with eculizumab in three cases of critical COVID-19. Clin Immunol.

[35] Kulasekararaj AG, Lazana I, Large J (2020). Terminal complement inhibition dampens the inflammation during COVID-19. Br J Haematol.

[36] Annane D, Heming N, Grimaldi-Bensouda L (2020). Eculizumab as an emergency treatment for adult patients with severe COVID-19 in the intensive care unit: A proof-of-concept study. EClinicalMedicine.

[37] Howard JF, Nowak RJ, Wolfe GI (2020). Clinical effects of the self-administered subcutaneous complement inhibitor zilucoplan in patients with moderate to severe generalized myasthenia gravis: results of a phase 2 randomized, double-blind, placebo-controlled, multicenter clinical trial. JAMA Neurol.

[38] Declercq J, Van Damme KFA, De Leeuw E (2021). Effect of anti-interleukin drugs in patients with COVID-19 and signs of cytokine release syndrome (COV-AID): a factorial, randomised, controlled trial. Lancet Respir Med.

[39] Rockwood K, Song X, MacKnight C (2005). A global clinical measure of fitness and frailty in elderly people. CMAJ.

[40] Harris PA, Taylor R, Thielke R, Payne J, Gonzalez N, Conde JG (2009). Research electronic data capture (REDCap)—a metadata-driven methodology and workflow process for providing translational research informatics support. J Biomed Inform.

[41] Mastellos DC, Ricklin D, Lambris JD (2019). Clinical promise of next-generation complement therapeutics. Nat Rev Drug Discov.

[42] Alexion. https://ir.alexion.com/news-releases/news-release-details/alexion-provides-update-phase-3-study-ultomirisr-ravulizumab. 2021.

[43] Georg P, Astaburuaga-Garcia R, Bonaguro L (2021). Complement activation induces excessive T cell cytotoxicity in severe COVID-19. Cell.

[44] Vlaar APJ, de Bruin S, Busch M (2020). Anti-C5a antibody IFX-1 (vilobelimab) treatment versus best supportive care for patients with severe COVID-19 (PANAMO): an exploratory, open-label, phase 2 randomised controlled trial. Lancet Rheumatol.

