# Supplementary material for: Efficacy and safety of the investigational complement C5 inhibitor zilucoplan in patients hospitalized with COVID-19: an open-label randomized controlled trial
Source: Respir Res. 2022 Aug 9;23:202. doi: 10.1186/s12931-022-02126-2 (PMC9361275; doi:10.1186/s12931-022-02126-2)
Supplement: Supplementary file 1 — Additional file 1. Study protocol. [file 12931_2022_2126_MOESM1_ESM.pdf]

A prospective, randomized, open-label, interventional study to investigate the efficacy of complement C5 inhibition with Zilucoplan® in improving oxygenation and short- and long-term outcome of COVID-19 patients with acute hypoxic respiratory failure.

Acronym / Protocol code	ZILU-COV
Protocol version and date	6.0 dd 9 June 2021
Phase	2
EudraCT n°	2020-002130-33
Sponsor	University Hospital Ghent,<br>C. Heymanslaan 10<br>9000 Ghent<br>Belgium
Financial/Material Support:	UCB

Coordinating Investigator:

Bart N. Lambrecht, MD, PhD
Department of Internal Medicine & Pediatrics,
Department of Respiratory Medicine
University Hospital Ghent
Corneel Heymanslaan
9000 Ghent, Belgium
+32/93329110

Co-investigators: Multicentric trial in Belgium

Cédric Bosteels, Catherine Van Der Straeten, Pieter Depuydt and Eva Van Braeckel contributed significantly.

A prospective, randomized, open-label, interventional study to investigate the efficacy of complement C5 inhibition with Zilucoplan® in improving oxygenation and short- and long-term outcome of COVID-19 patients with acute hypoxic respiratory failure.

Zilucoplan® in patients with acute hypoxic respiratory failure due to COVID-19 (ZILU-COV)

Protocol Co-ordinating Investigator signature page

I certify that I will conduct the study in compliance with the protocol, any amendments, GCP and the declaration of Helsinki, and all applicable regulatory requirements.

Investigator:

Name: Prof. Dr. B. Lambrecht
Function: Pulmonologist
Institution: UZ Ghent

Date: **Signature:**

A prospective, randomized, open-label, interventional study to investigate the efficacy of complement C5 inhibition with Zilucoplan® in improving oxygenation and short- and long-term outcome of COVID-19 patients with acute hypoxic respiratory failure.

Protocol Site Principal Investigator signature page

I certify that I will conduct the study in compliance with the protocol, any amendments, GCP and the declaration of Helsinki, and all applicable regulatory requirements.

Investigator:

Date: **Signature:**

Protocol Amendment History:

Version Number	Date	Description of amendment
**2.0**	18 May 2020	Section 2.1: study design added.
		Section 7.1.2: wording changed to "An Investigator must discontinue or withdraw a subject from the study for the following reason."<br>Additional reason for withdrawal from study:<br>• Cases included in the trial with a 'tentative' diagnosis of COVID-19 in which it is not possible to get a confident diagnosis by serology or other means later in the disease course, will be withdrawn from the study as soon as it is clear that SARS-CoV2 was not the cause of the clinical presentation.<br><br>Clarification added on prophylaxis for meningococcal disease: In all patients that have received Zilucoplan® as a study medication (group A patients), prophylaxis for meningococcal disease using either 3^{d} generation cephalosporin IV daily or oral ciprofloxacin daily should be continued for 14 days after the last Zilucoplan® dose, even if patient retracted from the study, or was withdrawn from the study by the investigator. In case reason for withdrawal was allergy to an antibiotic, an alternative antibiotic for prophylaxis should be given until 14 days after the last Zilucoplan® dose.
		Section 9.2, 9.4: Follow up visit week 12-22 after randomization: additional assessments were added:<br>- WHO performance scale<br>- 6 minutes walk test (per standard of care)<br>- HRCT scan to evaluate HRCT fibrosis score
		Section 9.4:<br>- assessment of vital signs: values to be collected at morning assessment, between 7-10 AM for all parameters.<br>- 6-point ordinal scale: scoring updated to "1=Death".<br><br>HRCT fibrosis score: instructions on how to score HRCT findings.
		Section 13.5: text added: A careful assessment will be performed in cases where disease related events appear to be enhanced by the IMP. In accordance with CT-3 guidance, a causality assessment will be performed for each SAE, and if the investigator considers disease related event to be IMP-related and the event is serious,

		related and unexpected, then it will be reported as a SUSAR.
		Section 13.6: Additional information on DSMB Charter: The Charter includes the role of the committee, organizational flow, committee membership, committee meetings and will be signed by all DSMB members prior to the first meeting of the DSMB.
		Section 9.3, 9.4, 10: details on samples to be collected updated, cfr. updated lab manual provided by central lab, Covance:<br>- DNA to be analysed on EDTA tube instead of PaxGene<br>- PK sample to be collected on D15/discharge<br>- serum samples: 2 x 6 ml instead of 5 ml<br>- 6 ml EDTA tube for complement added on D1, D2, D6 and D15/discharge
		Section 12.1.2 information added that no data will be collected directly into the eCRF.
		Section 11.1: primary endpoint changed to day 6, day 15 (or discharge).
		Section 6.1: information added on anti-conception requirements for male subjects.
		Section 4.2: definition of ARDS changed according to the Berlin criteria according to Jason Chertoff.
		Section 1., 5.1: definition of Hypoxic respiratory failure changed.
**3.0**	**29 May 2020**	Section 1., 3.2, 4.2: secondary objectives changed.
		Section 3.1 and 4.1 Clarification for ECMO patients added.
		Section 4.2: Definition of ARDS: bullet point 4 changed.
		Section 6.1: Definition hypoxia changed.
		Section 9.2, 9.4:<br>- laboratory assessments removed (fibrinogen and triglycerides at Day 6, 15 and FU)<br>- Score assessments removed: Clinical Sign Score, Glasgow Come Scale and NEWS2. HScore only at Day 1.<br>- Information added on timing of evaluation of 6 point ordinal scale and HScore.6 minute walk test: protocol requirement, not following SOC.<br>-
		Section 9.3:<br>- Listing of lab assessments removed.<br>- Day 4 was changed into day 6<br><br>Timepoint of analysis of cytokines changed from Day 14 to Day 15.
		General: typo's corrected.

		Section 10:<br>D1: samples of 5ml for PK was changed into 6 ml<br>One 6 ml EDTA tube was added for complement (according to flowchart)
		Section 13.4: in flowchart reporting , 'HIRUZ CTU will inform all participating sites' was added for SUSARs
		Section 9.2: clinical exam and ECG added to section "at screening", to correspond with 9.4.
**4.0**	**10 Jun 2020**	Section 3.1 and 5.1 and 7.1 and 9.4: statement/clarification on use of alternative antibiotics in case of allergy to cephalosporin or ciprofloxacin
		Section 6.2: Exclusion Criteria added: patient on ECMO at screening
		Section 8.1.4: clarification added on administration of IMP during 24 hours dialysis.
		Section 9.4: Physical Examination: to be assessed on clinical grounds, as per standard of care.<br>Clarification for ECMO patients added
**5.0**	**27 Apr 2021**	Section 4.2: Typo "≥60" corrected to "≥ 50".
		Section 4.2: Secondary endpoints updated according to SAP
		Section 4.3 has been added and describes the pharmacodynamics and pharmacokinetic endpoints
		Section 7.3, 9.4: information added on assessment of arterial blood gas: if an arterial blood gas value is available of less than 24 hours before randomization, there's no need to have a new ABG done on Day 0/1.
		Section 9.4: correction in footnote: time window of assessment of vital signs is not applicable for the follow-up visit.
		Section 13.3: Reporting address for SAEs changed to DS_ICT@ucb.com
		Section 13.7: clarification added on timelines of submission of DSUR.
		Section 10.3, 10.4: clarification added on analysis and storage by selected centres of optional samples.
		Section 10.5: identification of FAGG certified biobank to store remaining samples for future use.
		Section 9.2: volume of DNA EDTA changed from 5 ml to 4 ml (typo).
		Section 11.0: Update of statistical analysis team
		Section 5.1: "Group B" removed from sentence about prophylactic antibiotic treatment for patient randomized to Group A.
**6.0**	**9 june 2021**	Section 4.2 : All secondary endpoints not related to secondary objectives have been moved to new section 4.3 "exploratory endpoints" section.<br><br>Duplicates within secondary endpoint section have been removed.

		Removal of analytical language e.g. 'means' has been updated to reflect the endpoint itself not the analytical approach
		Section 4.3 Exploratory Endpoints was added. The content of this section concerns endpoints previously listed under section 4.2 which have been moved to the current section 4.3. For some of the endpoints, analytical language e.g. 'means' has been updated to reflect the endpoint itself not the analytical approach
		Section 4.4: clarification was added related to the exploratory nature of pharmacodynamic and pharmacokinetic endpoints
		Section 4.4: aPTT and PT have been removed from Pharmacodynamic endpoints
		Section 11.1 : name of individual UCB statistician Trevor Smart replaced by UCB
		Section 11.2: Clarification was added regarding the endpoints to be summarized.
		Section 11.2: the sentence "Data from SOC in another study with a very similar protocol **will** be incorporated into the analysis using a Bayesian frame work. This will be described more fully in the statistical analysis plan (SAP)." Has been replaced by "Data from SOC in another study with a very similar protocol **may** be incorporated into the analysis using a Bayesian frame work. This will be described more fully in the statistical analysis plan (SAP)."
		Section 12.4: The sentence "Pseudonymized biological analyses and study results will also be shared with the provider of Zilucoplan® (UCB Pharma)" has been replaced by "Pseudonymized biological analyses, **study data** and study results will also be shared with the provider of Zilucoplan® (UCB Pharma) "

TABLE OF CONTENTS

LIST OF ABBREVIATIONS 11
1. Protocol Summary 12
2. Rationale and background 14
2.1. Rationale 14
2.2. Risk/Benefit Assessment 15
2.3. Limitations 16
3. Objectives 17
3.1. Primary Objectives 17
3.2. Secondary Objectives 17
4. End Points + Time Points 18
4.1. Primary End Points + Time Points 18
4.2. Secondary End Points + Time Points 18
4.3. Pharmacodynamic and Pharmacokinetic endpoints 20
5. Study design 22
5.1. Description of study design 22
5.2. End of Study Definition 23
5.2.1. For an individual subject 23
5.2.2. For the whole study 23
5.3. Estimated duration of the study 23
6. Inclusion and Exclusion Criteria 24
6.1. Inclusion Criteria 24
6.2. Exclusion Criteria 25
6.2.1. Screen failures 25
7. Target Population 26
7.1. Subjects 26
7.1.1. Number of subjects and planned recruitment rate 26
7.1.2. Withdrawal and replacement of subjects 26
7.2. Method of recruitment 27

7.3. Screening 27
8. Investigational Medicinal Product (IMP) 28
8.1. Name of the IMP 28
8.1.1. Composition and active substance of the IMP 28
8.1.2. Producer and Distributor of the IMP 28
8.1.3. Preparation + Dosage + administration of the IMP 28
8.1.4. Permitted dose adjustments and interruption of treatment 30
8.1.5. Duration of treatment 30
8.1.6. Packaging and Labeling of the IMP 30
8.1.7. Storage conditions of the IMP 31
8.1.8. Known side effects of the medication 31
8.2. Concomitant / Rescue Medication 32
9. Study Specific Procedures 33
9.1. Randomization 33
9.2. Study specific interventions 33
9.3. Overview of collected data 35
9.4. Schematic overview of the data collection & interventions (see next page) 35
9.5. Restrictions for subjects during the study 39
10. Sampling 39
10.1. Types and number of samples 39
10.2. Timepoints of sampling 39
10.3. Sample Handling & Analysis 39
10.4. Sample Storage and/or shipping 39
10.5. Future use of stored samples 39
11. Statistical Considerations 40
11.1. Sample size calculation 40
11.2. Type of statistical methods 40
11.3. Statistical analysis team 41
12. Data handling 41
12.1. Method of data collection 41
12.1.1. Case Report Form 41
12.1.2. Data directly collected in the CRF (no source available) 41
12.2. Data storage 42
12.3. Archiving of data 42
12.4. Access to data 42
13. Safety 43

13.1. Definitions 43
13.2. Reporting requirements 44
13.2.1. AE reporting 44
13.2.2. SAE reporting 44
13.2.3. SUSAR reporting 45
13.3. List of contact details for safety reporting 45
13.4. Flowchart Reporting 46
13.5. Events, excluded from reporting 46
13.6. Data Safety Monitoring Board (DSMB) 46
13.7. Development Safety Update Report 47
14. Monitoring/Auditing/Inspection 48
14.1. Monitoring 48
14.1.1. General 48
14.1.2. Monitoring team 48
14.1.3. Scope 48
14.2. Inspection 48
14.3. Protocol Deviation policy 48
14.4. Serious breach to GCP and/or the protocol 48
15. Ethical and legal aspects 49
15.1. Good Clinical Practice 49
15.2. Informed Consent 49
15.3. Approval of the study protocol 49
15.3.1. General 49
15.3.2. Protocol amendments 50
15.4. Confidentiality and Data Protection 50
15.5. Liability and Insurance 50
15.6. End of Study Notification 50
16. Publication policy 51
17. Reference List 51

LIST OF ABBREVIATIONS

AE	=	Adverse Event
AECC	=	American-European Consensus Conference
ARDS	=	Acute Respiratory Distress Syndrome
CI	=	Coordinating Investigator
COVID-19	=	Coronavirus induced disease-2019
CT	=	Clinical Trial Unit
DSMB	=	Data Safety Monitoring Board
DSUR	=	Development Safety Update Report
EC	=	Ethics Committee
ECG	=	Electrocardiogram
eCRF	=	electronic Case Report Form
EDC	=	Electronic Data Capture
EPD	=	Electronic Patient Dossier
FAMHP	=	Federal Agency for Medicines and Health Products
FiO2	=	Fraction of inspired oxygen
FPI	=	First Patient In
FVC	=	Forced vital capacity
GCP	=	Good Clinical Practice
GDPR	=	General Data Protection Regulation
gMG	=	generalized myasthenia gravis
GMP	=	Good Manufacturing Practice
HIRUZ	=	Health, Innovation and Research Institute UZ Ghent
IB	=	Investigator's Brochure
ICF	=	Informed Consent Form
ICH	=	International Council for Harmonisation
IMNM	=	Immune Mediated Necrotic Myopathy
IMP	=	Investigational Medicinal Product
IMPD	=	Investigational Medicinal Product Dossier
LVLS	=	Last Visit, Last Subject
PCWP	=	Pulmonary Capillary Wedge Pressure
PEEP	=	Positive End Expiratory Pressure
PI	=	Principal Investigator
PaO_2	=	Partial pressure of oxygen
PNH	=	Paroxysmal Nocturnal Hemoglobinuria
SC	=	Subcutaneous
SAE	=	Serious Adverse Event
SC	=	Subcutaneous
sHLH	=	secondary hemophagocytic lymphohistiocytosis
SmPC	=	Summary of Product Characteristics
SOP	=	Standard Operating Procedure
SUSAR	=	Suspected Unexpected Serious Adverse Reaction
TERENA	=	Trans-European Research and Education Networking Association
TLC	=	Total Lung Capacity
TLS	=	Transport Layer Security

1. Protocol Summary

ZILU-COV trial : Use of Zilucoplan® in patients with acute hypoxic respiratory failure due to COVID-19

Title	A prospective, randomized, open-label, interventional study to investigate the efficacy of Zilucoplan® in improving oxygenation and short- and long-term outcome of COVID-19 patients with acute hypoxic respiratory failure.
Protocol number	ZILU-COV
Protocol version	V6.0
EudraCT number	2020-002130-33
Sponsor	University Hospital Ghent
Co-ordinating Investigator	Bart N. Lambrecht
Type of study	Interventional
Fase	Phase 2a (Proof-of-concept)
Study design	prospective, 2:1 randomized, open-label study
Study duration	22 weeks
Purpose of study	To investigate the efficacy and safety of Zilucoplan® in improving oxygenation and short- and long-term outcome of COVID-19 patients with acute hypoxic respiratory failure.
Primary Objective	To study if complement C5 inhibition on top of standard of care improves measures of oxygenation versus standard of care in patients with COVID-19
Secondary Objectives	- to study if early intervention with Zilucoplan® is safe (number of AEs/SAEs)<br>- to study if early intervention with subcutaneous (sc) Zilucoplan® affects clinical outcome defined by duration of hospital stay, 6-point ordinal scale and SOFA<br>- to study if early intervention with Zilucoplan® affects the rate of nosocomial infection<br>- to study if early intervention with Zilucoplan® affects progression to mechanical ventilation and/or ARDS<br>- to study if treatment with Zilucoplan® affects all-cause mortality rate at D28 and 12-22 weeks post-randomization<br>- to study if treatment with Zilucoplan® has a favourable effect on long term at follow up 12-22 weeks post-randomization
Primary Endpoint	Improvement of oxygenation as measured by mean and/or median change in normalized PaO2/FiO2 and P(A-a)O2 gradient between enrolment (baseline) and at predose D6 and D15 (or discharge, whichever comes first).
Number of participants	81

Study population and main inclusion criteria	Patients with confirmed COVID-19 infection<br>Presence of hypoxic respiratory failure defined as<br>1) O2 saturation below 93% on minimal 2l/min O2 therapy; and/or<br>2) PaO2/FiO2 below 350 mmHg (Strongly recommended: patient in upright position, after minimal 3 minutes without supplemental oxygen; In ventilated patients PaO2 can be taken from invasive arterial line and FiO2 taken directly from mechanical ventilation settings).
Control arm	Standard of Care (SoC) + 1 week of prophylactic antibiotics (or until hospital discharge, whichever comes first) = Group B (control)
Experimental Arm	Standard of Care (SoC) + subcutaneous Zilucoplan® + prophylactic antibiotics until 14 days after last Zilucoplan®= Group A (active)
Investigational drug, dose, route	subcutaneous Zilucoplan®, 32.4 mg once daily for 14 days or until hospital discharge, whichever comes first
Treatment duration	14 days or until hospital discharge, whichever comes first.<br>Prophylactic antibiotics will be continued for 14 days after the last Zilucoplan® administration
Follow-up duration	12-22 weeks post-randomization
Study Duration	From first patient in to final report<br>12 months

2. Rationale and background

2.1. Rationale

The proposed development plan was guided by three specific considerations:

1. Supportive Scientific Rationale:

The complement system performs a broad range of functions, including defense against invading pathogens, removal of cellular debris, modulation of adaptive immune system responses, and maintenance of homeostasis. The complement cascade is triggered by diverse mechanisms which lead to the generation of effector molecules that clear susceptible pathogens and cellular debris or promote inflammation, and is also closely regulated.

Acute respiratory distress syndrome (ARDS) is characterized by severe, acute inflammatory responses in the lung, resulting in diffuse damage to the alveolar-capillary barrier, flooding the airspaces with protein-rich edema fluid, with severe gas-exchange abnormalities and disordered ventilation-perfusion mismatch as a result, leading to profound hypoxia[1].

Among well-known causes of ARDS is viral pneumonia caused by influenza and corona viruses such as SARS-CoV, MERS-CoV and SARS-CoV2. Cytokine responses induced by these viruses can be so excessive, which is known as “cytokine storm”, that they not only enhance local inflammatory reactions in the lungs but also induce widespread inflammation elsewhere in the body. ARDS is a frequent complication of COVID-19. It is a main cause of death and the main reason for the current need of intensive care (IC) beds and ventilators to treat patients with SARS-CoV2 infection[2-4].

Excessive production of cytokines is not the only inflammatory response of the body to severe viral infections. Rather this cytokine storm is part of a broader response involving multiple inflammatory systems. Among the systems activated is the complement system. This system has been studied for decades regarding its role in ARDS. In particular the effects of activated complement on neutrophils leading to their lodging in lung capillaries where they damage the blood-air barrier by releasing oxygen radicals and aggressive proteases, are key in the pathogenesis of ARDS [5,6].

Indirect evidence suggests that complement activation likely contributes to the pathogenesis of ARDS and multiple organ failure (MOF) in COVID-19. A severe viral infection (including influenza, SARS-CoV, MERS-CoV, SARS-CoV2) can trigger a cytokine storm in the lungs and lead to a systemic hyperinflammatory response.

Studies in animal models in mice and monkeys for influenza and corona virus-induced lung injury have shown that this leads to activation of complement in the lungs, as evidenced by deposition of several complement factors in the damaged tissues, as well as to an increase of complement activation products in the circulation[7,8]. Though the exact molecular mechanism of this activation process needs to identified, it likely involves C-reactive protein (CRP) and natural IgM which bind to damaged cells, microparticles, cell debris and molecules released by damaged cells, (ie, damage-associated molecular patterns [DAMPs]), and which subsequently activate complement. The physiological role of this activation is to remove damaged cells and debris via complement receptors on phagocytes. However, in case of excessive activation, complement may do more harm than good, as it further amplifies inflammation and causes additional tissue damage. For example, C3a and C5a, which are generated during complement activation are potent inflammatory peptides, and because of these properties referred to as the anaphylatoxins. C3a and C5a can directly by interaction with their receptors C3aR and C5aR, respectively, interact with endothelial cells and mast cells to increase local vasopermeability. Secondly, C3a and in particular C5a are also chemoattractants that recruit immune effector cells which contributes to lung inflammation. Both recruited monocytes and neutrophils highly express C5aR CD88

in COVID-19 patients. Their effects on neutrophils can be deleterious as they not only recruit these cells to the lungs but also activate and degranulate these cells, leading to further damage of the endothelial-epithelial barrier of the alveoli. Finally, the anaphylatoxins, and also terminal membrane attack complex (MAC, C5b-9) can enhance cytokine responses by mononuclear cells and exacerbate the cytokine storm.

Observations in patients with viral pneumonia suggest that the progression towards ARDS is associated with complement activation. Levels of CRP and C4 were significantly increased in patients with SARS. Moreover, levels of C3a and C5a are increased in patients with 2009 H1N1 infection. Furthermore, increased levels of cleaved (presumably activated) C3 distinguish SARS patients from controls, and correlate with LDH and CRP levels. Evidence for complement activation in patients with COVID-19 has recently been documented, showing deposition of MASP2, C4 complement and C5-C9 membrane attack complex in the lungs and skin of 5 patients with severe COVID-19, 2 of which died[9]. Pathological examination suggests that complement activation might be at the heart of a thrombotic micro-angiopathy that is typically seen in COVID-19 and might be at the basis of increased LDH, thrombocytopenia, mild anemia and elevated D-dimers. The N protein of SARS-CoV2 has the potential to activate MASP-2 and in this way promote the alternative activation of complement[10]. These observations as well as those made in the animal models for virus-induced lung injury, strongly suggest involvement of complement in thrombotic events, acute lung injury, diffuse alveolar damage and ultimately development of ARDS and SIRS during COVID-19 infection.

Multiple studies in animal models not only show that virus (influenza, corona)-induced lung injury triggers activation of complement, but also that inhibition of this activation can improve outcome. Deficiency of C3, or inhibition of C5aR or C3aR by receptor antagonist or monoclonal antibody, attenuate virus-induced lung injury in various influenza and SARS models both in mice and monkeys[11,12]. Notably, these animal studies not only show an improved survival, but also decreased viral load in the lungs[7].

Though experimental evidence for involvement of complement in severe complications of COVID-19 is currently lacking, clinical observations in related syndromes and pathological descriptions of complement deposition at sites of microthrombosis in the lung and skin in COVID-19 patients and data obtained in multiple animal models for virus-induced lung injury, including injury induced by SARS-CoV and MERS-CoV support involvement of complement activation in patients with severe complications of COVID-19. Two small series in COVID-19 patients have reported initial success blocking either C3 complement using AMY101 or C5 complement using eculizumab[13] [14], and one trial is currently underway in China blocking C5a using the antibody BDB-001 (ref[10]). A genetic association study across 54 countries has identified a polymorphism in complement C3 as a major genetic risk association locus for severity of COVID-19 (Prof Joris De Lange, oral communication).

2. Experience: Use of Zilucoplan® has been performed in phase III studies

3. Expediency: Toxicology, pharmacologic and safety data supports the immediate clinical use of Zilucoplan® in hypoxic respiratory failure and ARDS due to COVID-19. Investigator brochure is available and contains detailed information on toxicity.

2.2. Risk/Benefit Assessment

COVID-19 poses a very significant risk of mortality of 3-7% and this percentage rises to mortality of 20% in patients with co-morbidity. Of all infected patients, some 15-20% develop severe respiratory symptoms necessitating hospital admission. Around 5% of infected patients will require invasive

mechanical ventilation, and many of those (40-50% will die). The current world-wide pandemic of COVID-19 is putting unforeseen stress on the entire primary, secondary and tertiary medical system, leading to unseen triage of patients that potentially benefit or not from admission to ICU units when they develop respiratory failure.

Immune protection against coronavirus infection is understood to rely primarily upon neutralizing humoral immunity and CD8 cytotoxic adaptive T cell cellular immunity, rather than innate immunity and the complement system. Therefore, C5 inhibition is not expected mechanistically to impair the immune response to coronavirus (or indeed to a vaccine once one is available).

Treatment with the approved C5 inhibitors eculizumab and ravulizumab, as well as rare genetic deficiencies in C5, are associated with a markedly increased risk for infection with encapsulated bacteria, most notably *Neisseria meningitidis and H. influenzae*, but not for viral infections generally, or for coronavirus infections in particular.

Despite the important potential risk of *Neisseria meningitidis*, no other important risks have been identified with Zilucoplan®. To prevent the risk of meningococcal disease and prevent other infections with encapsulated bacteria, patients will receive prophylactic antibiotics (e.g. intravenous 3rd generation cephalosporin while in hospital, followed by oral ciprofloxacin upon discharge until 14 days after the last Zilucoplan® administration). Antibiotics that have been used previously to prevent infections under Zilucoplan® in clinical trials include:

Antibiotic (trade)	***Usual dosage***
Cefotaxime (Claforan)	*1-2 g IV every 8 hrs*
Ceftriaxone (Rocephine, generics)	*2 g IV once daily*
Vancomycin	1 gr IV every 12 hrs,<br>*Pharmacokinetics recommended;*<br>*not recommended in monotherapy*
Ciprofloxacin (Ciproxine)	*400 mg IV every 12 hrs or*<br>*500 mg PO every 12 hrs*
Levofloxacin (Tavanic, generics)	*750 mg IV/PO daily*
Moxifloxacin (Avelox, generics)	*400 mg IV/PO every 24 hours*
Imipenem (not available in Belgium)	*500 mg IV every 6 hrs*
Meropenem (Meronem)	*1 gr IV every 8 hrs*

2.3. Limitations

There is a large number of COVID-19 infected patients that are currently being hospitalized across the globe. In just 30 days time, our COVID-19 ward at Ghent University Hospital has admitted 225 confirmed cases, of which a significant portion (30%) would have fulfilled eligibility criteria for the current proposed protocol. We believe that given the current descending part of the epidemiology curve in Belgium, with numbers of hospital-admitted patients declining rapidly, there will be some delay in obtaining high patient numbers initially. With the release of social distancing measures, and release of government-enforced quarantine measures, the number of cases may increase again after mid May 2020.

3. Objectives

3.1. Primary Objectives

Justification for our objective is that preventing progression from early acute hypoxic respiratory failure to frank ARDS via complement C5 inhibition could have a huge impact on the overflow of the ICU units, that is happening in some countries and is bound to happen on a global scale. The outcome of our study could thus have large impact from a medical, ethical and economic perspective.

The **hypothesis of the proposed intervention** is that complement C5 has profound effects on acute lung injury present in patients with severe COVID-19 and hypoxia, prior to mechanical intubation, and that C5 inhibition can lead to a 25% improvement in lung oxygenation parameters.

The **primary objective** of this intervention is to study if Zilucoplan® affects the **the median and/or mean change in oxygenation** between enrolment (baseline) and at predose day 6 and day 15 (or discharge, whichever comes first) through assessment of the PaO2/FiO2 ratio and through measurement of the P(A-a)O2 gradient, which can easily be performed in the setting of clinical observation of patients admitted to the COVID-19 ward or ICU COVID-19 unit. For patients on ECMO the PaO2/FiO2 ratio and Aa-gradient cannot be calculated as in this case the FiO2 will be missing. Therefore, the last available value of PaO2/FiO2 ratio and Aa gradient prior to ECMO can be used at that timepoint.
Comparison will be between active group A receiving Zilucoplan® on top of standard of care and control group B receiving standard of care. Prophylaxis against meningococcal disease will be given in group A in the form of IV antibiotic prophylaxis with a third generation cephalosporin (ceftriaxone or cefotaxime while in hospital, followed by oral ciprofloxacin upon discharge until at least 14 days after the last Zilucoplan® dose), since there is no time for immunization against meningococci. To control for the effects of 3d generation cephalosporin on clinical course of COVID-19, the control group B will also receive 1 week (or until hospital discharge whichever comes first) of IV 3rd generation cephalosporin. In case of allergies to these antibiotics, or on clinical indication, these antibiotics may be switched to antibiotics that also cover *Neisseria* meningitidis.

3.2. Secondary Objectives

-to study if early intervention with Zilucoplan® is safe (number of AEs/SAEs)

-to study if early intervention with Zilucoplan® affects clinical outcome defined by duration of hospital stay, 6-point ordinal scale, time to defervescence, supplemental oxygen use and SOFA score

- to study if early intervention with Zilucoplan® affects progression to mechanical ventilation and/or ARDS and duration of ICU stay

-to study if early intervention with Zilucoplan® affects the rate of nosocomial infection

-to study if treatment with Zilucoplan® has a favourable effect on long term at follow up 12-22 weeks post-randomization

-to study if treatment with Zilucoplan® affects all-cause mortality rate at day 28 and at 12-22 weeks post-randomization

4. End Points + Time Points

4.1. Primary End Points + Time Points

To measure the effectiveness of SC Zilucoplan® treatment on restoring lung homeostasis, the **primary endpoint** of this intervention is **measuring oxygenation** at predose day 6 and day 15 (or at discharge, whichever comes first) through assessment of the mean and/or median change from pretreatment (day 1) to post-treatment (day 6 and 15 or at discharge, whichever comes first) PaO_2/FiO_2 ratio, P(A-a)O_2 gradient and a/A pO_2 ratio, which can easily be performed in the setting of clinical observation of patients admitted to the COVID-19 ward or ICU COVID-19 unit. These measurements derived from an arterial blood gas sampling are taken when the patient is sitting upright and breathing room air. Since supplemental oxygen will be indispensable in some patients, the A-a gradient will be normalized against age and FiO_2 expected A-a gradient, according to validated formulas. If patients leave hospital between day 6 and day 15, that day of discharge will be taken as measurement point for primary endpoint instead of day 15. For patients on ECMO the PaO2/FiO2 ratio and Aa-gradient cannot be calculated as in this case the FiO2 will be missing. Therefore, the last available value of PaO2/FiO2 ratio and Aa gradient prior to ECMO can be used at that timepoint.

4.2. Secondary End Points + Time Points

-To study if early intervention with Zilucoplan® is safe (number of AEs/SAEs)

-**To study if early intervention with Zilucoplan® affects clinical outcome**
defined by 6-point ordinal scale change between day 1 and respectively day 6, day 15 (or discharge, whichever comes first) and day 28 (by phone call). 6-point ordinal scale defined as

1. Death
2. Hospitalized, on invasive mechanical ventilation or ECMO;
3. Hospitalized, on non-invasive ventilation or high flow oxygen devices;
4. Hospitalized, requiring supplemental oxygen
5. Hospitalized, not requiring supplemental oxygen

Not hospitalized

-Number of days requiring supplemental oxygen after randomization to Day 28 (or discharge, whichever comes first)

-Number of days with fever (defined as 37.1°C or more) during 28-day assessment period

- change from baseline in SOFA score to D6, D15 (or on discharge, whichever is first)

- Time to at least a 2-point improvement or discharge sustained up to D28 on the 6-point ordinal scale during 28-day assessment period

-Incidence of nosocomial bacterial or invasive fungal infection during 28-day assessment period
Patients with viral respiratory infection are at risk of secondary bacterial infections. As part of routine clinical care, sputum or BAL samples will be collected in patients suspected of secondary bacterial

pneumonia, and checked for the presence of bacteria. Measurements of procalcitonin levels will be performed at least three times per week until day 14 or hospital discharge.

- All-cause mortality rate at 28 days post randomization (all treated participants)

-All-cause mortality rate at 28 days post randomization (excluding participants that required invasive mechanical ventilation or ECMO, non-invasive mechanical ventilation or high-flow oxygen devices within 24 hours prior to or after randomization)

-All-cause mortality rate at 28 days post randomization (excluding participants that required invasive mechanical ventilation or ECMO within 24 hours prior to or after randomization)

-All-cause mortality rate at 28 days post randomization (only including patients that required invasive mechanical ventilation or ECMO within 24 hours prior to or after randomization)

-Incidence of patients in each category of the 6-point Ordinal Scale at follow up 12-22 weeks post-randomization

-All cause mortality at follow up 12-22 weeks post-randomization for all treated participants

- Results from 6 minute walking test

- Incidence of participants in each category of the WHO performance scale

4.3. Exploratory Endpoints

-Time since randomization until improvement in oxygenation, (defined as independence from supplemental oxygen) during 28-day assessment period (or discharge, whichever comes earlier)

-Number of days with hypoxia defined as SpO2 < 93% breathing room air or the dependence on supplemental oxygen after randomization to Day 28 (or discharge, whichever comes earlier)

-Time since randomization until absence of last fever (defined as 37.1°C or more) for more than 48h without antipyretics

- change in CRP levels between day 1 and day 6, and between day 1 and day 15 (or discharge in each case

- change in ferritin levels between day 1 and day 6, and between day 1 and day 15 (or discharge in each case).

-Incidence of SARs/SUSARs during 28 days

-Duration of hospital stay

-Duration of hospital stay in survivors

- Change from baseline in 6-point ordinal scale to Day 6, Day 15 and D28 (by phonecall) post randomization

- Percentage of participants reporting at least a 2-point improvement sustained to D28 or discharge

- AUEC/Time using the 6-point score recorded daily – up to D15, D28 and all data recorded

- Percentage of participants who record a 2-point improvement from baseline or discharge at D6, D15 and D28

- Percentage of participants not deteriorating according to the ordinal scale by 1 or 2 points on D6, D15 and D28

-6-point Ordinal Scale at 6 and 15 days (or discharge whichever comes first) and day 28 (phone call), in relation to serum D-dimers and complement C5a levels at baseline

-Time since randomization until first use of high-flow oxygen devices or non-invasive mechanical ventilation or invasive mechanical ventilation or ECMO in non-ventilated patients (i.e. excluding patients who are ventilated within 24h prior to or after randomization) during 28-day assessment period

- duration of ventilator-free days after randomization to Day 28 (or discharge, whichever comes earlier)

-Duration of invasive mechanical (including ECMO) and non-invasive mechanical ventilation in ventilated patients

-Duration of ICU stay in patients that enrolled in trial on invasive or non-invasive mechanical ventilation for less than 24h prior to or after randomization during 28-day assessment period

-Duration of ICU stay in patients that enrolled in trial that were on invasive or non-invasive mechanical ventilation or high-flow oxygen devices within 24h prior to or after randomization during 28-day assessment period

-Time since randomization to progression to ARDS
criteria-defined ARDS according to the adapted Berlin criteria as follow:

* Within 1 week of a known clinical insult or new or worsening respiratory symptoms

* bilateral infiltrates not supposed to be of cardiac origin or fluid overload

* PaO2/FiO2 < 300 mmHg

* PEEP > 5 cm H2O on invasive or non-invasive ventilation or flow ≥ 50L/min on HFOT (Optiflow)

-**Time since randomization to progression to ARDS according to D-dimers and complement C5a at randomization**

-Incidence of lung function abnormalities at follow up 12-22 weeks post-randomization

-**Incidence of lung fibrosis on chest high-resolution computed tomography (HRCT) scan at follow up 12-22 weeks post-randomization**

4.4. Pharmacodynamic and Pharmacokinetic endpoints (exploratory)

Pharmacodynamic endpoints:
Plasma and serum samples will be collected for summary and exploratory analysis as appropriate , may include but are not limited to (see also Section 9.3):

- ADA – D1 and long-term follow-up from serum samples (Summary only to indicate presence or not)
- Local safety labs such as D-dimers, LDH, ferritin, CRP, fibrinogen
- Complement system assessment (EDTA tubes) – D1, D2, D6 and D15 (or at hospital discharge, if earlier)

- Cytokines and biomarkers (from selected centres) on D1, D6 and D15 (or at hospital discharge, if earlier)
  - Cytokines may include, but not limited to:
    - IL-1beta, IL-1RA, IL-2, IL-8, IL-6, TNFa, GM-CSF, G-CSF, IP-10, MCP-1, MIP-1a, IFNg, and IL-10
  - Biomarkers may include but not limited to
    - sRAGE, Angiopoietin-2), KL6
- DNA for pharmacogenomic research (Paxgene tube, D1 only)

Pharmacokinetic endpoints:
Blood samples will be taken from participants in Arm A only on D1, D2, D6 and day 15/discharge– all pre-dose.

5. Study design

5.1. Description of study design

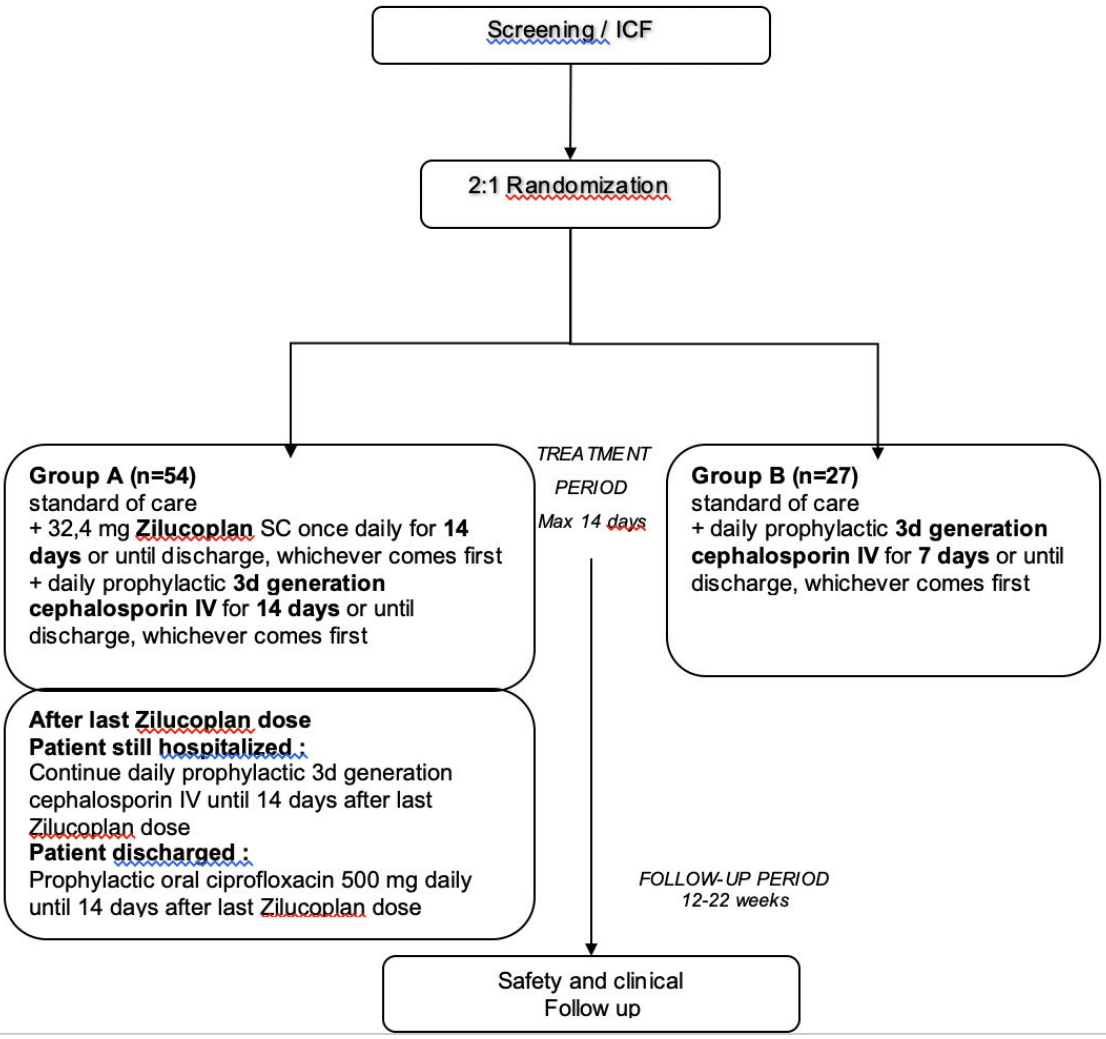

This investigator-initiated trial is a phase 2 academic, prospective, 2:1 randomized, open-label, multi-center interventional study designed to investigate the efficacy of subcutaneous Zilucoplan® in improving oxygenation and short- and long-term outcome of COVID-19 patients with acute hypoxic respiratory failure. There are currently no treatments reducing lung damage in COVID-19 patients, and no treatment that attempts to halt the progression from manageable acute hypoxic respiratory failure to acute lung injury or ARDS in patients with COVID-19 infection. **Justification for our objective** is that preventing progression from early acute hypoxic respiratory failure to ARDS could have a huge impact on the foreseeable overflow of the ICU units, that is already happening in some countries and is bound to happen on a global scale.

Several academic and non-academic centers in Belgium will participate in this trial. Study coordination, monitoring and data management will be performed under the responsibility of the Health Innovation and Research Institute UZ Ghent (HIRUZ) Clinical Trial Unit and Data Management Unit.

The **hypothesis of the proposed intervention** is that Zilucoplan® has profound effects on inhibiting acute lung injury induced by COVID-19, and can promote lung repair mechanisms, that lead to a 25%

improvement in lung oxygenation parameters. This hypothesis is based on experiments performed in mice showing that C5a blockade can prevent mortality and prevent ARDS in mice with post-viral acute lung injury.

We will randomize patients with confirmed COVID19 with acute hypoxic respiratory failure (1. O2 saturation below 93% on minimal 2l/min O2 therapy; and/or 2. PaO2/FiO2 below 350 mmHg) to receive up to 14 days of SC Zilucoplan® on top of standard of care (active group A), or to receive standard of care treatment (control group B). Randomization will be done at a 2:1 ratio active: control group. In the active group A, patients will additionally receive daily antibiotics (daily 3rd generation cephalosporin IV while in hospital, followed by oral ciprofloxacin while discharged) as primary prophylaxis against meningococcal disease until 14 days after the last dose of Zilucoplan®. Control group B will receive standard of care and prophylactic antibiotics (3rd generation cephalosporin IV) for only 1 week (or until hospital discharge whichever comes first), to control for the effects of antibiotics on the clinical course of COVID-19. In case of allergies to these antibiotics, or on clinical indication, these antibiotics may be switched to antibiotics that also cover *Neisseria* meningitidis.

To measure the effectiveness of Zilucoplan® on restoring lung homeostasis, the **primary endpoint** of this intervention is **measuring change in oxygenation parameters** comparing baseline values (pretreatment) to values predose day 6 and to values at day 15 (or discharge whichever comes first) post-randomizationin group A and group B and the differences in these values between group A and group B.

5.2. End of Study Definition

5.2.1. For an individual subject

The subject has completed the study if he or she has completed all phases of the study, including the last visit (week 12-22 post-randomization clinical follow up visit) or the last scheduled procedures, as described in this protocol (see section “9. Study Specific Procedures”).

5.2.2. For the whole study

Overall, the end of the study is reached when the last study procedure for the last subject has occurred: last subject, last visit (LSLV).
As soon as the whole study has ended (cfr. the definition above), the coordinating investigator shall notify the HIRUZ Clinical Trial Unit, so that the Competent Authority and the Ethics Committee can be informed in a timely manner according to the regulatory requirements (within 90 days after end of the study, or if the study had to be terminated early, this period must be reduced to 15 days and the reasons should clearly explained).

5.3. Estimated duration of the study

We estimate the study to terminate in 52 weeks, including last clinical follow up visits.

6. Inclusion and Exclusion Criteria

6.1. Inclusion Criteria

-Recent (≥6 days and ≤16 days of flu-like symptoms or malaise prior to randomization) infection with COVID-19.

-COVID-19 diagnosis confirmed by antigen detection test and/or PCR and/or positive serology, or any emerging and validated diagnostic laboratory test for COVID-19 within this period. For patients with a negative SARS-CoV-2 PCR and either a positive SARS-CoV-2 antigen or antibody test, the presence of suggestive lesions for COVID-19 on chest-CT scan is mandatory.

-In some patients, it may be impossible to get a confident laboratory confirmation of COVID-19 diagnosis after 24h of hospital admission because viral load is low and/or problems with diagnostic sensitivity. In those cases, in absence of an alternative diagnosis, and with highly suspect bilateral ground glass opacities on recent (<24h) chest-CT scan (confirmed by a radiologist and pulmonary physician as probable COVID-19), and a typical clinical and chemical diagnosis with signs of cytokine release syndrome, a patient can be enrolled as probable SARS-CoV-2-infected. In all cases, this needs confirmation by later seroconversion.

-Presence of hypoxia defined as
1)O2 saturation below 93% on minimal 2l/min O2 therapy;
and/or
2) PaO2/FiO2 below 350 mmHg (Strongly recommended: patient in upright position, after minimal 3 minutes without supplemental oxygen; In ventilated patients PaO2 can be taken from invasive arterial line and FiO2 taken directly from mechanical ventilation settings).

-Signs of acute lung injury and/or cytokine release syndrome defined as
ANY of the following
-serum ferritin concentration >1000 mcg/L and rising since last 24h
-single ferritin above 2000 mcg/L in patients requiring immediate high flow oxygen device (Optiflow) or non-invasive or invasive mechanical ventilation
-lymphopenia defined as <800 lymphocytes/microliter and two of the following extra criteria
-Ferritin > 700 mcg/L and rising since last 24h
-increased LDH (above 300 IU/L) and rising since last 24h
-D-Dimers > 1000 ng/mL and rising since last 24h
-CRP above 70 mg/L and rising since last 24h and absence of bacterial infection
-if three of the above are present at admission, no need to document 24h rise

-Low dose Chest CT or HRCT or Angio Chest CT scan showing bilateral infiltrates within last 2 days prior to randomization
-Admitted to specialized COVID-19 ward or an ICU ward taking care of COVID-19 patients
-Age ≥ 18 years
- Women of childbearing potential must have a negative serum pregnancy test pre-dose on day 1. Women of childbearing potential must consistently and correctly use (during the entire treatment period and 4weeks after last Zilucoplan® administration) at least 1 highly effective method for contraception.
Male subjects (who have not been surgically sterilized by vasectomy) must agree to use effective contraception during the study.
-Willing and able to provide informed consent or legal representative willing to provide informed consent

6.2. Exclusion Criteria

-Patients with known history of serious allergic reactions, including anaphylaxis, to Zilucoplan® or inability to receive antibiotic prophylaxis due to allergy to ALL of the antibiotics that can be given for prophylaxis of meningococcal disease

-History of active or past meningococcal disease

-**Invasive mechanical ventilation > 24 h at randomization**

-Patient on ECMO at screening

-Clinical frailty scale above 3 before onset of the COVID-19 episode
-Weight below 54 kg as measured max 1 week prior to inclusion
-Weight above 150 kg as measured max 1 week prior to inclusion
-Active bacterial or fungal infection
-Unlikely to survive beyond 48h
-Neutrophil count below 1500 cells/microliter
-Platelets below 50.000/microliter
-Patients enrolled in another investigational drug study
-Patients on high dose systemic steroids (> 8 mg methylprednisolone or equivalent for more than 1 month) or other moderately immunosuppressive drugs (in the opinion of the investigator) for COVID-19 unrelated disorder
-Patients on current complement inhibiting drugs
-Serum transaminase levels >5 times upper limit of normal, unless there are clear signs of cytokine release syndrome defined by LDH >300 IU/L and ferritin >700 ng/ml
-Pregnant or breastfeeding females (all female subjects deemed of childbearing potential by the investigator must have negative pregnancy test at screening)

6.2.1. Screen failures

Screen failures are defined as subjects who consent to participate in the clinical study but are not subsequently randomly assigned to the study intervention or entered in the study. A minimal set of screen failure information will be kept to ensure transparent reporting of screen failure subjects.

7. Target Population

7.1. Subjects

7.1.1. Number of subjects and planned recruitment rate

The number of subjects that will be included in this study is: 81.
These are divided into following sub-groups: 54 patients will be randomized to receive daily Zilucoplan® SC for a maximum of 14 days plus prophylactic antibiotics (3rd generation cephalosporin IV, followed by oral ciprofloxacin, or appropriate alternative) daily, maintained for at least 2 weeks after last Zilucoplan® dose)(group A); 27 patients will be randomized to receive standard of care (Group B) plus one week (or until hospital discharge whichever comes first) of 3rd generation cephalosporin IV (or appropriate alternative).

The sample size calculation is elaborated in the Statistics section in section 11.

7.1.2. Withdrawal and replacement of subjects

Subjects are free to withdraw from participation in the study at any time upon request. If patient decides to leave hospital between day 6 and day 15, treatment with Zilucoplan® will be terminated, and day of discharge will be used to calculate the day 15 endpoint.

An investigator must discontinue or withdraw a subject from the study for the following reasons:

- allergic reactions (anaphylactic shock) to Zilucoplan® or antibiotic used for prophylaxis of meningococcal disease. Allergy to an antibiotic, should lead to a switch to another antibiotic so that patients receive prophylaxis against meningococcal disease for at least 14 days after the last dose of Zilucoplan®.
- Pregnancy
- Significant study intervention non-compliance
- If any clinical adverse event (AE), laboratory abnormality, or other medical condition or situation occurs such that continued participation in the study would not be in the best interest of the subject
- If the subject meets an exclusion criterion (either newly developed or not previously recognized) that precludes further study participation
- Cases included in the trial with a 'tentative' diagnosis of COVID-19 in which it is not possible to get a confident diagnosis by serology or other means later in the disease course, will be withdrawn from the study as soon as it is clear that SARS-CoV2 was not the cause of the clinical presentation.

In all cases, the reason why subjects are withdrawn must be recorded in detail in the eCRF and in the subject's medical records.

In all patients that have received Zilucoplan® as a study medication (group A patients), prophylaxis for meningococcal disease using either 3^{d} generation cephalosporin IV daily or oral ciprofloxacin daily (or appropriate alternative) should be continued for 14 days after the last Zilucoplan® dose, even if patient retracted from the study, or was withdrawn from the study by the investigator. In case reason for

withdrawal was allergy to an antibiotic, an alternative antibiotic for prophylaxis should be given until 14 days after the last Zilucoplan® dose.

The following actions must be taken if a subject fails to return to the clinic for a required study visit (visit at 12-22 weeks post-randomization):

- The site will attempt to contact the subject and reschedule the missed visit within 4 weeks and counsel the subject on the importance of maintaining the assigned visit schedule and ascertain if the subject wishes to and/or should continue in the study.
- Before a subject is deemed lost to follow-up, the investigator or designee will make every effort to regain contact with the subject (where possible, 3 telephone calls and, if necessary, a certified letter to the subject's last known mailing address or local equivalent methods). These contact attempts should be documented in the subject's medical record or study file.
- Should the subject continue to be unreachable, he or she will be considered to have withdrawn from the study with a primary reason of lost to follow-up.

7.2. Method of recruitment

Subjects will be recruited at the COVID-19 hospitalization ward at the participating centers. The study will be proposed by the treating physician to COVID-19 subjects that fulfill inclusion and exclusion criteria.
There will be no compensation for study participation. UCB is providing Zilucoplan® to the study subjects, free of charge. Cost of prophylactic antibiotics will be part of the per patient budget per center and will be purchased by the study site pharmacy.
Since this is a hospital-based trial, in which patients are severely ill, we suspect the retention in the trial to be high.

7.3. Screening

Patients will be informed about the study by the treating physician.
After receiving full explanation, including the risk of meningococcal disease when blocking C5, having received sufficient time to considerer the trial, asking questions and receiving satisfying responses to all questions, patients will be asked to sign ICF.

A serum pregnancy test will be done (female patients of childbearing potential only).
Medical history will be checked for review of exclusion criteria and relevant subject information.

Patients will be under continuous medical supervision on the COVID-19 ward.
Exams (standard of care) include, but are not limited to:

- ECG
- Low dose chest CT-scan
- Laboratory tests for leukocyte formula, kidney and liver function, ferritin levels
- Vital signs
- Pulse oximetry
- Arterial blood gas: If an arterial blood gas value is available of less than 24 hours before randomization, there's no need to have a new ABG done on Day 0/1.

As soon as all in- and exclusion criteria are checked and patient is considered eligible, patient can be randomized in the RedCAP system. The maximal time window between screening and randomization of the patient is 24h.

8. Investigational Medicinal Product (IMP)

8.1. Name of the IMP

ZILUCOPLAN®

8.1.1. Composition and active substance of the IMP

Zilucoplan® drug product is supplied as a sterile, preservative-free solution (40 mg/mL) for subcutaneous injection

[redacted]

The prefilled syringe is placed into a Becton Dickinson (BD) UltraSafe Plus device assembled with a finger flange and plunger rod on the dose 32.4 mg (0.810 mL).

8.1.2. Producer and Distributor of the IMP

Zilucoplan® 40mg/mL (32.4 mg/0.810 mL) will be supplied by Ra Pharmaceuticals, Inc. as [redacted] placed within an administration device. Study drug will be packaged and labeled in accordance with applicable local and regulatory requirements.

8.1.3. Preparation + Dosage + administration of the IMP

Investigational drug product will be provided in prefilled syringes containing 32.4 mg of Zilucoplan® for subcutaneous injection in the abdomen (preferred site), thigh, or upper arm. This dose is equivalent to that administered to the highest weight bracket in prior weight-based dosing regimens and is expected to achieve rapid, profound, and sustained complement inhibition with acceptable safety and tolerability.

Subjects who present with very low body weight (<54kg) are excluded from enrolment. Subjects with very high body weight (>150 kg) are also excluded.

[redacted]

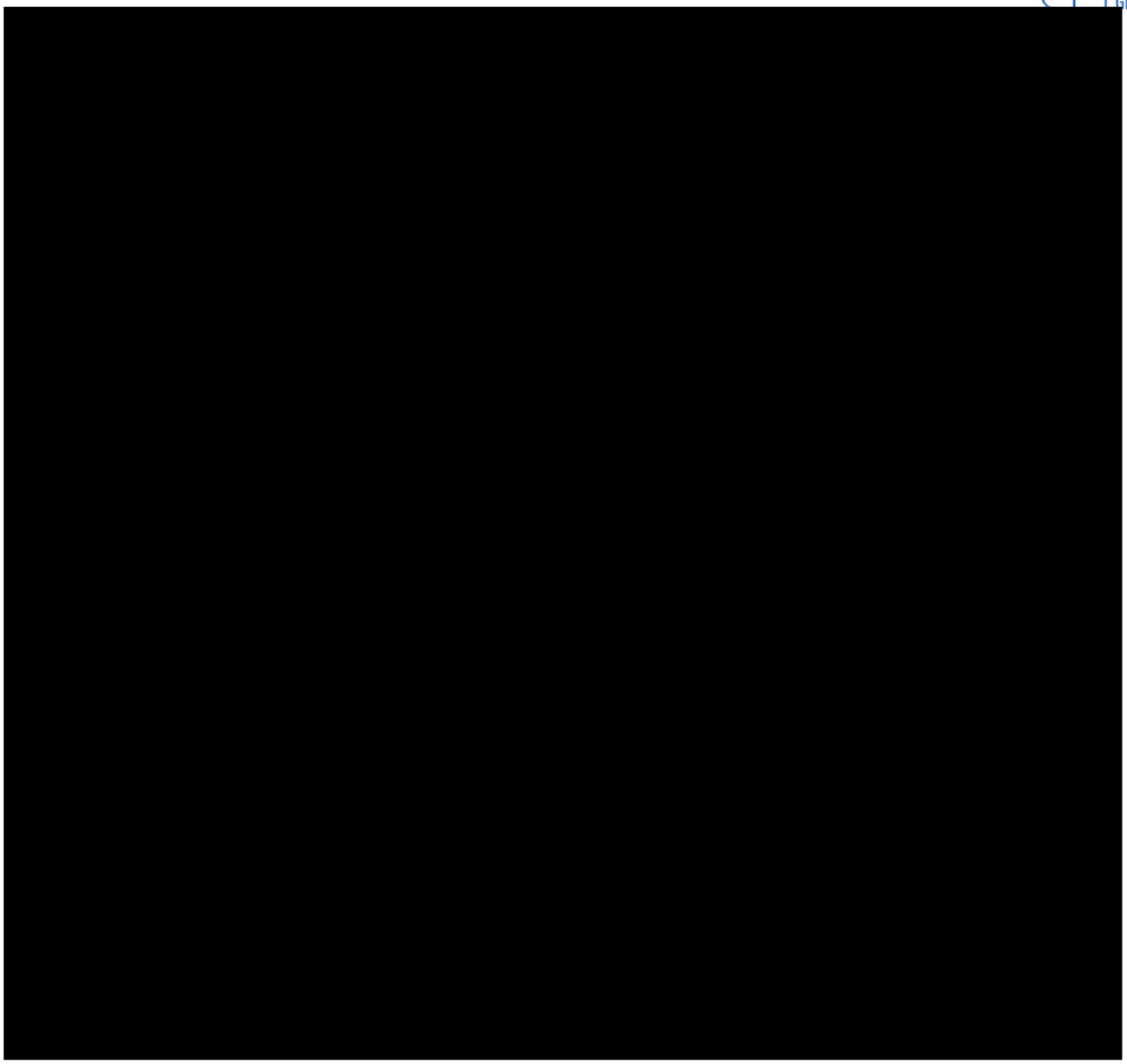

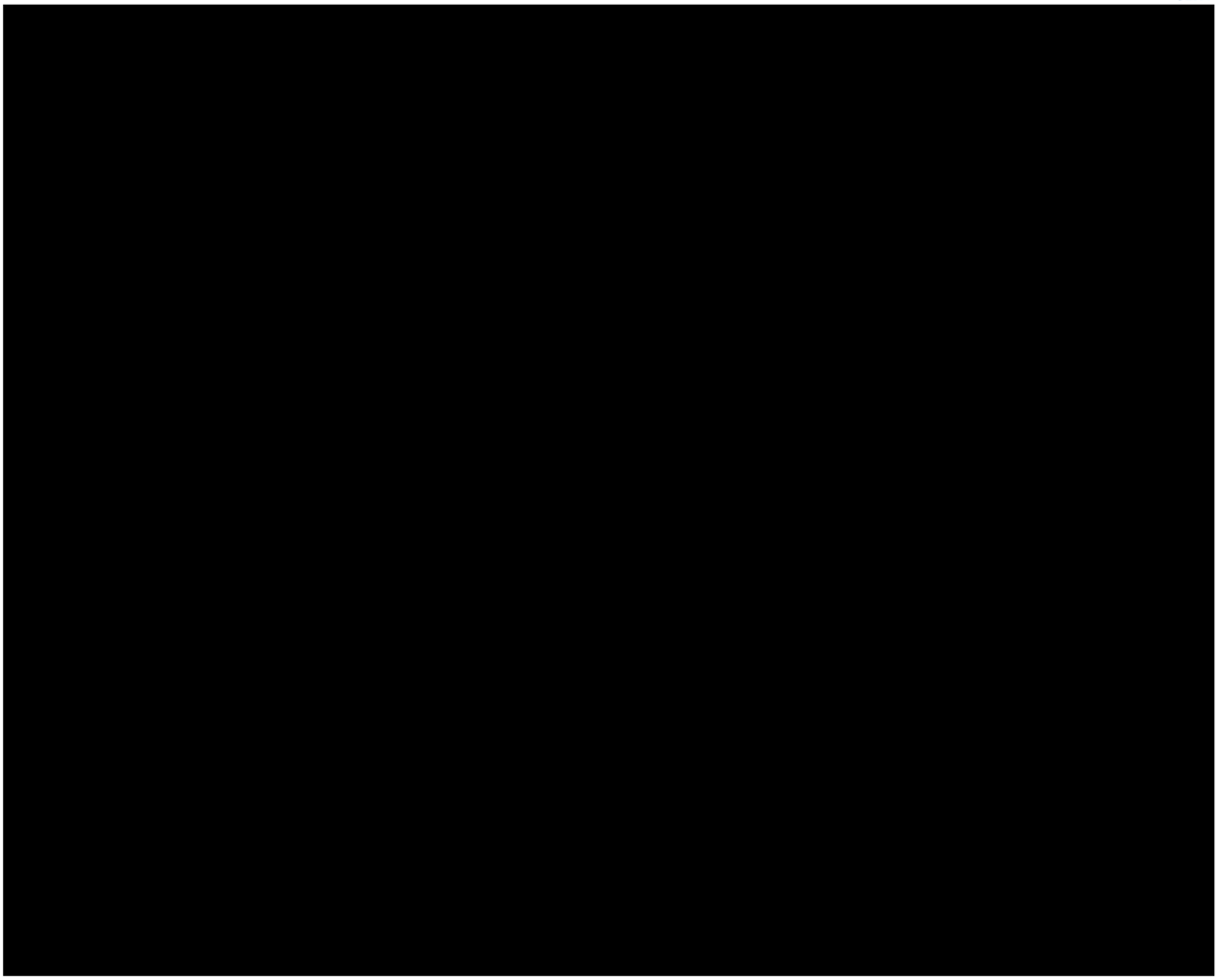

8.1.4. Permitted dose adjustments and interruption of treatment

No dose adjustments and interruptions are permitted during this trial. In case of anaphylaxis or severe AE, the drug will be immediately interrupted. In case a patient is well enough to leave hospital before 14 days of treatment, drug administration will be terminated.

Patients on hemodialysis: on days of dialysis the dose of Zilucoplan® should be administered after the procedure is completed. Since a single dose leads to complete complement inhibition within approx. 3h, subjects should continue to be fully complement inhibited even when treated with hemodialysis. If patient undergoes a 24 hours dialysis, it is recommended to administer Zilucoplan® as per protocol while dialysis is ongoing.

8.1.5. Duration of treatment

14 days, or until hospital discharge, whichever comes first

8.1.6. Packaging and Labeling of the IMP

Study drug will be packaged and labeled in accordance with applicable local and regulatory requirements. Via its partner RaPharmaceuticals, UCB had prefilled syringes ready for a Phase III trial in Myastenia gravis in various European countries, labeled on the syringe as RA101495 plus the study number identification. The syringes are too small to be individually relabeled, but the kit containing 7 syringes will be relabeled on the external packaging following communication and as per instructions

of FAMHP. All the syringes in the kit will have the same number as on the label on the corrected external packaging, making it possible to link it with the corrected trial reference and sponsor marked on the external packaging. In any case, at the various trial sites, IMP will be administered by study staff, to minimalize errors.

8.1.7. Storage conditions of the IMP

The IMP, Zilucoplan®, will be directly delivered to the study sites by UCB in a relabeled format. The study medication should be stored at 2°C to 8°C at the study site. Once dispensed to the hospital ward, Zilucoplan® may be stored at room temperature [20°C to 25°C (68°F to 77°F)] for up to 45 days protected from sources of heat, light, and damage. However, storage of Zilucoplan® outside of room temperatures should be avoided.

8.1.8. Known side effects of the medication

To date Zilucoplan® has shown a favorable safety and good tolerability profile across all studies in healthy volunteers and patients with gMG, PNH, immune mediated necrotic myopathy (IMNM), and renal impairment. No major safety risks have been identified to date, with over 100 patient years of exposure across and >40,000 injections administered in clinical trials. A difference in the overall adverse event profiles was seen between the clinical trials in different populations: few adverse events occurred in healthy controls and stable renally impaired patients; the adverse event profile in PNH reflected the hemolytic anemia characteristic of the underlying disease; and in the gMG population, many adverse events were reflective of multiple comorbidities, longstanding use of corticosteroid and immunosuppressive therapies, and the more advanced age of this population.

Although no meningococcal infections were identified with Zilucoplan® treatment to date, it is well established that inhibition of complement C5 and the terminal complement pathway increases the susceptibility to infection with encapsulated bacteria, in particular *Neisseria meningitidis*. This risk is also described in the prescribing information for the approved complement C5 inhibitor eculizumab (Soliris® USPI 2017).
Given the increased risk for *Neisseria meningitidis* infection with complement C5 inhibition or deficit, patients who have received Zilucoplan® in ambulatory clinical trials were required to have documentation of *Neisseria meningitidis* vaccination (and booster if appropriate) prior to treatment initiation. In addition, while on Zilucoplan®, subjects were monitored closely for signs and symptoms of *Neisseria meningitidis* infection, including self- monitoring based on detailed instruction about signs and symptoms of possible meningococcal infections.

Considering the acuteness of the indication and short duration of treatment (maximum of 14 days inhibition of C5 with Zilucoplan®, followed by a post-dosing inhibition of C5 for a maximum of 14 days) in the hospital, vaccination and induction of humoral immunity prior to initiation of Zilucoplan® therapy is not feasible in the context of COVID-19 with hypoxia. Vaccination during the period of COVID-19 with high fever and complement activation, is also not a safe option. Therefore, the risk of neisserial infection must be mitigated by concomitant administration of appropriate antibiotics (e.g. 3rd generation cephalosporin IV, ciprofloxacin PO) from the beginning of Zilucoplan® administration until 14 days after cessation of Zilucoplan®. In the hospital setting this will be achieved by daily IV administration of ceftriaxone (or cefotaxime). As soon as the patient is discharged, oral daily ciprofloxacin will be given until at least 14 days after the last dose of Zilucoplan®. To correct for the effects of antibiotics on the course of COVID-19 disease, the control group B will receive 1 week (or until hospital discharge whichever comes first) of IV ceftriaxone (or cefotaxime) during the hospital stay. In the control group, oral ciprofloxacin post ceftriaxone or cefotaxime is not required. In case of allergies to these antibiotics, or on clinical indication, these antibiotics may be switched to antibiotics that also cover *Neisseria* meningitidis.

8.2. Concomitant / Rescue Medication

There are no restrictions regarding concomitant/rescue medication, except for complement inhibiting drugs or other investigational drugs (see exclusion criteria).

PROPHYLACTIC ANTIBIOTICS USE

Group A patients will receive a daily IV infusion of 2g of ceftriaxone until hospital discharge (or until 28 days after randomization when still in hospital, whichever comes first). If patient is discharged before day 28 after randomization, patient will switch to oral ciprofloxacin 1x500 mg per day until at least 14 days after the last Zilucoplan® administration. In case of unavailability, ceftriaxone can be replaced by cefotaxime 1gr every 8 hours.

Group B patients will receive daily IV infusion of 2g of ceftriaxone daily IV during one week or until hospital discharge, whichever comes first. In case of unavailability, ceftriaxone can be replaced by cefotaxime 1g every 8 hours.

Prophylactic ceftriaxone will replace any amoxicillin/clavulanic acid that would be given to treat concomitant community acquired pneumonia. In the case that, on clinical grounds, antibiotics need to be switched, in all instances, *Neisseria meningitidis* and *Streptococcus pneumoniae* need to be covered by the new antibiotic regimen (e.g. meropenem or piperacillin/tazobactam). In all instances, prophylactic antimeningococcal antibiotics need to be given until at least 14 days after the last Zilucoplan® administration.

9. Study Specific Procedures

Patients will be informed about the study by the treating physician.
After receiving full explanation, having received sufficient time to considerer the trial, asking questions and receiving satisfying responses to all questions, patients will be asked to sign ICF.
The ICF process will be performed before any other study related procedure.

9.1. Randomization

In this open label trial patients will be randomized in a 2:1 ratio. Randomization in all centers will be done using REDCap (electronic IWRS system).

9.2. Study specific interventions

This is a hospital based intervention trial, in which patients with COVID-19 will be treated for a maximum of 14 days with Zilucoplan®. Patients with COVID-19 infection and respiratory failure are severely ill, and will require multiple daily clinical exams, blood sampling, vital parameter measurements, blood oxygenation measurements, and chest X-rays and/or CT. These are all part of the clinical management plan of the patients, and data stored in the electronic patient file will be used as part of the assessment of efficacy and safety profile of Zilucoplan®

At screening, a blood sample will be taken, preferentially during routine blood sampling, to determine exclusion criteria (minimally includes Ferritin, LDH, haptoglobin, D-dimers, CRP, creatinine, triglycerides, fibrinogen, eosinophils, lymphocytes, neutrophils, haemoglobin, WBC count, platelets, bilirubin, ALT and AST, procalcitonin and bHCG (females)). A clinical exam and ECG to be performed at screening or day 1 before administration of study mediation. Any form of chest CT scan within 48h before randomization (low dose, HRCT, or Angio CT scan) must document a presence of bilateral ground glass infiltrates or bilateral consolidation.

On day 1, prior to Zilucoplan® treatment in group A, and during the day in group B control patients, two tubes of blood serum (2 x 6 ml), one EDTA 6 ml tube, one 4 ml DNA EDTA tube, and two optional EDTA tubes (2 x 10 ml, optional) will be collected for measuring blood cytokine and chemokine levels, complement, and activation of immune cells. Additionally, one extra EDTA 6 ml tube will be collected from patients in the active group for pharmacokinetics (PK). In both groups, an arterial blood gas determination via arterial puncture will be taken in upright position, after minimal 3 minutes without supplemental oxygen. In case of inability to sit upright: same position should be used for all measurements of PaO2. In ventilated patients or ECMO patients ABG can be obtained from invasive arterial line. In ventilated patients the FiO2 can be directly derived from mechanical ventilation settings. The FiO2 at moment of ABG is documented in the eCRF, except for patients on ECMO.. Scores to be assessed include 6-point ordinal scale, SOFA score and HScore. Both scores to be evaluated at time of first drug administration (Arm A and Arm B).

On day 2, one EDTA 6 ml tube for patients in both groups for complement assays (prior to Zilucoplan® treatment in group A) and one tube of blood EDTA (6ml) prior to Zilucoplan® treatment in group A will be collected for pharmacokinetics.

On day 6, prior to Zilucoplan® treatment in group A (i.e. pre-dosing) and during the day in group B, two tubes of blood serum (2 x 6ml), plus 1 EDTA 6 ml tube for PK in active group, one EDTA 6 ml tube and two optional EDTA tubes (2 x 10 ml, optional) will be collected for measuring blood cytokine and chemokine levels, complement activation, and activation of immune cells. Additionally, one extra EDTA 6 ml tube will be collected from patients in the active group for PK. Routine laboratory assessment should minimally include Ferritin, LDH, haptoglobin, D-dimers, CRP, creatinine, fibrinogen,

eosinophils, lymphocytes, haemoglobin, WBC count, platelets, bilirubin, ALT and AST. Also, an arterial blood gas determination via arterial puncture will be taken in upright position, after minimal 3 minutes without supplemental oxygen. In case of inability to sit upright: same position should be used for all measurements of PaO2. In ventilated patients or ECMO patients ABG can be obtained from invasive arterial line. In ventilated patients the FiO2 can be directly derived from mechanical ventilation settings. The FiO2 at moment of ABG is documented in the eCRF, except for patients on ECMO. Scores to be assessed include 6-point ordinal scale and SOFA score

On day 15 (**or on discharge** whichever comes first), two tubes of blood serum (2 x 6 ml), 1 EDTA 6 ml tube and optional 2 EDTA tubes (2 x 10 ml, optional) will be collected for measuring blood cytokine and chemokine levels, complement activation and activation of immune cells. . Additionally, one extra EDTA 6 ml tube will be collected from patients in the active group for PK. Routine laboratory assessment should minimally include Ferritin, LDH, haptoglobin, D-dimers, CRP, creatinine, fibrinogen, eosinophils, lymphocytes, haemoglobin, WBC count, platelets, bilirubin, ALT and AST. Also, an arterial blood gas determination via arterial puncture will be taken in upright position, after minimal 3 minutes without supplemental oxygen. In case of inability to sit upright: same position should be used for all measurements of PaO2. In ventilated patients or ECMO patients ABG can be obtained from invasive arterial line. In ventilated patients the FiO2 can be directly derived from mechanical ventilation settings. The FiO2 at moment of ABG is documented in the eCRF, except for patients on ECMO. Scores to be assessed include 6-point ordinal scale and SOFA score. Measurements of procalcitonin levels will be performed at least three times per week until day 14, to exclude bacterial infection.

On days 1-14 (or until discharge, whichever comes first), patients in **group A** will receive Zilucoplan® on top of standard of care. They will additionally receive prophylactic antibiotics (IV ceftriaxone during hospital stay followed by daily ciprofloxacin for 14 days after the last Zilucoplan® administration). Patients in control **group B** will only receive one week of ceftriaxone IV (or until hospital discharge whichever comes first) on top of standard of care, to control for a modifying effect of antibiotics on the clinical course. In case patients in group A and group B would need a switch to another antibiotic for intermittent therapeutic indications, in all instances *N. meningitidis, H. Influenzae* and *S. pneumoniae* should be covered in the antibiotic spectrum of the new antibiotic. Patients in groups A or group B who are already receiving amoxicillin/clavulanic acid for treatment of possible bacterial pneumonia co-infection at randomization should be switched to IV ceftriaxone, in order for *S. pneumoniae* and *N. meningitidis* to be covered.

Upon progression of disease, need for mechanical ventilation and transfer to the ICU within the 14 day period, in patients in the active group, Zilucoplan® will be administered also via SC administration and at all instances presence of edema will be noted. If edema is noted, where possible drug will be administered subcutaneously in non-edematous area, with a preference for the upper leg.

At day 28 post-randomization (or within next 3 days) patients will be inquired by phone for 6-point ordinal scale, (S)AE, supplemental oxygen need (Y/N), therapy compliance (antibiotics) and concomitant medication since hospital discharge or day 15.

On a final clinical visit between week 12-22 after randomization two additional serum tubes (2 x 6 ml) and optional 2 EDTA tubes (2 x 10 ml) will be taken. Routine laboratory assessment should minimally include Ferritin, LDH, haptoglobin, D-dimers, CRP, creatinine, fibrinogen, eosinophils, lymphocytes, haemoglobin, WBC count, platelets, bilirubin, ALT and AST. Also, an arterial blood gas determination via arterial puncture will be taken. WHO-score, Lung function data (FVC, FEV1, FRC, RV, DLCO), a 6 minute walk test and HRCT scan to assess the HRCT fibrosis score.

9.3. Overview of collected data

1. patient demographics and biometry
   age, sex, ethnicity, day of admission, weight, length
2. day of confident or highly suspicious COVID-19 diagnosis
3. Clinical and laboratory parameters on screening day and as frequent as possible during trial
   -first day of illness (defined by myalgia, or fever, or coughing)
   -clinically relevant abnormalities found during physical examination
   -vital signs (temperature, blood pressure, heart rate, breathing rate)
   -pulse oximetry data (SpO2)
   -clinical blood gas sampling (pH, PaO2, PaCO2, HCO3, SaO2)
   -clinical chemistry sampling (will be measured on day 1, 6,day 15 or discharge and FU)
   -Chest X-ray and/or CT characteristics and radiology clinical report
4. All standard care drugs used during the trial and on day of enrolment of the trial, including oxygen flow rate.
5. Basic clinical data on prior medical history (prior lung diseases, smoking history, prior lung function measurements (preferentially within 5 preceding years), prior gas exchange measurements) and medication use will be collected from electronic medical record.
6. Study specific measurements
   Plasma and serum samples will be collected at each of the indicated timepoints for exploratory analysis that may include but not limited to:
   - ADA (Day 1, and week 12-22 follow-up)
   - Complement system assessment on EDTA tube
   - Cytokines such as IL-1beta, IL-1RA, IL-2, IL-8, IL-6, TNFa, GM-CSF, G-CSF, IP-10, MCP-1, MIP-1a, IFNg, and IL-10 as well as biomarkers (sRAGE, Angiopoietin-2) will be measured on samples collected at the various centers at day 1, day 6 and day 15 or discharge (whichever comes first)
   - DNA EDTA tube for pharmacogenomic research (Day 1 predose only)

7. Phamacokinetics (PK) measurements (predose D1, predose D2, predose D6 and D15/discharge) only in group A.

9.4. Schematic overview of the data collection & interventions (see next page)

Procedure	Screening (D0 or D1)	D1[a]	D2	D6	Hospital discharge or D15[b]	D28[c]	Follow-up[d]
Informed consent	X						
Inclusion/exclusion criteria	X						
Randomization		X					
Medical history & biometry	X						
6 minute walk test							X
Lung function							X
Physical examination[h]	→ Per SOC →						
Vital signs[e,h]	X	→ Daily until discharge from hospital →					X
Daily (S)AE inquiry		→					
Concomitant medication	X	→				X[c]	X
ECG[h]	X		On clinical grounds				
Any chest CT scan	X[p]		On clinical grounds				
HRCT[q]							X
**Routine laboratory assessments** on clinical grounds, except:<br>- D0/1, D6, discharge/D15 and FU<br>- Procalcitonin 3x/week until D15	X[f,h]			X[g,h]	X[g]		X[g]
**Study blood sampling**[h]							
- 2 x 6 ml serum tubes		X		X	X		X
- 1 x 4 ml EDTA tube (DNA)		X					
- 2 x 10 ml EDTA tube (optional, selected sites)		X		X	X		X
- 1 x 6 ml EDTA tube (complement)		X	X	X	X		
- 1 x 6 ml EDTA tube for PK (arm A only)		X	X	X	X		
- Arterial blood gas	X[h]			X[i,h]	X[i]		X
**Score assessments**							
- 6-point ordinal scale[j,h]	→ Daily until discharge from hospital →						X
- SOFA score[k,h]		X		X	X		
- HScore[l,h]		X					
- WHO performance scale[r]							X
IMP Zilucoplan® (arm A only)		→ Daily SC until D14 or discharge →					
Third generation cephalosporin (arm A only)[m]		→ Daily IV until discharge →					
Third generation cephalosporin (arm B only)[n]		→ Daily IV until D7 →					
Oral ciprofloxacin (arm A only)[o]				→ [o]			
Daily drug compliance inquiry		→				X[c]	

[a] Prior to first Zilucoplan® administration in arm A, and during the day in arm B

[b] Hospital discharge or D15, whichever comes first

[c] Inquiry by phone on D28 post-randomization (or within next 3 days) for 6-point ordinal scale, (S)AE, supplemental oxygen need (Y/N) and concomitant medication since discharge or D15

[d] Follow-up visit at 12-22 weeks post-randomization

[e] Values between 7-10AM Includes T°C (actual and highest last 24h), Pulse rate, Blood Pressure, Respiratory Rate, SpO2 by pulsoximetry, FiO2 and ventilator parameters (if patient admitted to ICU) Time window of assessment of vital signs (7-10 AM) is not applicable for follow-up visit.

[f] Minimally includes Ferritin, LDH, haptoglobin, D-dimers, CRP, creatinine, triglycerides, fibrinogen, eosinophils, lymphocytes, neutrophils, haemoglobin, WBC count, platelets, bilirubin, ALT and AST, procalcitonin and bHCG (females) (For exclusion criteria, HScore and SOFA)

[g] Should minimally include Ferritin LDH, haptoglobin, D-dimers, CRP, creatinine, fibrinogen, eosinophils, lymphocytes, haemoglobin, WBC count, platelets, bilirubin, ALT and AST (For SOFA)

[h] If arm A: Pre-dose Zilucoplan®
Arterial blood gas: If an arterial blood gas value is available of less than 24 hours before randomization, there's no need to have a new ABG done on Day 0/1.

[i] Mandatory: patient in upright position, after minimal 3 minutes without supplemental oxygen. In case of inability to sit upright: same position is to be used for all measurements of PaO2. In ventilated patients PaO2 can be taken from invasive arterial line and FiO2 taken directly from mechanical ventilation settings, document FiO2 at moment of ABG. For patients on ECMO the PaO2/FiO2 ratio and Aa-gradient cannot be calculated as in this case the FiO2 will be missing. Therefore, the last available value of PaO2/FiO2 ratio and Aa gradient prior to ECMO can be used at that timepoint.
[j] Defined as 1 = Death; 2 = Hospitalized, on invasive mechanical ventilation or ECMO; 3 = Hospitalized, on non-invasive ventilation or high flow oxygen devices; 4 = Hospitalized, requiring supplemental oxygen; 5 = Hospitalized, not requiring supplemental oxygen; 6 = Not hospitalized

[k] SOFA score (see https://www.mdcalc.com/sequential-organ-failure-assessment-sofa-score): requires PaO2, FiO2, platelet count, GCS, bilirubin, MAP and creatinine. To be evaluated between 6 and 10 am. Score to be calculated based on the worst values in the previous 24h at time of evaluation.

[l] HScore (see https://www.mdcalc.com/hscore-reactive-hemophagocytic-syndrome): requires T°C, haemoglobin, wbc count, platelets, ferritin, fibrinogen, triglycerides and AST (BM aspirate is not required) To be evaluated at time of randomization.

[m] Third generation cephalosporin replaces empiric amoxicillin/clavulanic acid to treat concomitant community acquired pneumonia. In case of allergies or clinical indication for antibiotic switch, *N. menigitidis* and *S. pneumoniae* need to be covered by the new antibiotic treatment (e.g. meropenem or piperacilin/tazobactam). Third generation cephalosporin is given until D28 or hospital discharge whichever comes first.

[n] Group B patients receive daily injection of 2g ceftriaxone IV (or appropriate alternative in case of allergies or on clinical indication) during one week or until hospital discharge, whichever comes first.

[o] Upon discharge before D28 post-randomization, patients will switch to daily oral ciprofloxacin (500 mg 1x/d) until at least 14 days after the last dose of Zilucoplan® (only in arm A).

[p] any form of chest CT scan within 48h before randomization (low dose, HRCT, or Angio CT scan) must document a presence of bilateral ground glass infiltrates or bilateral consolidation

[q] HRCT scoring: A subjective assessment of the overall extent of normal attenuation, reticular abnormalities, honeycombing and traction bronchiectasis will be performed. A reticular abnormality is defined as a collection of innumerable areas of small linear opacity. Honeycombing is defined as the presence of a cystic airspace measuring 3‑10 mm in diameter, with 1- to 3-mm thick walls. Traction bronchiectasis is defined as irregular bronchial dilatation within the surrounding areas showing parenchymal abnormalities. The morphological criteria on HRCT scans include bronchial dilatation with respect to the accompanying pulmonary artery, a lack of tapering of the bronchi and the identification of bronchi within 10 mm of the pleural surface. The HRCT findings will be graded on a scale of 1‑4 based on the classification system: 1. normal attenuation; 2. reticular abnormality; 3. traction bronchiectasis; and 4. honeycombing. The presence of each of the above four HRCT findings will be assessed independently in three (upper, middle and lower) zones of each lung. The upper lung zone is defined as the area of the lung above the level of the tracheal carina, the lower lung zone is defined as the area of the lung below the level of the inferior pulmonary vein and the middle lung zone is defined as the area of the lung between the upper and lower zones. The extent of each HRCT finding will be determined by visually estimating the percentage (to the nearest 5%) of parenchymal involvement in each zone. The score for each zone will be calculated by multiplying the percentage of the area by the grading scale score. The six zone scores will be averaged to determine the total score for each patient. The highest score is 400 points and the lowest score is 100 points using this calculation method. The total score is the “HRCT fibrosis score”. (https://www.ncbi.nlm.nih.gov/pmc/articles/PMC3922654/pdf/1465-9921-15-10.pdf)

[r] WHO performance scale: 0. Able to carry out all normal activity without restrictions; 1. Restricted in physically strenuous activity but ambulatory and able to carry out light work; 2. Ambulatory and capable of all self-care but unable to carry out any work: up and more than 50% of waking hours; 3. Capable of only limited self-care: confined to bed or chair more than 50% if waking hours; 4. Completely disabled: cannot carry on any self-care: totally confined to bed or chair.

9.5. Restrictions for subjects during the study

There are no subject restrictions during this trial.

10. Sampling

10.1. Types and number of samples

D1: pre-dose serum blood sample 2 x 6ml serum, one EDTA 6ml tube for PK in active group A, one 4 ml DNA EDTA tube, one EDTA 6 ml tube for complement and optional EDTA blood sample, 2 x 10 ml
D2: predose 1 x 6ml EDTA tube for PK in active group A only, 1 x 6ml EDTA tube for patients in both groups
D6: serum blood sample 2 x 6ml serum tube, one 6 ml EDTA tube for PK in group A, 1 x 6ml EDTA tube for patients in both groups and optional EDTA blood sample, 2 x 10 ml
D15 or at discharge: serum blood sample 2 x 6ml, one 6 ml EDTA tube for PK in group A, 1 x 6ml EDTA tube for patients in both groups and optional EDTA blood sample, 2 x 10 ml
Week 12-22 post-randomization follow-up visit: serum blood sample 2 x 6ml, optional EDTA blood sample, 2 x 10 ml

10.2. Timepoints of sampling

These samples are to be taken predose on D1, D2, D6 and D15 (or discharge whichever comes first) and on final follow up visit between week 12 and 22.

10.3. Sample Handling & Analysis

Samples will be taken during hospitalization preferably together with the blood draw for standard of care and will be used for measurements of pharmacodynamics and pharmacokinetic endpoints described under section 4.3

For sample handling and analysis, see lab manual provided by Covance.

OPTIONAL sampling per center

In selected centers (to be decided by the local investigator after consulting with the coordinating principle investigator), additional 2 x 10 ml EDTA blood tubes will be taken at day 1, 6 and 15, or at hospital discharge whichever comes first and safety follow-up, for flow cytometric analysis and COVID-19 related research purposes. The samples will be stored in the FAGG-certified biobank mentioned in section 10.5 until further use in the context of COVID-19 related research takes place.

Separate submission to the Ethics Committee will be initiated before execution of any research activity on the blood samples stored in the biobank.

10.4. Sample Storage and/or shipping

See lab manual provided by Covance.
Optional EDTA samples (selected sites) to be shipped to UZ Ghent for analysis and storage.

10.5. Future use of stored samples

Initially samples will be stored for the use as described within this protocol. If at a later time point samples will be stored for future use, they will be stored in the FAGG certified biobank “prospectieve biobank CF, bronchiëctasieën en respiratoire infecties” managed by biobank manager Prof. Dr. E. Van Braeckel. For each future project with samples collected from the current protocol, a separate Ethics Committee approval will be obtained.

11. Statistical Considerations

11.1. Sample size calculation

The outcome(s) on which the sample size calculation is based upon, is the primary endpoint measurement of oxygenation, defined as ratio of PaO2/FiO2.

Sample calculation and power analysis have been performed by UCB. The target difference is the difference measured at the primary endpoint (day 6 and day 15 or discharge) between the control and the treated group. Given a sample size of 81 patients, 54 on Zilucoplan® and standard of care and 27 on standard of care there would be >85% power to show a significant difference from standard of care at the 2 sided 5% level if the true underlying treatment difference was an 80 point difference (25% of 320, being the mean at hospital admission) in the PaO2/FiO2 ratio. This assumes a standard deviation of 105 and a dropout rate of less than 10%.

11.2. Type of statistical methods

All primary and secondary endpoints will be summarized and where relevant represented graphically

Primary endpoint:
The change from baseline in PaO2/FiO2 ratio will be analyzed using a Mixed Model Repeated Measures (MMRM) analysis with a fixed effect for Baseline PaO2/FiO2 ratio, treatment, day and treatment by day interaction, where day is categorical. Subject will be fitted as a random effect and an unstructured covariance will be used unless it fails to converge, where other covariance structures will be considered. Other covariates, such as gender, may also be included in the model, if by the time of the analysis these have been identified as key differentiators in terms of COVID-19 recovery. If the variability is shown to be inappropriate for this additive model, then a log transformation of the PaO2/FiO2 ratio may be considered.

Data from SOC in another study with a very similar protocol may be incorporated into the analysis using a Bayesian frame work. This will be described more fully in the statistical analysis plan (SAP).

A similar analysis for the P(A-a)O2 gradient will be completed

Other Endpoints:
Other continuous endpoints will be analyzed in a similar way using an MMRM.
Response rates, such as 28 day all-cause mortality a logistic regression will be used.
Count data will use a generalized linear model with a log link unless the model is either over or under dispersed and then an alternative approach will be used. If it is counts out of a fixed number of days, then a logistic regression will be considered.

For the final follow up, a survival analysis with Kaplan-Meir plots will be created covering from Day 1 to end of follow up.

The detail around the analyses will be explained in more detail in the SAP and as this is an exploratory study, additional analyses may be considered to explore the data more fully.

Interim analyses: will be performed when 42 patients have reached primary endpoint analysis.

After half the patients have completed day 15 an interim analysis will be completed to understand if there is already enough evidence to prompt further work on COVID-19 treatment with Zilucoplan®. There is no plan to stop the study at this stage.
Analyses will also be completed when all patients have finished day 28 to assess all pre-follow up assessments and 28 day mortality (via phone call). This will enable key results to be understood as soon as possible without compromising the study.

11.3. Statistical analysis team

The statistical analysis will be performed by UCB.

12. Data handling

12.1. Method of data collection

Subjects that are included in the study at Ghent University Hospital or other Belgian centers, will be assigned a unique study number upon their registration in REDCap. On all documents submitted to the coordinating center, sponsor or CI, patients will only be identified by their study number. The subject identification list will be safeguarded by the site. The name and any other directly identifying details will not be included in the study database.

12.1.1. Case Report Form

An electronic data capture (EDC) system, i.e. REDCap, will be used for data collection. Data reported on each eCRF should be consistent with the source data. If information is not known, this must be clearly indicated on the eCRF. All missing and ambiguous data will be clarified.
Only the data required by the protocol are captured in the eCRF. The eCRFs and the database will be developed, based on the protocol. The final eCRF design will be approved by the Co-ordinating Investigator.
All data entries and corrections will only be performed by study site staff, authorized by the investigator. Data will be checked by trained personnel (monitor, data manager) and any errors or inconsistencies will be clarified. The investigator must verify that all data entries in the eCRF are accurate and correct.

REDCap is provided and maintained by Vanderbilt University; a license for use was granted to the Health, Innovation and Research Institute (HIRUZ). REDCap is a web-based system intended for academic clinical trials.
The study site staff is responsible for data entry in REDCap.

12.1.2. Data directly collected in the CRF (no source available)

All data is to be collected in accordance with the latest version of the ICH E6 (R2) GCP guidelines. There will be no data directly collected in the CRF.

12.2. Data storage

The data is accessed through a web browser directly on the secure REDCap server. The server is hosted within the UZ Ghent campus and meets hospital level security and back-up requirements.

Privacy and data integrity between the user's browser and the server is provided by mandatory use of Transport Layer Security (TLS), and a server certificate issued by TERENA (Trans-European Research and Education Networking Association). All study sites will have access to REDCap. Site access is controlled with IP restriction.

12.3. Archiving of data

The investigator and sponsor specific essential documents will be retained for at least 25 years. At that moment, it will be judged whether it is necessary to retain them for a longer period, according to applicable regulatory or other requirement(s).

12.4. Access to data

Direct access will be granted to authorised representatives from the Sponsor, host institution and the regulatory authorities to permit study-related monitoring, audits and inspections.

Pseudonymized biological analyses, study data and study results will also be shared with the provider of Zilucoplan® (UCB Pharma)

Login in REDCap is password controlled. Each user will receive a personal login name and password and will have a specific role which has predefined restrictions on what is allowed in REDCap. Furthermore, users will only be able to see data of subjects of their own site. Any activity in the software is traced and transparent via the audit trail and log files.

13. Safety

13.1. Definitions

Term	Definition
**Adverse Event (AE)**	Any untoward medical occurrence in a subject to whom a medicinal product has been administered, including occurrences which are not necessarily caused by or related to that product. An AE can therefore be any unfavourable and unintended sign (including an abnormal laboratory finding), symptom, or disease temporally associated with the use of a medicinal (investigational) product, whether or not related to the medicinal (investigational) product.
**Unexpected Adverse Event**	An adverse event, the nature or severity of which is not consistent with the applicable product information (e.g., Investigator's Brochure for an unapproved investigational product or package insert/summary of product characteristics for an approved product).
**Adverse Reaction (AR)**	An untoward and unintended response in a subject to an investigational medicinal product which is related to any dose administered to that subject.<br>The phrase "response to an investigational medicinal product" means that a causal relationship between a study medication and an AE is at least a reasonable possibility, i.e. the relationship cannot be ruled out.<br>All cases judged by either the reporting medically qualified professional or the Sponsor as having a reasonable suspected causal relationship to the study medication qualify as adverse reactions.
**Serious Adverse Event (SAE)**	A serious adverse event is any untoward medical occurrence that:<br>• results in death<br>• is life-threatening<br>• requires inpatient hospitalisation or prolongation of existing hospitalisation<br>• results in persistent or significant disability/incapacity<br>• consists of a congenital anomaly or birth defect<br><br>Other 'important medical events' may also be considered serious if they jeopardise the subject or require an intervention to prevent one of the above consequences.<br>NOTE: The term "life-threatening" in the definition of "serious" refers to an event in which the subject was at risk of death at the time of the event; it does not refer to an event which hypothetically might have caused death if it were more severe.
**Serious Adverse Reaction (SAR)**	An adverse event that is both serious and, in the opinion of the reporting Investigator, believed with reasonable probability to be due to one of the study treatments, based on the information provided.
**Suspected Unexpected Serious Adverse Reaction (SUSAR)**	A serious adverse reaction, the nature and severity of which is not consistent with the information about the medicinal product in question set out:<br>• in the case of a product with a marketing authorisation, in the summary of product characteristics (SmPC) for that product<br>• in the case of any other investigational medicinal product, in the investigator's brochure (IB) relating to the study in question

Attribution definitions
An adverse event is considered associated with the use of the drug if the attribution is possible, probable or definitive, in the opinion of the investigator.

Not related
An adverse event which is not related to the use of the drug.

Unlikely
An adverse event for which an alternative explanation is more likely - e.g. concomitant drug(s), concomitant disease(s), and/or the relationship in time suggests that a causal relationship is unlikely.

Possible
An adverse event which might be due to the use of the drug. An alternative explanation - e.g. concomitant drug(s), concomitant disease(s), - is inconclusive. The relationship in time is reasonable; therefore the causal relationship cannot be excluded.

Probable
An adverse event which might be due to the use of the drug. The relationship in time is suggestive (e.g. confirmed by dechallenge). An alternative explanation is less likely - e.g. concomitant drug(s), concomitant disease(s).

Definitely
An adverse event which is listed as a possible adverse reaction and cannot be reasonably explained by an alternative explanation - e.g. concomitant drug(s), concomitant disease(s). The relationship in time is very suggestive (e.g. it is confirmed by dechallenge and rechallenge).

13.2. Reporting requirements

13.2.1. AE reporting

AE's will be recorded from the first drug administration until the end of the study, as defined in section 5.2.
Special attention will be given to those subjects who have discontinued the study for an AE, or who experienced a severe or a serious AE. All AE's should be recorded in the patient's file and in the CRF.

13.2.2. SAE reporting

SAE's occurring during the entire study period will be reported as below.

All serious adverse events (initial and follow up information) and pregnancies occurring during this study must be reported by the local Principal Investigator within 24 hours after becoming aware of the SAE to:

- The local ethics committee (it is the responsibility of the local PI to report the local SAE's to the local EC)
- HIRUZ CTU of the University Hospital Ghent
- The National Coordinating Investigator (in case of multicenter studies)
  The company UCB that provides the IMP

This reporting is done by using the appropriate SAE form. For the contact details, see below.

13.2.3. SUSAR reporting

In case the Coordinating Investigator, in consultation with HIRUZ CTU, decides the SAE is a SUSAR (Suspected Unexpected Serious Adverse Reaction), HIRUZ CTU will report the SUSAR to the Central EC and the FAMHP within the timelines as defined in national legislation. The Coordinating Investigator reports the SUSAR to all local PI's.
In case of a life-threatening and fatal SUSAR the entire reporting process must be completed within 7 calendar days. In case of a non life-threatening SUSAR the reporting process must be completed within 15 calendar days.

13.3. List of contact details for safety reporting

Contact details for serious adverse drug reactions and serious adverse events:

HIRUZ CTU:
Ghent University Hospital
C. Heymanslaan 10, 1K5
9000 Ghent, Belgium
e-mail: hiruz.ctu@uzgent.be
Tel: +32 9 332 05 00
Fax: +32 9 332 05 20

Coordinating Investigator:
Prof. dr. Bart Lambrecht
Ghent University Hospital
Department of pneumology
C. Heymanslaan 10, 1K5
9000 Ghent, Belgium
email: bart.lambrecht@ugent.be
Tel: +32 9 332 91 10

Marketing Authorisation Holder:
N.A.

Partner providing the IMP (serious adverse drug reactions only):
DS_ICT@ucb.com

13.4. Flowchart Reporting

Type of Adverse Event	*Action to be taken*
AE	List all AE's per subject in the patient's file and add this information to the CRF.
SAE	Notify to HIRUZ CTU within 24 hours after becoming aware of the SAE + add the SAE to a list that will be reported yearly (see section 13.8)
SAR	Notify to HIRUZ CTU within 24 hours after becoming aware of the SAE<br>→ HIRUZ CTU will submit to the central EC<br>→ HIRUZ CTU informs company that provides the IMP
SUSAR	Notify to HIRUZ CTU within 24 hours after becoming aware of the SUSAR<br>→ HIRUZ CTU will submit to the central EC.<br>→ HIRUZ CTU will submit to the FAMHP<br>→ HIRUZ CTU informs company that provides the IMP<br>→HIRUZ CTU will inform all participating sites

In case the (SU)SAR occurs at a local participating site, the local PI or study team should also contact:

- The local Ethics Committee
- The Co-ordinating Investigator

13.5. Events, excluded from reporting

COVID-19 infection is a very recent syndrome, on which few data are available. Normal symptoms and natural disease course symptoms that will not be reported as adverse events are dyspnea, coughing, malaise, fever, drop in oxygen saturation, progression to respiratory failure, progression to ARDS, drop in blood pressure. However, progression to multi-organ failure, may have other etiologies and will be reported as SAE.

A careful assessment will be performed in cases where disease related events appear to be enhanced by the IMP. In accordance with CT-3 guidance, a causality assessment will be performed for each SAE, and if the investigator considers disease related event to be IMP-related and the event is serious, related and unexpected, then it will be reported as a SUSAR.

13.6. Data Safety Monitoring Board (DSMB)

Considering the known safety profile of the study medications and study design, however in a new disease indication, a DSMB (chaired by Prof. Sylvie Rottey) is foreseen after enrolment of the first 10 active patients in the trial. The Charter includes the role of the committee, organizational flow, committee membership, committee meetings and will be signed by all DSMB members prior to the first meeting of the DSMB.

13.7. Development Safety Update Report

The Coordinating Investigator will provide DSURs once a year throughout the entire duration of the clinical study, or on request, to the Competent Authority, Central Ethics Committee and Sponsor. This DSUR will include all SAE's (who were not categorized as SAR's and were not immediately reported to the EC).

The report will be submitted 1 year (+ maximum 60 days) after the 'Development International Birth Date (DIBD)', and will subsequently be submitted each year until the study is declared ended. This DIBD is the date of the sponsor's first overall authorisation to conduct the clinical trial in any country worldwide.

HIRUZ CTU can provide a template that can be used to complete this DSUR.

14. Monitoring/Auditing/Inspection

14.1. Monitoring

14.1.1. General

Monitoring of the study will be performed in compliance with GCP E6(R2) and the applicable regulatory requirements. The study team will be trained in an initiation visit by the monitor. A training and delegation log will be held. A detailed description of the monitoring tasks can be found in the latest version of the (study-specific) 'Monitoring plan'.

14.1.2. Monitoring team

Monitoring services will be provided by HIRUZ CTU. All relevant contact details (e.g. primary contact person, can be found in the 'Monitoring plan'.

14.1.3. Scope

Monitoring services will consist of the following (non-exhaustive list):
- review of informed consents and the followed process
- check on recruitment status
- checking for protocol deviations/violations
- checking GCP compatibility
- check on safety reporting compliance
- IMP handling and storage
- review of study data

…

14.2. Inspection

This study can be inspected at any time by regulatory agencies during or after completion of the study. Therefore access to all study records, including source documents, must be accessible to the inspection representatives. Subject privacy must be respected at all times, in accordance to GDPR, GCP and all other applicable local regulations.
The investigator/study team should immediately notify the sponsor if he or she has been contacted by a regulatory agency concerning an upcoming inspection.

14.3. Protocol Deviation policy

Sponsor and all investigators agree to take any reasonable actions to correct protocol deviations/violations noted during monitoring/inspection, in consultation with the monitoring team. All deviations must be documented on a protocol deviation log by the study team that is kept available at any time for monitoring/inspection purposes. Under emergency circumstances, deviations from the protocol to protect the rights, safety or well-being of human subjects may proceed without prior approval of the sponsor and the EC.

14.4. Serious breach to GCP and/or the protocol

Critical issues that significantly affect patient safety, data integrity and/or study conduct should be clearly documented and will be communicated with the Coordinating Investigator, HIRUZ CTU and possibly both the applicable Ethics Committee(s) and Competent authority. (Please contact HIRUZ CTU asap in case of a serious breach: hiruz.ctu@uzgent.be and/or +3293320500).
Early determination of the study (in a specific center or overall) may be necessary in case of major non-compliance.

15. Ethical and legal aspects

15.1. Good Clinical Practice

The study will be conducted cfr the latest version of the ICH E6 (R2) GCP guidelines, creating a standard for the design, conduct, performance, monitoring, auditing, recording, analyses and reporting of clinical studies that provides assurance that the data and reported results are accurate and that the rights, integrity and confidentiality of study subjects are protected.

15.2. Informed Consent

Eligible subjects may only be included in the study after providing written (witnessed, if needed) Ethics Committee-approved informed consent, or, if incapable of doing so, after such consent has been provided by a legally acceptable representative(s) of the subject.
Informed consent must be obtained before conducting any study-specific procedures (as described in this protocol).

Prior to entry in the study, the investigator must explain to potential subjects or their legal representatives the study and the implication of participation. Subjects will be informed that their participation is voluntary and that they may withdraw consent to participate at any time. Participating subjects will be told that their records may be accessed by competent authorities and by authorized persons without violating the confidentiality of the subject, to the extent permitted by the applicable law(s) and/or regulations. By signing the Informed Consent Form (ICF), the subjects or legally acceptable representatives are authorizing such access.
After this explanation and before entry to the study, written, dated and signed informed consent should be obtained from the subject or legally acceptable representative. The ICF should be provided in a language sufficiently understood by the subject. Subjects must be given the opportunity to ask questions.
The subject or legally acceptable representative will be given sufficient time to read the ICF and to ask additional questions. After this explanation and before entry to the study, consent should be appropriately recorded by means of either the subject's or his/her legal representative's dated signature or the signature of an independent witness who certifies the subject's consent in writing. After having obtained the consent, a copy of the ICF must be given to the subject.
In case the subject or legally acceptable representative is unable to read, an impartial witness must attest the informed consent.
Subjects who are unable to comprehend the information provided or pediatric subjects can only be enrolled after consent of a legally acceptable representative.
The following information should be added to the electronic medical record (EMR):

- which version of the ICF was obtained
- who signed the ICF
- if sufficient time has been given to consider participation into the study
- which investigator obtained ICF with the date of signature
- if a copy was provided to the patient
- start and end of participation in the study

15.3. Approval of the study protocol

15.3.1. General

The protocol has been reviewed and approved by the Ethics Committee of the Ghent University (Hospital), designated as the central Ethics Committee, after consultation with the local Ethics Committees, and the Federal Agency for Medicine and Health Products (FAMHP). This study cannot start before both approvals have been obtained.

15.3.2. Protocol amendments

Any significant change or addition to the protocol can only be made in a written protocol amendment that must be approved by the Central Ethics Committee (and the FAMHP if applicable).
Only amendments that are intended to eliminate an apparent immediate safety threat to patients may be implemented immediately.
Notwithstanding the need for approval of formal protocol amendments, the investigators are expected to take any immediate action, required for the safety of any subject included in this study, even if this action represents a deviation from the protocol. These actions should always be notified to the sponsor.

15.4. Confidentiality and Data Protection

All study data will be handled in accordance with the law on General Data Protection Regulation (GDPR) and institutional rules
[Belgian law dated on 30 July 2018 and 22 Aug. 2002].

The collection and processing of personal data from subjects enrolled in this study will be limited to those data that are necessary to fulfill the objectives of the study. These data must be collected and processed with adequate precautions to ensure confidentiality and compliance with applicable data privacy protection laws and regulations.

Appropriate technical and organizational measures to protect the personal data against unauthorized disclosures or access, accidental or unlawful destruction, or accidental loss or alteration must be put in place. Sponsor and site personnel whose responsibilities require access to personal data agree to keep the identity of subjects confidential.
The informed consent obtained from the subject includes explicit consent for the processing of personal data and for the investigator/institution to allow direct access to his or her original medical records (source data/documents) for study-related monitoring, audit, Ethics Committee review and regulatory inspection. This consent also addresses the transfer of the data to other entities, if applicable.

Privacy and confidentiality of data generated in the future on stored samples will be protected by the same standards applicable to all other clinical data.
Stored samples will be pseudonymized throughout the sample storage and analysis process and will not be labeled with personal identifiers.

15.5. Liability and Insurance

The sponsor has taken a no fault insurance for this study, in accordance with the relevant legislation (article 29, Belgian Law of May 7, 2004).
Sponsor: Ghent University Hospital
Insurance Details: Allianz Global Corporate & Specialty; Uitbreidingstraat 86, 2600 Berchem; Tel: +32 33 04 16 00
Polis number: BEL000862

15.6. End of Study Notification

If all subjects have completed the study, a notification of the end of the study should be submitted to the (Central) Ethics Committee and FAMHP. This notification should be made within 90 days of the end of the clinical study. In case of early termination (definition in CT-1, 4.2), this is reduced to 15 days.

16. Publication policy

This study will be registered at EudraCT and ClinicalStudies.gov, and results information from this study will be submitted to ClinicalStudies.gov. In addition, every attempt will be made to publish results in peer-reviewed journals.

17. Reference List

1. Thompson BT, Chambers RC, Liu KD. Acute Respiratory Distress Syndrome. N Engl J Med 2017;377:1904-5.
2. Wu C, Chen X, Cai Y, et al. Risk Factors Associated With Acute Respiratory Distress Syndrome and Death in Patients With Coronavirus Disease 2019 Pneumonia in Wuhan, China. JAMA Intern Med 2020.
3. Wang D, Hu B, Hu C, et al. Clinical Characteristics of 138 Hospitalized Patients With 2019 Novel Coronavirus-Infected Pneumonia in Wuhan, China. JAMA 2020.
4. Ruan Q, Yang K, Wang W, Jiang L, Song J. Clinical predictors of mortality due to COVID-19 based on an analysis of data of 150 patients from Wuhan, China. Intensive Care Med 2020.
5. Huppert LA, Matthay MA, Ware LB. Pathogenesis of Acute Respiratory Distress Syndrome. Semin Respir Crit Care Med 2019;40:31-9.
6. Weinberg PF, Matthay MA, Webster RO, Roskos KV, Goldstein IM, Murray JF. Biologically active products of complement and acute lung injury in patients with the sepsis syndrome. Am Rev Respir Dis 1984;130:791-6.
7. Gralinski LE, Sheahan TP, Morrison TE, et al. Complement Activation Contributes to Severe Acute Respiratory Syndrome Coronavirus Pathogenesis. mBio 2018;9.
8. Mulvey JJ, Magro CM, Ma LX, Nuovo GJ, Baergen RN. Analysis of complement deposition and viral RNA in placentas of COVID-19 patients. Ann Diagn Pathol 2020;46:151529.
9. Magro C, Mulvey JJ, Berlin D, et al. Complement associated microvascular injury and thrombosis in the pathogenesis of severe COVID-19 infection: A report of five cases. Transl Res 2020.
10. Gao T. Highly pathogenic coronavirus N protein aggravates lung injury by MASP-2-mediated complement over-activation. MedRxiv 2020;https://doi.org/10.1101/2020.03.29.20041962.
11. Jiang Y, Zhao G, Song N, et al. Blockade of the C5a-C5aR axis alleviates lung damage in hDPP4-transgenic mice infected with MERS-CoV. Emerg Microbes Infect 2018;7:77.
12. Jiang Y, Li J, Teng Y, et al. Complement Receptor C5aR1 Inhibition Reduces Pyroptosis in hDPP4-Transgenic Mice Infected with MERS-CoV. Viruses 2019;11.
13. Mastaglio S, Ruggeri A, Risitano AM, et al. The first case of COVID-19 treated with the complement C3 inhibitor AMY-101. Clin Immunol 2020:108450.
14. Diurno F, Numis FG, Porta G, et al. Eculizumab treatment in patients with COVID-19: preliminary results from real life ASL Napoli 2 Nord experience. Eur Rev Med Pharmacol Sci 2020;24:4040-7.
