# Supplementary material for: Efficacy and safety of the investigational complement C5 inhibitor zilucoplan in patients hospitalized with COVID-19: an open-label randomized controlled trial
Source: Respir Res. 2022 Aug 9;23:202. doi: 10.1186/s12931-022-02126-2 (PMC9361275; doi:10.1186/s12931-022-02126-2)
Supplement: Supplementary file 2 — Additional file 2. Statistical analysis plan. [file 12931_2022_2126_MOESM2_ESM.pdf]

STATISTICAL ANALYSIS PLAN

Study: ZILU-COV

Product: Zilucoplan®

A prospective, randomized, open-label, interventional study to investigate the efficacy of complement C5 inhibition with Zilucoplan® in improving oxygenation and short- and long-term outcome of COVID-19 participants with acute hypoxic respiratory failure.

**SAP/Amendment Number**	**Date**
Final SAP	Approved 18DEC2020

Confidentiality Statement

Confidential

This document is the property of UCB and may not – in full or in part – be passed on, reproduced, published, or otherwise used without the express permission of UCB.

TABLE OF CONTENTS

LIST OF ABBREVIATIONS 5
1 INTRODUCTION 7
2 PROTOCOL SUMMARY 7
2.1 Study Objectives 8
2.1.1 Primary objective(s) 8
2.1.2 Secondary objective(s) 8
2.2 Study endpoint(s) 9
2.2.1 Efficacy endpoint(s) 13
2.2.1.1 Primary efficacy endpoint(s) 13
2.2.1.2 Secondary efficacy endpoint(s) 13
2.2.2 Pharmacodynamic endpoint(s) 19
2.2.3 Pharmacokinetic endpoint(s) 20
2.2.4 Safety endpoint(s) 20
2.2.4.1 Key safety endpoint(s) 20
2.2.4.2 Safety endpoint(s) of special interest 20
2.2.4.3 Other safety endpoint(s) 20
2.3 Study design and conduct 22
2.4 Determination of sample size 23
3 DATA ANALYSIS CONSIDERATIONS 24
3.1 General presentation of summaries and analyses 24
3.2 General study level definitions 25
3.2.1 Analysis time points 25
3.2.1.1 Relative day 25
3.2.1.2 Study periods 26
3.2.1.3 Nominal Days 26
3.2.2 Definition of Baseline values 27
3.2.2.1 General baseline 27
3.2.2.2 Alternate baseline 28
3.3 Protocol deviations 28
3.4 Treatment assignment and treatment groups 28
3.5 Analysis sets 28
3.5.1 Enrolled Set 29
3.5.2 Randomized Set 29
3.5.3 Safety Set 29
3.5.4 Full Analysis Set 29
3.5.5 Completer Analysis Set 29
3.5.6 Per Protocol Set 29

3.5.7 Pharmacokinetic Analysis Set 29
3.6 Centres 29
3.6.1 Centre pooling strategy 29
3.6.2 Change of Centres 30
3.7 Coding dictionaries 30
3.8 Changes to protocol-defined analyses 30
4 STATISTICAL/ANALYTICAL ISSUES 31
4.1 Adjustments for covariates 31
4.2 Handling of dropouts or missing data 31
4.2.1 Safety laboratory data 32
4.2.2 Dates and times 32
4.3 Handling of repeated and unscheduled measurements 32
4.4 Interim analyses and data monitoring 32
4.4.1 Interim 1 32
4.4.2 Interim 2 33
4.4.3 Safety Data Interim Look 33
4.5 Multiple comparisons/multiplicity 33
4.6 Use of an efficacy subset of participants 33
4.7 Examination of subgroups 34
5 STUDY POPULATION CHARACTERISTICS 34
5.1 Participant disposition 34
5.2 Protocol deviations 36
6 DEMOGRAPHICS AND OTHER BASELINE CHARACTERISTICS 37
6.1 Demographics 37
6.2 Other Baseline characteristics 37
6.3 Medical history and concomitant diseases 40
6.4 Prior and concomitant medications 40
7 MEASUREMENTS OF TREATMENT COMPLIANCE 41
8 EFFICACY ANALYSES 41
8.1 Statistical analysis of the primary efficacy endpoint(s) 42
8.1.1 Derivations of primary efficacy endpoint(s) 42
8.1.2 Primary analysis of the primary efficacy endpoint(s) 43
8.1.3 Sensitivity analyses of the primary efficacy endpoint(s) 44
8.1.3 Supplementary analyses of the primary efficacy endpoint(s).... **Error! Bookmark not defined.**
8.2 Statistical analysis of the secondary efficacy endpoint(s) 45
9 PHARMACOKINETICS AND PHARMACODYNAMICS 59
9.1 Pharmacokinetics 59

9.2 Pharmacodynamics 59
10 SAFETY ANALYSES 59
10.1 Extent of exposure 59
10.2 Adverse events 59
10.3 Clinical laboratory evaluations 61
10.4 Vital signs, physical findings, and other observations related to safety 61
10.4.1 Vital signs 61
10.4.2 Electrocardiograms (if applicable) 61
11 REFERENCES 62
STATISTICAL ANALYSIS PLAN SIGNATURE PAGE 63

LIST OF ABBREVIATIONS

AE	Adverse Event
AECC	American-European Consensus Conference
ARDS	Acute Respiratory Distress Syndrome
CI	Coordinating Investigator
COVID-19	Coronavirus induced disease-2019
CT	Clinical Trial Unit
DSMB	Data Safety Monitoring Board
DSUR	Development Safety Update Report
EC	Ethics Committee
ECG	Electrocardiogram
eCRF	electronic Case Report Form
EDC	Electronic Data Capture
EPD	Electronic Participant Dossier
FAMHP	Federal Agency for Medicines and Health Products
FiO2	Fraction of inspired oxygen
FPI	First Participant In
FVC	Forced vital capacity
GCP	Good Clinical Practice
GDPR	General Data Protection Regulation
gMG	generalized myasthenia gravis
GMP	Good Manufacturing Practice
HIRUZ	Health, Innovation and Research Institute UZ Ghent
IB	Investigator's Brochure
ICF	Informed Consent Form
ICH	International Council for Harmonisation
IMNM	Immune Mediated Necrotic Myopathy
IMP	Investigational Medicinal Product
IMPD	Investigational Medicinal Product Dossier
LVLS	Last Visit, Last Subject
PCWP	Pulmonary Capillary Wedge Pressure
PEEP	Positive End Expiratory Pressure
PI	Principal Investigator
PaO_2	Partial pressure of oxygen
PNH	Paroxysmal Nocturnal Hemoglobinuria
SC	Subcutaneous
SAE	Serious Adverse Event
SC	Subcutaneous
sHLH	secondary hemophagocytic lymphohistiocytosis
SmPC	Summary of Product Characteristics
SOP	Standard Operating Procedure

SUSAR	Suspected Unexpected Serious Adverse Reaction
TERENA	Trans-European Research and Education Networking Association
TLC	Total Lung Capacity
TLS	Transport Layer Security

1 INTRODUCTION

The purpose of this statistical analysis plan (SAP) is to provide all information that is necessary to perform the required statistical analysis of this study to support the final clinical study report (CSR). It also defines the summary tables, figures and listings (TFLs) to be generated (specific shells will be provided in a separate document) to be included in the report.

The SAP is based on the following study documents: Protocol version 3.0 dated 29May2020.

If a future protocol amendment necessitates a substantial change to the analysis of the study data, this SAP will be amended accordingly. In addition, if the methodology for the analysis of key study endpoints must be modified or updated prior to database lock, an SAP amendment will be required. If, after database lock, additional analyses are required to supplement the planned analyses described in this SAP, these changes will be described in the clinical study report together with the associated rationale.

The content of this SAP is compatible with the International Conference on Harmonization (ICH)/ Food and Drug Administration (FDA) E9 Guidance documents.

2 PROTOCOL SUMMARY

Details of the study design are described in the protocol and Section 5 of this SAP, but a brief outline is as follows.

The purpose of this phase 2a (proof of concept), multi-center,open-label 2:1 randomised (Experimental Arm: Control Arm) study is to investigate the efficacy and safety of Zilucoplan® in improving oxygenation and short- and long-term outcome of COVID-19 participants with early acute hypoxic respiratory failure (Acute hypoxemic respiratory failure is severe arterial hypoxemia that is refractory to supplemental oxygen) The participant population are participants with confirmed (see inclusion criteria) COVID-19 infection and with presence of hypoxic respiratory failure defined as O_2 saturation below 93% on minimal 2l/min O_2 therapy and/or PaO_2/Fi O_2 ratio below 350 mmHg. A sufficient number of participants will be enrolled in order to randomise 81 participants onto one of the 2 treatment arms in a 2:1 ratio. A 2:1 randomisation ratio was chosen to give the opportunity for more participants to potentially benefit from treatment with Zilucoplan and also it may be possible to include participant level data from another trial to augment the control arm.

Participants in the experimental arm (Arm A) will receive standard of care (SoC) + subcutaneous Zilucoplan® once daily and prophylactic antibiotics once daily (until 14 days after the last Zilucoplan® dose). Participants in the Control Arm (Arm B) will receive SOC+1 week of prophylactic antibiotics once daily (or until hospital discharge, whichever comes first).

The study consists of a screening day, 28-day primary assessment period, D28 (by phone call, unless still in hospital) and a final follow-up visit sometime between 12 to 22 weeks post-randomisation. For Arm A, the 28-day primary assessment period includes an in-participant treatment duration of up to 14 days (less if discharged from hospital before D15) of Standard of Care (SoC) + subcutaneous Zilucoplan® once daily + IV prophylactic antibiotics once daily, followed by 14 days of SoC +IV prophylactic antibiotics. It is required that the participant

should continue prophylactic antibiotics once daily for 14 days after the last dose of Zilucoplan®. If the participant is discharged before D28, daily prophylactic antibiotic treatment will be taken orally until 14 days after last dose of Zilucoplan®. For Arm B, the 28-day assessment period comprises SOC treatment up to 28 days post randomization or hospital discharge, when the D28 nominal day procedures. Arm B will only have 1 week of IV prophylactic antibiotics once daily, starting on D1. If hospital discharge occurs before D28 post-randomisation, both SOC and antibiotic treatment will cease and the D28 nominal day procedures will occur by telephone.

For both arms, whilst in hospital, assessments will continue on a daily basis as possible, until discharge or Day 28 is reached. If discharge is prior to D28, no daily assessments will be carried out – just the D28 telephone call.

The maximum time window for an eligible participant signing the informed consent document (and starting screening) and being randomised is 24 hours. It is assumed that first dose occurs very soon after randomisation.

An interim analysis (Interim 1) of the primary endpoints is planned after approximately half the planned number of participants have been dosed and had the opportunity to complete D15 assessments. The purpose of the interim analysis is to understand if there is already enough evidence to prompt further work on COVID-19 treatment with Zilucoplan®. There is currently no plan to stop the study based on interim results at this stage. Other endpoints may be summarised as the data allows. Safety data will also be reviewed and summarised as appropriate.

There will also be an interim analysis (Interim 2) when all participants have had the opportunity to complete D28 procedures (by phone call) to assess the primary endpoints, response rate, time to response, time to discharge and 28-day mortality, as appropriate. These analyses will enable key results to be understood as soon as possible, without compromising the study. In addition, safety will be summarised by incidence of TEAE and SAE by treatment arm. Graphs showing the time course for oxygen levels and 6-point ordinal score and Kaplan-Meier plots for time-to - event variables will be provided.

Final analyses will be carried out after all participants have either discontinued prematurely from or completed the study, by completing the follow-up visit.

2.1 Study Objectives

2.1.1 Primary objective(s)

The primary objective of this intervention is to study if Zilucoplan® plus standard of care improves the median and/or mean change in oxygenation between enrolment (baseline) and at predose day 6 and day 15 (or discharge, whichever comes first) compared to standard of care through assessment of the PaO2/FiO2 ratio and through measurement of the P(A-a)O2 gradient, which can easily be performed in the setting of clinical observation of participants admitted to the COVID-19 ward or ICU COVID-19 unit.

2.1.2 Secondary objective(s)

The secondary objectives are:

- to study if early intervention with Zilucoplan® is safe
- to study if early intervention with Zilucoplan® affects clinical outcome defined by duration of hospital stay, 6-point ordinal scale, time to defervescence, supplemental oxygen use SOFA score,
- to study if early intervention with Zilucoplan® affects progression to mechanical ventilation and/or ARDS and duration of ICU stay
- to study if early intervention with Zilucoplan® affects the rate of nosocomial infection
- to study if treatment with Zilucoplan® has a favourable effect on long term at follow up 12-22 weeks post-randomisation
- to study if treatment with Zilucoplan® affects all-cause mortality rate at day 28 and at 12-22 weeks post-randomisation

2.2 Study endpoint(s)

Endpoints listed in these subsections are definitions of the measures that are collected from each participant. How each of these are handled to assess the effectiveness of the intervention is described in the analysis sections.

Table 1.1 provides details of the schedule of events and when it is mandatory to take measurements of each endpoint. However, where possible, clinical and laboratory endpoints will be taken as frequently as possible; ideally daily up until hospital discharge.

Table 1.1

**Procedure**	**Screening (D0 or D1)**	**D1[a]**	**D2**	**D6**	**Hospital discharge or D15[b]**	**D28[c]**	**Follow-up[d]**
Informed consent	X						
Inclusion/exclusion criteria	X						
Randomisation		X					
Medical history & biometry	X						
6-minute walk test							X
Lung function							X
Physical examination[h,]	X	------------------------------Per SOC-----------------------------					
Vital signs[e,h]	X	----------------------------Daily until discharge-------------------------					**X**
Daily (S)AE inquiry		---					
Concomitant medication	X	----------------------------Daily until discharge----------------------				**X[c]**	X
ECG[h]	X		On clinical grounds				
Any chest CT scan	X[p]		On clinical grounds				
HRCT[q]							X
**Routine laboratory assessments** on clinical grounds, except:<br>- D0/1, D6, discharge/D15 and FU<br>- Procalcitonin 3x/week until D15	X[f,h]			X[g,h]	X[g]		X[g]
**Study blood sampling[h]**							
- 2 x 6 ml serum tubes		X		X	X		X
- 1 x 4 ml EDTA tube (DNA)		X					
- 2 x 10 ml EDTA tube (optional,selected sites)		X		X	X		X
- 1 x6 ml EDTA tube (complement)		X	X	X	X		
- 1 x 6 ml EDTA tube for PK (arm A only)		X	X	X	X		
- Arterial blood gas	X[h]			X[i.h]	X[i]		X
**Score assessments**							
- 6-point ordinal scale [j,h]	---Daily until discharge----------------------------------						X
- SOFA score [k,h]		X		X		X	
- HScore [l,h]		X					
- WHO performance scale [r]							X
IMP Zilucoplan® (arm A only)		------------------Daily SC until D14 or discharge-----------------					
Third generation cephalosporin (arm A only)[m]		------------------Daily IV until discharge--------------------------------------					

Procedure	Screening (D0 or D1)	D1[a]	D2	D6	Hospital discharge or D15[b]	D28[c]	Follow-up[d]
Third generation cephalosporin (arm B only)[n]		--------Daily IV until D7-------					
Oral ciprofloxacin (arm A only)[o]					-------------[o]		
Daily drug compliance enquiry		--				X[c]	

[a] Prior to first Zilucoplan® administration in arm A, and during the day in arm B

[b] Hospital discharge or D15, whichever comes first

[c] Enquiry by phone on D28 post-randomisation (or within next 3 days) for 6-point ordinal scale, (s)AE, supplemental oxygen (Y/N) and concomitant medication since discharge or D15

[d] Follow-up visit at 12-22 weeks post-randomisation

[e] Values between 7-10am includes T°C (actual and highest last 24h), Pulse Rate, Blood Pressure, Respiratory Rate, SpO2 by pulsoximetry, FiO2 and ventilator parameters (if participant admitted to ICU)

[f] Minimally includes Ferritin, LDH, haptoglobin, D-dimers, CRP, creatinine, fibrinogen, eosinophils, lymphocytes, haemoglobin, WBC count, platelets, bilirubin, ALT and AST, procalcitonin and bHCG (females) (for exclusion criteria, HScore and SOFA)

[g] Should minimally include Ferritin, LDH, haptoglobin, D-dimers, CRP, creatinine, fibrinogen, eosinophils, lymphocytes, haemoglobin, WBC count, platelets, bilirubin, ALT and AST (for SOFA)

[h] If arm A: Pre-dose Zilucoplan®

[i] Mandatory: participant in upright position, after minimal 3 minutes without supplemental oxygen. In case of inability to sit upright: same position is to be used for all measurements of PaO2. In ventilated participants or ECMO participants PaO2 can be taken from invasive arterial line and FiO2 taken directly from mechanical ventilation settings, document FiO2 at moment of ABG. For patients on ECMO the PaO2/FiO2 ratio and Aa-gradient cannot be calculated as in this case the FiO2 will be missing. Therefore, the last available value of PaO2/FiO2 ratio and Aa gradient prior to ECMO can be used at that timepoint.

[j] Defined as 1 = Death; 2 = Hospitalized, on invasive mechanical ventilation or ECMO; 3 = Hospitalized, on non-invasive ventilation or high flow oxygen devices; 4 = Hospitalized, requiring supplemental oxygen; 5 = Hospitalized, not requiring supplemental oxygen; 6 = Not hospitalized

[k] SOFA score (see https://www.mdcalc.com/sequential-organ-failure-assessment-sofa-score): requires PaO2, FiO2, platelet count, GCS, bilirubin, MAP and creatinine. To be evaluated between 6 and 10 am. Score to be calculated based on the worst values in the previous 24h at time of evaluation.

[l] HScore (see https://www.mdcalc.com/hscore-reactive-hemophagocytic-syndrome): requires T°C, haemoglobin, wbc count, platelets, ferritin, fibrinogen, triglycerides and AST (BM aspirate is not required) To be evaluated at time of randomization.

[m] Third generation cephalosporin replaces empiric amoxicillin/clavulanic acid to treat concomitant community acquired pneumonia. In any case that antibiotics need to be switched, *N. menigitidis* and *S. pneumoniae* need to be covered by the new antibiotic treatment (e.g. meropenem or piperacilin/tazobactam). Third generation cephalosporin is given until D28 or hospital discharge whichever comes first.

[n] Group B participants receive daily injection of 2g ceftriaxone IV (or appropriate alternative in case of allergies or on clinical indication) during one week or until hospital discharge, whichever comes first.

[o] Upon discharge before D28 post-randomization, participants will switch to daily oral ciprofloxacin (500 mg 1x/d) until at least 14 days after the last dose of Zilucoplan® (only in arm A).

[p] any form of chest CT scan within 48h before randomization (low dose, HRCT, or Angio CT scan) must document a presence of bilateral ground glass infiltrates or bilateral consolidation

[q] HRCT scoring: A subjective assessment of the overall extent of normal attenuation, reticular abnormalities, honeycombing and traction bronchiectasis will be performed. A reticular abnormality is defined as a collection of innumerable areas of small linear opacity. Honeycombing is defined as the presence of a cystic airspace measuring 3–10 mm in diameter, with 1- to 3-mm thick walls. Traction bronchiectasis is defined as irregular bronchial dilatation within the surrounding areas showing

parenchymal abnormalities. The morphological criteria on HRCT scans include bronchial dilatation with respect to the accompanying pulmonary artery, a lack of tapering of the bronchi and the identification of bronchi within 10 mm of the pleural surface. The HRCT findings will be graded on a scale of 1–4 based on the classification system: 1. normal attenuation; 2. reticular abnormality; 3. traction bronchiectasis; and 4. honeycombing. The presence of each of the above four HRCT findings will be assessed independently in three (upper, middle and lower) zones of each lung. The upper lung zone is defined as the area of the lung above the level of the tracheal carina, the lower lung zone is defined as the area of the lung below the level of the inferior pulmonary vein and the middle lung zone is defined as the area of the lung between the upper and lower zones. The extent of each HRCT finding will be determined by visually estimating the percentage (to the nearest 5%) of parenchymal involvement in each zone. The score for each zone will be calculated by multiplying the percentage of the area by the grading scale score. The six zone scores will be averaged to determine the total score for each participant. The highest score is 400 points and the lowest score is 100 points using this calculation method. The total score is the “HRCT fibrosis score”. (https://www.ncbi.nlm.nih.gov/pmc/articles/PMC3922654/pdf/1465-9921-15-10.pdf)

[r] WHO performance scale: 0. Able to carry out all normal activity without restrictions; 1. Restricted in physically strenuous activity but ambulatory and able to carry out light work; 2. Ambulatory and capable of all self-care but unable to carry out any work: up and more than 50% of waking hours; 3. Capable of only limited self-care: confined to bed or chair more than 50% if waking hours; 4. Completely disabled: cannot carry on any self-care: totally confined to bed or chair.

2.2.1 Efficacy endpoint(s)

2.2.1.1 Primary efficacy endpoint(s)

- Change from baseline in PaO_2/FiO_2 ratio to Day 6 and Day 15 (or at discharge, whichever comes first)
- Change from baseline in $P(A\text{-}a)O_2$ gradient to Day 6 and Day 15 (or at discharge, whichever comes first).
- Change from baseline in a/A pO_2 ratio to Day 6 and Day 15 (or at discharge, whichever comes first

These endpoints are derived from variables obtained from arterial blood gas sampling.

2.2.1.2 Secondary efficacy endpoint(s)

All time to event endpoints are measured from date of randomization. It is expected that there will be approximately 24 hours between randomization and dosing.

All endpoints associated with each secondary objective are listed together. The key endpoints are written in bold to be analysed/summarized at the second interim analysis when all participants have had the opportunity to complete Day 28 Procedures. Collected endpoints and their timing that may be used to derive the endpoints are listed in the third column. Alternative sources of the same information may be available and used.

There is overlap on some of the measurements across endpoints. Some may be taken once for each endpoint or taken once and used for more than one endpoint.

Details of the items in the 6-point ordinal score are provided below this table. Brief details of SOFA, are provided and further information can be obtained from the hyperlink.

**Secondary Objective**	**Endpoint**	**Measurement**
1) To study if early intervention with Zilucoplan® affects clinical outcome defined by duration of hospital stay, 6-point ordinal scale, time to defervescence, supplemental oxygen use, SOFA score	Incidents of participants reporting each severity rating in 6-point ordinal scale at D1, D6, D15 and D28<br><br>Change from baseline in 6-point ordinal scale to Day 6, Day 15 and Day 28 (by phone call) weeks post randomisation<br><br>**Time to at least a 2-point improvement or discharge sustained up to D28 on the 6-point ordinal scale during 28-day assessment period**<br><br>**Percentage of participants reporting at least a 2-point improvement sustained to D28 or discharge**<br><br>**AUEC/Time using the 6-point score recorded daily – up to D15, D28 and all data recorded**<br><br>**Percentage of participants who record a 2-point improvement from baseline or discharge at D6, D15 and D28**<br><br>**Percentage of participants not deteriorating according to the ordinal scale by 1 or 2 points on D6, D15 and D28**<br><br>6-point ordinal Scale at D6 and D15 and D28 in relation to D-Dimers and complement C5a levels at baseline	**6-point ordinal scale measured daily up to discharge, D28 and follow-up**<br><br>Lab values at baseline for D-Dimers and C5a
	Change from baseline in SOFA score (https://www.mdcalc.com/sequential-organ-failure-assessment-sofa-score) to Day 6, Day 15 (or at discharge, whichever comes first)	SOFA score measured at D1, D6 and D15

**Secondary Objective**	**Endpoint**	**Measurement**
1)continued:<br>To study if early intervention with Zilucoplan® affects clinical outcome defined by duration of hospital stay, 6-point ordinal scale, time to defervescence, supplemental oxygen use, SOFA score	Number of days with fever (defined as 37.1ºC or more) during 28-day assessment period<br>Time since randomization until absence of LAST fever (defined as 37. 1ºC or more) for more than 48 h without antipyretics (use last fever to account for possibility that participants have intermittent fever per this definition)	Temperature Vital Parameters<br>Antipyretic use in last 24 hours is collected on eCRF on every day. Use highest temperature recorded in last 24 hours, not the 7-10am temperature
	Duration of hospital stay	From DATES eCRF
	Duration of hospital stay of survivors	From DATES
	Number of days requiring supplemental oxygenation after randomisation to Day 28 (or discharge, whichever comes earlier)<br>Time since randomization until improvement in oxygenation (defined as independence from supplemental oxygen) during 28-day assessment period (or discharge, whichever comes earlier)	Number of days where Suppl O_2 = 'Y' from Vital parameters<br>Independence from supplementary oxygen means nor further 'Y' after a 'N'
	Number of days with hypoxia (defined as SpO2 <93% breathing room air or the dependence on supplemental oxygen after randomisation to Day 28 (or discharge, whichever comes earlier)	Use SpO2 (%) and Supple O2 (Y/N) from Vital parameters

**Secondary Objective**	**Endpoint**	**Measurement**
2) To study if early intervention with Zilucoplan® affects **progression** to mechanical ventilation and/or ARDS<br>And<br>Duration of ICU stay	Duration of ventilator free days after randomisation to Day 28 (or discharge, whichever comes earlier)<br>Duration of invasive mechanical (including ECMO) and non-invasive mechanical ventilation in ventilated participants	Use data from Vital parameters (include all days, where NIV, IV and ECMO='N')<br><br>Complement of above (exclude participants who are not ventilated i.e. do not include these with a duration of 0)
	Time since randomization until first use of high-flow oxygen devices or non-invasive mechanical ventilation or invasive mechanical ventilation or ECMO in non-ventilated participants (i.e. excluding participants who are ventilated within 24h prior to or after randomization) during 28-day assessment period	Use Vital Parameter CRF,
	Time since randomization to progression to ARDS	Randomisation date<br>Date of ARDS diagnosis
	Duration of ICU stay in participants that enrolled in trial that were on invasive or non-invasive mechanical ventilation or high-flow oxygen devices within 24h prior to or after randomization during 28-day assessment period<br>Duration of ICU stay in participants that enrolled in trial that were on invasive or non-invasive mechanical ventilation for less than 24h prior to or after randomization during 28-day assessment period	Use DATES and Vital Signs dataset

**Secondary Objective**	**Endpoint**	**Measurement**
3) To study if treatment with Zilucoplan® affects all-cause mortality rate at day 28 and at 12-22 weeks post-randomisation	**All-cause mortality rate at 28 days post randomisation (all treated participants)**<br><br>All-cause mortality rate at 28 days post randomisation (excluding participants that required invasive mechanical ventilation or ECMO, non-invasive mechanical ventilation or high-flow oxygen devices within 24 hours prior to or after randomisation)<br><br>All-cause mortality rate at 28 days post randomisation (excluding participants that required invasive mechanical ventilation or ECMO within 24 hours prior to or after randomisation)<br><br>All-cause mortality rate at 28 days post randomisation (excluding participants that required invasive mechanical ventilation or ECMO, non-invasive mechanical ventilation or high-flow oxygen devices within 24 hours prior to or after randomisation)<br><br>All-cause mortality rate at 28 days post randomisation (only including participants that required invasive mechanical ventilation or ECMO within 24 hours prior to or after randomisation)<br><br>All-cause mortality at follow up 12-22 weeks post-randomisation for all treated participants	**6-point ordinal scores, DATES and VITAL SIGNS datasets**

**Secondary Objective**	**Endpoint**	**Measurement**
4) To study if treatment with Zilucoplan® has a favourable effect on long term at follow up 12-22 weeks post-randomisation	Incidents of participants in each category of the 6 point ordinal scale at follow-up 12-22 weeks post randomisation	6-point ordinal score
	Incidents of lung fibrosis on chest high-resolution computed tomography (HRCT) scan at follow up 12-22 weeks post-randomisation<br><br>Incidents of lung function abnormalities at follow up 12-22 weeks post-randomisation	HRCT results<br><br>Lung function results – dataset should list all abnormalities in a consistent way across participants, including 'None'
	Results from the 6 minute walk test	
	Incidents of participants in each category of the WHO performance scale	WHO score
5) To study if early intervention with Zilucoplan® affects the rate of nosocomial infection	Incidents of nosocomial bacterial or invasive fungal infection during 28-day assessment period<br><br>Note: Participants with viral respiratory infection are at risk of secondary bacterial infections. As part of clinical routine care, sputum or BAL samples will be collected in participants suspected of secondary bacterial pneumonia and checked for presence of bacteria. Measurements of procalcitonin levels will be performed at least 3x/week until 14 days or hospital discharge	Data for bacteria will be presence/absence at each day tested.<br><br>Procalcitonin will be concentrations
6) Change from baseline in specific labs	Mean change in ferritin levels between D1 and D6 and between D1 and D15 (or discharge in each case)<br><br>Mean change in CRP levels between D1 and D6 and between D1 and D15 (or discharge in each case)	Laboratory values

Note: Please refer to Section 3.2.1.3 for details about assigning the correct nominal day in the event that participants are discharged from hospital due to clinical improvement prior to D28.

The 6-point ordinal score is collected daily and the 6 points are defined by:

- 1. Death
- 2. Hospitalized, on invasive mechanical ventilation or ECMO
- 3. Hospitalized, on non-invasive mechanical ventilation or high flow oxygen devices
- 4. Hospitalized, requiring supplemental oxygen
- 5. Hospitalized, not requiring supplemental oxygen
- 6. Not hospitalized

For Grade 3, 'high-flow oxygen' consists of a heated, humidified high-flow nasal cannula delivering up to 100% heated and humidified oxygen at a maximum flow of 60 L/minute via nasal prongs or cannula to a participant at rates of flow higher than traditional low-flow therapy. In high-flow oxygen, the fraction of inspired oxygen (FiO_2) can be titrated from 21% to 100% independent of the flow rate.

SOFA score – This is a measure of mortality risk of participants admitted into an ICU. The higher the total score, the greater the risk of death. The score can be mapped to a probability of death, but the analysis will use the raw total score data. (https://www.mdcalc.com/sequential-organ-failure-assessment-sofa-score)

Additional endpoints may be explored using analytical models and graphs to help inform interpretation of the results, as required upon sight of the data.

2.2.2 Pharmacodynamic endpoint(s)

Plasma and serum samples will be collected for summary and exploratory analysis as appropriate (e.g. descriptive statistics, compare change from baseline between the two treatment arms), may include but are not limited to:

- ADA – D1 and long-term follow-up from serum samples (Summary only to indicate presence or not)
- Local safety labs such as D-dimers, LDH, ferritin, CRP, fibrinogen, aPTT, PT
- Complement system assessment (EDTA tubes) – D1, D2, D6 and D15 (or at hospital discharge, if earlier)

The following may be collected but will be analysed at a later stage, and not as part of the main analyses for the study

- Cytokines and biomarkers (from selected centres) on D1, D6 and D15 (or at hospital discharge, if earlier)
  - Cytokines may include, but not limited to:
    - IL-1beta, IL-1RA, IL-2, IL-8, IL-6, TNFa, GM-CSF, G-CSF, IP-10, MCP-1, MIP-1a, IFNg, and IL-10
  - Biomarkers may include but not limited to
    - sRAGE, Angiopoietin-2)

- DNA for pharmacogenomic research (Paxgene tube, D1 only)

2.2.3 Pharmacokinetic endpoint(s)

Blood samples will be taken from participants in Arm A only on D1, D2, D6 and day 15/discharge– all pre-dose.

2.2.4 Safety endpoint(s)

2.2.4.1 Key safety endpoint(s)

The key safety endpoints of this study are the incidence/number of adverse events (AE), serious adverse events (SAE) and the severity of AEs from first dose of study treatment on D1 until D28. Each AE will be categorized as a Treatment Emergent Adverse Event (TEAE) or not. All cases will be assessed by the reporting medically qualified professional or Sponsor as to having a reasonable suspected causal relationship to study treatment or not and categorized on the eCRF as

1. Not related
2. Unlikely
3. Possible
4. Probable
5. Definitely

All AE/SAEs that fall into the last 3 categories are deemed as related to study treatment and will be classified as an Adverse Reaction (AR) or Serious Adverse Reaction (SAR) respectively.

Each SAEs will also assessed as to whether it was an unexpected SAR (Suspected Unexpected Serious Adverse Event, SUSAR)

2.2.4.2 Safety endpoint(s) of special interest

Endpoints associated with nosocomial infection:

- Number of participants with at least one occurrence of nosocomial bacterial or invasive fungal infection for 28 days (phone call) after enrolment in trial

Participants with viral respiratory infection are at risk of secondary bacterial infections. As part of routine clinical care, sputum or BAL samples will be collected in participants suspected of secondary bacterial pneumonia, and checked for the presence of bacteria. Measurements of procalcitonin levels will be performed at least three times per week until day 14 or hospital discharge

2.2.4.3 Other safety endpoint(s)

- ECG – at screening and on clinical grounds
- CT scan – at screening and on clinical grounds
- Routine laboratory assessments – on clinical grounds but also on Screening, D6, D15 (or at discharge whichever is earlier) and long-term follow-up
    - At a minimum the following will be collected at Screening:

    - Ferritin, LDH, haptoglobin, D-dimers, CRP, creatinine, tryglycerides, fibrinogrn, eosinophils, lymphocytes, haemoglobin,WBC count, platelets, bilirubin, ALT and AST
    - bHCG for females
  - At a minimum the following will be collected at on D6, D15 (or at discharge whichever is earlier) and long term follow-up:
    - Ferritin, LDH, haptoglobin, D-dimers, CRP, creatinine, tryglycerides, fibrinogrn, eosinophils, lymphocytes, haemoglobin,WBC count, platelets, bilirubin, ALT, AST
  - Procalcitonin – Screening and 3x week for 14 days or hospital discharge
- Physical examination – at Screening, as required by standard of care (SOC) treatment and long term follow-up
  - including but not limited to
    - cyanosis, crepitation and rales, heart murmurs and peripheral edema
- Vital Signs – at Screening, daily until discharge andlong term follow-up
  - Temperature (actual 8-10 am and highest in last 24 hours)
  - Pulse rate
  - Blood pressure
  - Respiratory rate
  - SpO_2 by pulse oximetry
  - Supplemental Oxygen (Y/N) and Ventilation Method (plus flow rate when appropriate)
  - FiO_2(%) – for each method used
    - If participant is admitted to ICU the following invasive mechanical ventilation monitoring data
      - Arterial blood pressure
      - PCWP
      - Continuous O_2 saturation
      - Continuous ECG
      - Ventilatory parameters (tidal volume, FiO_2, PEEP pressure, peak pressure, minute ventilation)
- 6 minute walk test – long term follow-up only
- Lung Function – long term follow-up only
  - FVC, FEV1, FRC, RV, DLCO
- HRCT Scan – at long term follow-up

- HScore - taken at screening only. This is an algorithm that results in an integer value ranging from 0 to 337. Different scores are grouped together and each mapped onto a range of probabilities of hemophagocytic syndrome. In addition, an HScore >169 are 93% sensitive and 86% specific for HLH.

2.3 Study design and conduct

This investigator-initiated trial is a phase 2 academic, prospective, 2:1 randomized, open-label, multi-center interventional study designed to investigate the efficacy of subcutaneous Zilucoplan® in improving oxygenation and short- and long-term outcome of COVID-19 participants with acute hypoxic respiratory failure.

Several academic and non-academic centers in Belgium will participate in this trial. Study coordination, monitoring and data management will be performed under the responsibility of the Health Innovation and Research Institute UZ Gent (HIRUZ) Clinical Trial Unit and Data Management Unit.

81 participants with confirmed COVID19 with acute hypoxic respiratory failure (saturation below 93% on minimal 2 L/min supplemental O2 or PaO2/FiO2 <350) will be randomised to receive up to 14 days of SC Zilucoplan® on top of standard of care (active Arm A), or to receive standard of care treatment (control Arm B). Randomization will be done at a 2:1 ratio active: control group. In the active Arm A and in the control Arm B, participants will additionally receive daily antibiotics (daily 3rd generation cephalosporin IV while in hospital, followed by oral ciprofloxacin while discharged) as primary prophylaxis against meningococcal disease until 14 days after the last dose of Zilucoplan®. Control Arm B will receive standard of care and prophylactic antibiotics (3rd generation cephalosporin IV) for only 1 week (or until hospital discharge whichever comes first), to control for the effects of antibiotics on the clinical course of COVID-19.

A schematic of the study design is provided in Figure 1.

To measure the effectiveness of Zilucoplan® on restoring lung homeostasis, the primary endpoints of this intervention is measuring change in oxygenation comparing change from baseline values (pre-treatment) to values pre-dose day 6 (or discharge whichever comes first) and to values at day 15 (or discharge whichever comes first) post-randomization between Arm A and Arm B.

The study is estimated to terminate 52 weeks after the first participant is dosed, including last clinical follow up visits.

Figure 1 Description of Study Design

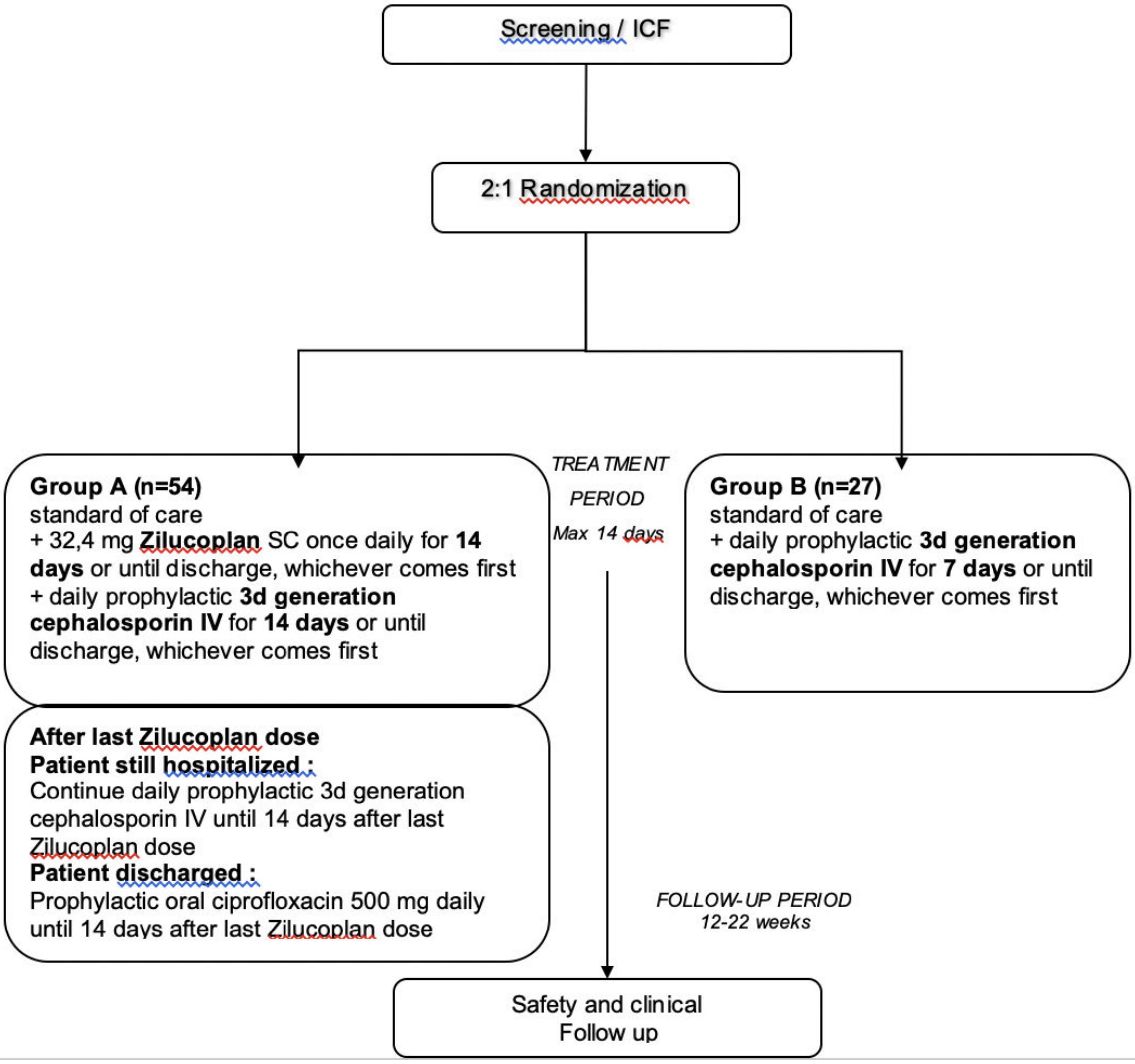

2.4 Determination of sample size

The outcome(s) on which the sample size calculation is based upon, is the primary endpoint measurement of oxygenation, defined as ratio of PaO2/FiO2.

The target difference is the difference from baseline measured at the primary endpoint (at day 6 and day 15) between the control and the treated group. Given a sample size of 81 participants, 54 on Zilucoplan® and standard of care and 27 on standard of care there would be >85% power to show a significant difference from standard of care at the 2 sided 5% level if the true underlying treatment difference was an 80 point difference (25% of 320, being the mean at hospital admission) in the PaO2/FiO2 ratio. This assumes a standard deviation of 105 and a dropout rate of less than 10%.

3 DATA ANALYSIS CONSIDERATIONS

3.1 General presentation of summaries and analyses

Statistical analysis and generation of tables, figures, participant data listings, and statistical output will be performed using SAS Version 9.1 or higher.

Descriptive statistics will be displayed to provide an overview of the study results. For categorical parameters, the number and percentage of participants in each category will be presented. The denominator for percentages will be based on the number of participants appropriate for the purpose of analysis. Unless otherwise noted, all percentages will be displayed to 1 decimal place. No percentage will be displayed for zero counts, and no decimal will be presented when the percentage is 100%. For continuous parameters, descriptive statistics will include number of participants with available measurements (n), mean, standard deviation (SD), median, minimum, and maximum.

Decimal places for descriptive statistics will always apply the following rules:

- “n” will be an integer.
- Mean, SD, and median will use 1 additional decimal place compared to the original data.
- Coefficient of variance (CV[%]) will be presented with 1 decimal place.
- Minimum and maximum will have the same number of decimal places as the original value.

Statistical tests of the primary efficacy endpoints will be presented as 2-sided p-values rounded to 4 decimal places. P-values less than 0.0001 will be presented as “<0.0001” and p-values greater than 0.9999 will be presented as “>0.9999.”. Appropriate statistical models will be fit to the various data collected for the secondary objectives, as described in their respective sub-sections in Section 8 for the purposes of estimation. No p-values will be provided for the analysis of endpoints associated with the secondary objectives, rather 95% confidence limits of the results of various comparisons of interest will be provided.

Data listings containing documented and calculated data (eg, change from Baseline) may be generated if it supports the interpretation of any analysis. Unless otherwise stated, listings will be sorted by treatment arm, participant number within arm, parameter (if applicable), nominal day and relative day respective to first dose and relative day with respect to last dose for any data collected after discontinuation from treatment. All listings will include all measurements which will appear in chronological order together with the scheduled nominal days. In all listings dates will be presented in the format ‘YYYY-MMM-DD’ and times will be presented as ‘hh:mm’ or hh:mm:ss’ as needed.

A listing of reasons for screen fail will be provided for participants who fail screening. A listing of reasons for not being dosed will be provided for participants who were randomized but not dosed. will also be provided.

Appropriate descriptive statistics will be provided in summary tables by treatment and Nominal Day for all endpoints, collected or derived. A total column will always be presented.

Figures will be generated to visualise all the raw data (not just those that fall on the nominal days) to aid interpretation of the results as indicated in their respective sub-sections in Section 8.

Treatment labels for TFLs

Full treatment regimen	Summary tables label	Listings label
Group A: Standard of Care + Zilucoplan 32.4mg i.v. + prophylactic antibiotics	Zilucoplan 32.4mg i.v. + Standard of Care	Group A: Zilucoplan 32.4mg i.v. + Standard of Care + prophylactic antibiotics
Group B: Standard of Care + prophylactic antibiotics	Standard of Care	Group B: Standard of Care + prophylactic antibiotics
Notes:	Standard of Care maybe abbreviated to ‘SoC’ if so add Footnote: SoC=Standard of Care	Standard of Care maybe abbreviated to ‘SoC’ if so add Footnote: SoC=Standard of Care<br>If the label is still too long for some listings the ‘Group A/B’ may be dropped’.

3.2 General study level definitions

3.2.1 Analysis time points

3.2.1.1 Relative day

The relative day of an event or assessment will be derived using the date/time of first dose of study treatment as reference. This is the date/time of first dose of Zilucoplan® for participants randomised to Arm A and first dose of IV antibiotic for participants randomised to Arm B.

For the purposes of reporting, there is no relative Day 0. Note that the protocol refers to ‘Screening (D0 or D1)’. This is to indicate that it is expected that screening will only take 24 hours and ends at first dose on D1. However, for some participants this is two days (D0 and D1) and for others it is only 1 day (D1) Hence the SAP/TFLs will state ’Day -1’ if the day before dosing and ‘Day 1’ if the day of dosing to ensure differentiation of the days.

Relative days for an event or assessment occurring before the date/time of first dose are calculated as follows and preceded by ‘-‘:

Relative Day = Event/Assessment Date/Time – Date/Time of First Dose

If Time has not been collected for the event/assessment, checks must be made to ensure that any Event/Assessment made on the same day, but PRIOR to first dose, is indicated as this will form part of the calculation to identify baseline values.

The relative day for an event or assessment occurring on or after the first dose to the date of the last dose of study treatment will be calculated as follows:

Relative Day = (Event/Assessment Date – Date of First Dose) + 1

For events or assessments occurring after the date of the last dose, relative day will be prefixed with '+' in the data listings and will be calculated as follows:

Relative Day = Event/Assessment Date – Date of Last Dose

Relative day will not be calculated in cases where dates are partial and should be presented as "--"in the relevant data listing.

3.2.1.2 Study periods

The following are defined as study periods:

Screening	Prior to the time of first dose of Zilucoplan® (Arm A) or IV antibiotic (Arm B)
Primary Assessment Period	For Arm A, from the date/time of first dose of Zilucoplan® to the end date* of the Primary Assessment Period<br>For Arm B, from the date/time of first dose of IV antibiotic to the end date* of the Primary Assessment Period
Planned Active Treatment Duration	For both arms the planned active treatment duration is from D1 up to D14 or hospital discharge, whichever is earlier.
D28 (minimal assessments carried out (6-point ordinal scale, (S)AE supplemental oxygen need (Y/N), concomitant medication)	28 days post randomization (by phone call, unless still hospitalised)
Follow-up	12-22 weeks post randomisation

* The end date of the primary assessment period for each participant will be 28 days post-randomisation

The end of the study is defined by the date of the Follow-up visit for the last participant to complete their Follow-up visit. In the event that participants are lost to follow-up, the study is not deemed to have ended until the last participant dosed has had the opportunity to complete their follow-up visit as scheduled.

3.2.1.3 Nominal Days

The pre-specified protocol nominal days are labelled as follows:

- Day -1
- D1
- D2

- D6
- D15
- D28
- Follow-up

The study protocol specifies that clinical and laboratory parameters be collected as frequently as possible, which would be daily. A window of +/- 1 relative day for each specified nominal day after D2 (+ 1 day for D2) will be allowed if a measurement was not collected on the specified relative day. The window for D28 is +/- 3 days. If a large proportion of data fall outside this window, then increasing the window to +/-2 days for nominal D6, nominal D15 and D28 will be considered.

An additional indicator variable will be derived, DISCHARGE DAY (Y/N), which will identify the relative day that the participants was discharged from hospital. If discharge is on or earlier than relative day 6, between relative day 7 and relative day 15 inclusive or between relative day 16 and relative day 28 inclusive, an additional row mapping the results of the discharge visit to nominal D6, or nominal D15 and nominal D28 respectively. For the primary endpoints a LOCF approach will be utilized for the participants who discharge i.e. if a participant discharges at Day 4 then that oxygenation measure will be mapped to Day 6, Day 15 and Day 28. The approach may be extended to secondary parameters. Data from participants who leave hospital for reasons other than clinical improvement will not be mapped to the nominal days as described above. Participants who die whilst in hospital are not considered as ‘discharged’. Separate indicator variables will be derived to indicate whether a participant has died on a specific relative day and if treatment was discontinued due to an adverse event.

The Nominal Day variable with the above labels will be derived from the relative days and allowable window for all collected endpoints. Since the ideal is to take measurements as frequently as possible, some measurements of some endpoints will not have a Nominal Day associated with them. When these values (without an associated Nominal Day) are to be summarized, graphed or analysed in statistical models, Relative Day will be used as either a continuous variable or categorical as defined in the respective sections. Nominal Day will only ever be used as a categorical variable.

3.2.2 Definition of Baseline values

3.2.2.1 General baseline

In general, Baseline values for efficacy and safety endpoints will be determined from the last non-missing data value collected prior to the first dose of Zilucoplan®, unless otherwise noted for a specific type of data in Arm A. For Arm B, baseline values will be the first value taken on the designated Day 1 of study medication, defined as the day the participant first receives IV antibiotic. For endpoints which measurements are not taken on Day 1, Baseline will be the value taken at Screening.

Note that for the Nominal Day variable, identified baseline values (whether they were actually taken at Day -1/Screening or pre-dose D1) will be mapped to D1. If this value was taken at Day -

1 (Screening), there will be a missing value associated with the Day -1(Screening) level in order not to duplicate results.

3.2.2.2 Alternate baseline

Due to the severity of disease in hospitals and the pandemic conditions some participants may not have a baseline recorded prior to their first dose. In the event there is no measurement at all prior to dosing for the key parameters then a measurement up to 2 hours postdose (this time frame may change based on PK/PD data) may be included as baseline for that participant. This baseline will be referred to as the ‘alternate baseline’ and will be used in sensitivity analyses.

3.3 Protocol deviations

Important protocol deviations are deviations from the protocol which potentially could have a meaningful impact on study conduct or on the primary efficacy (effectiveness) or safety for an individual participant. The criteria for identifying important protocol deviations may be defined within the appropriate protocol-specific document. Important protocol deviations will be reviewed as part of the ongoing data cleaning process and data evaluation documented in a protocol deviation log. All important deviations will be identified and documented prior to final database lock to confirm exclusion from analysis sets.

Note: whilst protocol deviations maybe listed for Interim1 it is unlikely sufficient determination on the effect of the deviations will have been reviewed and hence no one will be excluded from the PPS (section 3.5.6) for Interim1.

3.4 Treatment assignment and treatment groups

Participants will be randomized onto one of the two treatment arms (Arm A:Zilucoplan®+SOC, Arm B: SOC only) in a 2:1 ratio. The 2:1 randomisation will be carried out using REDCap (electronic IWRS system) by centre. No other stratification factors are planned.

3.5 Analysis sets

All safety analyses will be carried out on the Safety Set. All efficacy analyses will be carried out on the Full Analysis Set. They may be repeated on the Randomised and Per Protocol Set if these are generated. PK analyses will be carried out on the Pharmacokinetic Analysis Set.

It is expected that participants will receive treatment as randomized. However, if it is determined that participants received treatment other than what they were randomized to, then all analysis sets participants will be allocated to the actual treatment they received (SS-as treated, FAS – as treated, RS – as treated, PPS, PKS – as treated).

It is always appropriate to assess safety and PK based on actual treatment received. Since this is a PoC study, it is appropriate that treatment assignment for the FAS will be also be actual treatment received.

3.5.1 Enrolled Set

The Enrolled Set (ES) will consist of all participants who have given informed and will consist of all screened participants, including screen failures.

3.5.2 Randomized Set

The Randomized Set (RS) will consist of all participants randomized into the study. This will only be created if any participant was not dosed after randomization.

3.5.3 Safety Set

The Safety Set (SS) will consist of all participants who have received at least 1 dose of Zilucoplan® on Arm A and all participants who have had at least one dose of IV prophylactic antibiotic on Arm B.

3.5.4 Full Analysis Set

The Full Analysis Set will consist of all participants who have received at least 1 dose of Zilucoplan® on Arm A and who have had at least one dose of IV prophylactic antibody on Arm B. This is clearly the same as the SS but the FAS will be referred to when reporting results for efficacy endpoints.

3.5.5 Completer Analysis Set

The completer analysis set comprises participants who have received treatments per protocol (including patients who are discharged due to clinical improvement prior to completing the specified maximum number of days of treatment) and have complete data available up to D28 or up to date of death, whichever is sooner (Section 5.1)

3.5.6 Per Protocol Set

The Per Protocol Set (PPS) will consist of participants in the FAS without any major protocol deviations that may influence the validity of the data. If there are no major protocol deviations, this analysis set will not be created. This set will not be used for Interim1 as no protocol deviation meeting will have taken place.

3.5.7 Pharmacokinetic Analysis Set

The Pharmacokinetic Set (PKS) will be all participants in Arm A in SS who have at least one evaluable PK concentration.

3.6 Centres

3.6.1 Centre pooling strategy

All by participant listings will include the various centers. Summary statistics of all endpoints will be provided by center and treatment, as well as by treatment. The data from all centers will be pooled for both safety and efficacy summaries. Statistical models may include center as a covariate (center is included as a stratifying factor in the randomization). Centre by treatment interaction may be included if appropriate.

3.6.2 Change of Centres

Due to the ongoing pandemic participants may be required to move hospitals. No IMP will move with the participants and hence dosing will stop. Prophylactic antibiotic use will continue where appropriate.

If participants are moved to a hospital within the study then safety (conmeds, AEs, labs and temperature) and key efficacy data (primary oxygenation endpoint, ordinal scale, respiration data and mortality) will be prioritised.

If participants are moved to a hospital outside the conduct of the study then only key safety data (conmeds, AEs, SAEs) will be collected.

Centre changes will be captured and reported as well as day of transfer. Data collected for participants after transfer to another hospital may not be included in any formal analyses, but will be listed.

3.7 Coding dictionaries

Adverse events and medical history will be coded using CTCAE V5.0 and the latest available version of the Medical Dictionary for Regulatory Activities (MedDRA®) (Version v 22.0 or higher).

Medications may be coded using the World Health Organization Drug Reference List (WHO-DRL).

Medical procedures will not be coded.

3.8 Changes to protocol-defined analyses

The analysis of the primary endpoint described in the protocol omitted to add that baseline*day would be included as a fixed effect in the Mixed Model Repeated Measures (MMRM) analysis. This is needed in order to account for the fact that values from days closer to baseline are likely to be more correlated than values at days further apart from baseline. This is important if we include D28 and long-term follow-up in some of our analyses.

The primary analyses will be using Nominal Day as the categorical time point, and not strict relative day. This is because whilst the ideal is to take measurements as frequently as possible, the protocol only specifies specific days, which are the primary days for comparing between treatment arms. Therefore, windowing has been incorporated into the derivation of Nominal Day to ensure data that may not have been collected on the specific day of interest, but are within the window, are used. Sensitivity analyses using Relative Day (continuous and/or categorical) will be carried out as described in the relevant sections.

An additional variable will be created to indicate day of discharge and where needed, the results on the day of discharge will be mapped to the appropriate nominal day (Section 3.2.1.3).. This is to match with the sentence in Section 7.1.2 where it describes how to treat data if the day of discharge is prior to D15.

The SAP will not be amended for any changes to planned analyses or any additional analyses that are made after database lock

4 STATISTICAL/ANALYTICAL ISSUES

4.1 Adjustments for covariates

Covariates such as sex, age, BMI (measure of obesity) and race may also be included in the models if by the time of analysis these have been identified as key differentiators in terms of COVID-19 recovery.

Weight may also be used (independently of BMI) as a covariate to explore the impact of weight on results as the usual dosing regimen for Zilucoplan® is to dose by weight.

In addition, the impact on the results of disease severity at baseline may be explored using the 4 middle categories of the 6 point ordinal scale to define disease severity at baseline as a covariate

Where indicated, some secondary endpoints will explore the impact of baseline laboratory values on efficacy endpoints eg Baseline D-Dimers and C5a values.

The addition of the interaction of any baseline covariate with treatment may be explored to identify any evidence to suggest a differential impact on results.

Prior to fitting any model, all data will be explored graphically.

4.2 Handling of dropouts or missing data

The SOE indicates some endpoints are listed as only being collected on the specified days where comparisons between treatments are required, but all clinical and laboratory endpoints will be collected as frequently as possible, preferably daily, whilst in hospital. The primary assessment period is for 28 days post randomization, but some endpoints cannot be collected once a participant has been discharged from hospital e.g. vital signs (including the primary endpoints) and laboratory endpoints.

See Section 5.1 for definitions for Completed Treatment and Completed Study and their complements.

Missing values from participants take 4 forms:

- Missing due to death (no longer ‘at risk’ of recovery)
- Missing due to hospital discharge prior to end of primary assessment period (on any day up to D28)
- Missing values from the ideal of having daily collection of laboratory and clinical endpoints whilst participants are in hospital
- Missing values due to premature withdrawal from study

How missing data are to be handled is described in the analysis sections for each endpoint.

Participants who discontinue *study treatment* for reasons other than death or hospital discharge (for example, withdraw consent or adverse event, may still continue on study and will have measurements taken, so data from timepoints following discontinuation from *study treatment* will not be missing. These participants/data may be considered differently as part of a sensitivity analysis as they did not receive treatment per protocol.

4.2.1 Safety laboratory data

BLQ values should be set to half the LLOQ and any values above the limit of quantification (ALQ) will be assigned as the value of upper limit of quantification.

4.2.2 Dates and times

A description of how these are to be handled is in Section 13.1.

4.3 Handling of repeated and unscheduled measurements

No measurements are considered as unscheduled as the ideal is to take all clinical and laboratory measurements as frequently as possible.

Repeated measurements are defined as more than 1 measurement at the same timepoint. For example, the same ECG parameters assessed twice due to issues with the first assessment. The use of either the original or repeated measurement performed in any summary or analysis will depend on the reason for repeating the measurement. If repeated measurements are used in any analysis or summary, it will be clearly stated and the reason articulated.

Since the ideal is to take as many measurements as possible, it is possible that more than one measurement will be taken at different times on the same day but is not really considered a repeated measurement under the usual definition. In these cases, the first value taken on any day will be used in any analyses as these should hopefully be taken at the same time on each day and this should avoid any potential bias caused by diurnal variation.

Any listings and graphs of these data over time will include the repeated and/or unscheduled assessments. Sensitivity analyses may be performed using the original and repeated measurements. When more than 1 assessment is available for a specific study moment, these assessments are organized as first the original assessment, assigned to the nominal visit, and then the repeated assessment(s), assigned to unscheduled visits. The date and time of measurement are used to sort the data

4.4 Interim analyses and data monitoring

4.4.1 Interim 1

It is assumed that all data for the primary endpoints will not have been cleaned for this interim analysis.

The analyses of the primary endpoints will be as described in Section 8.1. In addition, the results of the secondary endpoints associated with a 2-point improvement in the 6-point ordinal scale may be analysed, if upon data exploration, formal comparison is meaningful – the percentage of participants who have achieved a sustained (to D15) 2-point improvement or discharge will be compared between treatments and the time to achieving the sustained 2 point improvement or discharge will be estimated. See Section 8.2.1 for a description of the analysis methods.

Safety data will also be reviewed (including adverse events) and presented as described in Sections 10.2 and 10.3 respectively. Mortality will be listed and/or summarized by nominal timepoint and treatment depending on if there is sufficient data to warrant a treatment summary.

There is no plan to stop the study based on results at this stage and no adjustment to control the overall Type 1 error at the final analysis is needed.

4.4.2 Interim 2

Interim 2 analyses will be carried out when all participants have had the opportunity to complete D28 procedures (in clinic or by a phone call if previously discharged) to assess 28 day mortality endpoints and endpoints associated with a 2 point improvement or discharge in the 6-point ordinal scale.

If all data from every participant for the primary objective and 6-point ordinal scale is available *and clean at this time*, this may form the final analysis for the primary objective and key secondary endpoints and be used to guide further research. i.e. there would be no need to wait for the final analysis planned to occur 12-22 weeks post randomization

The analyses of the primary endpoints, 28 day mortality and endpoints associated with a 2 point improvement in the 6-point ordinal score or discharge will be as described in Section 8.1 and Section 8.2.1 respectively.

Safety data will also be reviewed (Adverse events and clinical laboratory parameters) and presented as described in Sections 10.2 and 10.3 respectively.

4.4.3 Safety Data Interim Look

In addition to these interim analyses a Data Safety Monitoring Board is likely to happen after 10 participants have been dosed in Arm A. Additional DSMBs may occur as indicated. UzGhent will manage the DSMB (including the charter) however, the TFLs will be produced by UCB when the 10th active participant is entered in the database with data up to Day 15. The protocol does not state the timeframe of data required, however, it is judged that 15 days of data from 10 active participants will be sufficient to assess safety. AEs, Conmeds, laboratory values, vital signs, exposure will be investigated in Spotfire visualisations and summarized in TFLs if there are sufficient data. To put the safety data in context oxygenation levels, clinical scores, medical history and demography may be considered.

4.5 Multiple comparisons/multiplicity

Three endpoints are listed that are associated with the primary objective. There are no plans to control for multiplicity in the primary objectives as the endpoints are different parameterizations of the same underlying endpoint measured on separate days. This means there is effectively only one primary endpoint, PaO_2/FiO_2 ratio which was used to calculate sample size. The comparison of the differences from baseline to two days (D6/Early Discharge and D15/Early discharge) are of specific interest.

There are no plans to control for multiplicity in the secondary objectives as these hypotheses are considered exploratory. Therefore, any findings will need to be confirmed in subsequent studies.

4.6 Use of an efficacy subset of participants

Not applicable.

4.7 Examination of subgroups

Not applicable.

5 STUDY POPULATION CHARACTERISTICS

5.1 Participant disposition

Participants are considered **screenfails** if they were deemed not eligible for randomisation.

Since this is initially an in participant study the Nominal Days are not considered a scheduled protocol visit.

A participant who dies at any time during the study is considered as having **completed the study and primary assessment period.** If they discontinued early from planned active treatment duration (i.e. not including hospital discharge), the participant is considered as having **completed the study and primary assessment period, but not completed planned active treatment duration.**

In addition, a participant is considered to have **completed the study** if they have completed D28 and the follow-up visit. Participants can be discharged from hospital at any time, and as long as they have completed D28 and the follow-up visit, they have **completed the study**.

Participants have **completed primary assessment period** if they have:

- Arm A: completed up to 14 days of treatment with study drug followed by 14 days of either IV or oral prophylactic antibiotic treatment (as an in-participant or after discharge from hospital) AND completed D28 procedures
- Arm B: Completed up to 14 days of treatment with study drug (SOC+ 1 week of prophylactic antibiotic treatment) AND completed D28 procedures

Participants have **not completed primary assessment period**:

- are discontinued treatment prior to end of planned active treatment duration due to an adverse event - they may still complete D28 and/or follow-up visits
- discontinue treatment prior to end of planned active treatment duration as they meet an exclusion criterion (either newly developed or not previously recognized) that precludes further study treatment participation - they still may complete D28 and/or follow-up visits
- withdraw consent from being treated prior to end of planned active treatment duration – they may agree to complete D28 and/or follow-up visits
- Complete the treatment as planned but do not complete D28 procedures

Participants have prematurely **withdrawn from the study** if they:

- are lost to follow-up, after hospital discharge (i.e. do not complete D28 and follow-up visits or do not complete follow-up visit).
- Withdraw consent from being treated prior to end of planned active treatment duration and from D28 and follow-up visits

- Withdraw consent for D28 and follow-up visit procedures visit after completing planned active treatment duration

A listing of participants who failed screening at the beginning of the study will be presented for the ES, providing dates of informed consent and primary reason for screenfail.

In addition, the disposition of participants screened and treated will be summarized by site. This table may include, for each site, the site number, principal investigator name, the dates of the first participant in and the last participant out, the number of participants screened (ES), randomized (RS), treated (SS) and included in the FAS, PPS and PKS (if RS and PPS generated).

Disposition (completed study, completed primary assessment period, completed planned treatment duration or prematurely withdrawn from study and subsets) in each analysis set will be summarized by treatment group, and overall.

The number of participants who enrolled (overall only) and the number and percentage of screenfails (overall only) will be provided.

The following will be provided for the FAS, by treatment arm and overall.

- The number of participants randomized (used as basis for following percentages)
- The number of participants who started treatment
    - The number and percentage of participants who **complete** the study
        - Of these,
            - The number and percentage of participants who were discharged prior to D15
            - The number and percentage who died and how many of these died prior to D15
    - The number and percentage of participants who **complete the primary assessment period**
        - Of these,
            - The number and percentage of participants who were discharged prior to D15
            - The number and percentage who died and how many of these died prior to D15
    - The number and percentage of participants who **did not complete the primary assessment period**
        - Of these,
            - The number and percentage of participants who discontinued active treatment
                - Due to Adverse Event
                - Due to withdrawing consent

          - Due to not meeting exclusion criteria
        - The number and percentage who did not complete D28 assessments
  - The number and percentage of participants who **withdrew from study**
    - Due to lost to follow-up
    - Due to withdrawing consent
  - The number and percentage who completed planned active treatment duration
  - The number and percentage who completed the D28 assessment
  - The number and percentage who completed 12-22 week follow-up visit
  - The number and percentage of participants who died

The number and percentage of participants who discontinued due to AEs will be summarized separately for all participants, based on the FAS. This will be used for European Union Drug Regulating Authorities Clinical Trials (EudraCT) reporting.

In addition, the following listings will be presented:

- Study participant disposition (ES)
- Study discontinuation (ES)
- Analysis sets (ES)

The listing of study participant disposition will be presented by treatment group and by site, and will include the date of informed consent, date and time of first and last dose of study treatment, number of days on treatment, date of decision to terminate study treatment early and primary reason, date of D28 visit, date of the follow-up visit (if completed).

The number of days on study treatment will be calculated as follows:

$$\begin{aligned} &\mathit{Number\ of\ Days\ on\ Study\ Treatment} \\ &\quad = (\mathit{Date\ of\ Last\ Dose\ Received} - \mathit{Date\ of\ First\ Dose\ Received}) + 1 \end{aligned}$$

5.2 Protocol deviations

Important protocol deviations will be identified and classified by the deviation types defined in the IPD document, as per Section 3.4.

A listing of all IPDs identified at a data review meeting and will be presented for all study participants based on the FAS and will include the deviation type and description. The number and percentage of participants in the FAS with IPDs will be summarized by treatment group and for all participants for each deviation type. The number and percentage of participants who were excluded from the PPS will also be presented. The denominator for the percentage calculations will be the number of participants in the FAS.

6 DEMOGRAPHICS AND OTHER BASELINE CHARACTERISTICS

Additional endpoints to those mentioned below may be collected at baseline and will be summarized appropriately.

6.1 Demographics

A by-participant listing of demographics will be presented based on the ES. This will include the year of birth, age (calculated in years from date of randomisation), sex, race, height (in cm), weight (in kg) and body mass index (BMI, in kg/m2). The height will be the measurement obtained at the Screening visit. Weight will be the last non-missing value prior to the first dose of study treatment.

The BMI will be derived using the height and weight measurements recorded at the Screening visit and will be reported to 1 decimal place.

All demographic characteristics will be summarized by treatment group using the FAS. The summary of age will include descriptive statistics and categorical summaries, the latter based on requirements for EudraCT and clinicaltrials.gov reporting.

For the EudraCT reporting, the categories will include:

- 18 to <65 years
- 65 to <85 years

For clinicaltrials.gov reporting, the categories will include:

- ≤18 years
- 19 to <65 years
- ≥65 years

No statistical analysis will be performed on demographic data.

6.2 Other Baseline characteristics

All other baseline characteristics collected based on eligibility criteria will be listed for the FAS.

The following baseline characteristics will be summarized by treatment group using the FAS

<u>COVID-19 tests</u>

- Number and percentage on COVID-19 ward and ICU
- Number and percentage with positive PCR for COVID-19
- Number and percentage of Multiple PCRs
- Number and percentage of each type of sampling for COVID-PCR
    - Nasopharyngeal
    - BAL (bronchoalveolar lavage)
    - Anal

    - Sputum
    - Blood
    - Urine
    - Other (specify)
- Number and Percentage of PCR for other micro-organisms
    - Number and percentage positive for
        - Influenza
        - RSV
        - Other (Specify)
- Number and percentage of positive antigen tests for COVID-19
- Number and percentage of serology/other validated COVID-19 diagnostic tests

- Number and percentage of hypoxic respiratory failure

BASELINE ORDINAL SCALE Category

- Number and percentage with each score: 1 Death, 2 Hospitalized, requiring ECMO, invasive mechanical ventilation, or both, 3 Hospitalized, requiring nasal high-flow oxygen therapy, non-invasive mechanical ventilation, or both, 4 Hospitalized, requiring supplemental oxygen, 5 Hospitalized, not requiring supplemental oxygen, 6 Not hospitalized
- Note: these are worded slightly differently on the CRF

CYTOKINE RELEASE SYNDROME

- Summary of absolute score and probabilities for presence of sHLH by treatment arm (involves mapping the provided HScore to the appropriate point on the probability scale using linear interpolation to a probability based on the information contained in the following table)

HScore	Probability of hemophagocytic syndrome
≤90	<1%
91-100	~1%
101-110	1-3%
111-120	3-5%
121-130	5-9%

131-140	9-16%
141-150	16-25%
151-160	25-40%
161-170	40-54%
171-180	54-70%
181-190	70-80%
191-200	80-88%
201-210	88-93%
211-220	93-96%
221-230	96-98%
231-240	98-99%
≥241	>99%

Note: the best cutoff value for HScore was 169, corresponding to a sensitivity of 93%, specificity of 86%, and accurate classification of 90% of the participants.

Reference: https://www.mdcalc.com/hscore-reactive-hemophagocytic-syndrome#evidence

- Number and percentage of BOTH highly suspect bilateral ground opacities on chest CT-scan AND typical clinical and chemical diagnosis with signs of cytokine release syndrome
- Number and percentage of Radiography showing bilateral infiltrates in last 2 days (should be 100%)
- Number and percentage of signs of Cytokine Syndrome (should be 100%)
    - As shown by:
        - Number and percentage of single ferritin >2000 ng/ml (denominator participants with high flow nasal oxygen or mechanical ventilation i.e. total of participants on ordinal score 2 or 3 (which should be our participant population)) OR
        - Number and percentage of Ferritin > 1000 ng/ml and rising since last 24 hours

          OR
        - Lymphopenia < 800 /µL AND ≥ 2 of:
            - Ferritin > 700 mcg/L and rising since last 24h

    - CRP > 70 mg/dL and rising since last 24h without bacterial infection
    - LDH > 300 IU/L and rising since last 24h
    - D-dimers > 1000 ng/mL and rising for 24h

  OR

- Lymphopenia < 800 /µL AND ≥ 3 of:
    - Ferritin > 700 mcg/L
    - CRP > 70 mg/dL
    - LDH > 300 IU/L
    - D-dimers > 1000 ng/mL

Summary statistics and distribution plots of laboratory parameters listed here will be provided by treatment arm.

6.3 Medical history and concomitant diseases

A listing of all basic clinical data will be provided (such as prior lung diseases, smoking history, prior lung function measurements (within last 5 years), prior gas exchange measurements). These data will be collected from electronic medical records for each participant, and may not entered directly into the eCRF. All data received will be summarized as their data type suggests (see Section 3.1) by treatment arm.

The frequency of historical and ongoing conditions will be summarized by treatment (Y/N/ongoing).

Prior lung function test carried out (within last 5 years) (Y/N)

In addition to the above, any continuous data collected on lung function will be summarized by treatment arm (eg FEV1 (L)

Summary of substance use (alcohol (No, Daily, Sporadic, smoking (Never, Former, Current) or Other (No, Yes (other)) will be provided by treatment arm. Quantities may be summarized depending on quality of data provided.

6.4 Prior and concomitant medications

Prior medications include any medications that ended prior to the date of the first dose of study treatment.

Concomitant medications include medications with a start date before, on or after the first dose of study treatment and either ended post-first dose or continued after study treatment discontinuation.

Any medication that started prior to the first dose and stopped or continued after study treatment discontinuation after will be classified as both prior and concomitant.

Any medications with partially missing dates will be handled as described in Section 4.2.3 to classify them as prior or concomitant.

Medication use, both prior and concomitant, and all standard of care drugs (SOC) for Arm B will be collected on eCRFs. Drugs considered as SOC and the concomitant antibiotics, as opposed to prior and/or concomitant medication, will be clearly indicated and listed and summarized as described in Section 10.1 Extent of Exposure.

If the data allow, all prior and concomitant medications will be listed for the SS by treatment group and by WHODD Anatomical Main Group [Level 1 term text], Pharmacological Subgroup [Level 3 term text] and PT. The reported term will be included in the listing. Additional endpoints to be listed will be described in the TFL shells.

Separate summaries will be presented for prior medications and concomitant medications. Prior medications which continued into the Treatment Period will also be classified as concomitant and will be included in both summaries.

If medication is not coded, then all summaries will be based on the reported term.

If coded, all tabulations will be sorted alphabetically by Level 1 term, alphabetical Level 3 term within Level, otherwise by reported term, and decreasing frequency of PT in all study participants.

A glossary of all prior and concomitant medications will be presented for the FAS including the Anatomical Main Group (Level 1), Pharmacological Subgroup (Level 3), PT and reported term.

7 MEASUREMENTS OF TREATMENT COMPLIANCE

Administration of study treatment (including the administration of appropriate antibiotics) will be performed at the study site under the supervision of the designated site personnel. Compliance will be monitored at the site through a review of the pharmacy and dispensing records to ensure that each study participant received the correct dose level.

Compliance with taking oral daily antibiotics for at least 14 days after hospital discharge in Arm A will be monitored during the planned phone call at D28. The taking of antibiotics after hospital discharge is not required for Arm B.

Any dosing deviations will be reviewed as IPDs at the DEM and assessed for possible impact on the PPS. If considered important, these deviations will be included in the summary and listing of IPDs.

8 EFFICACY ANALYSES

All raw data collected will be displayed graphically, over time (relative day as continuous and nominal day as categorical) and by treatment arm, as appropriate. Plots may be trellised by factors to explore for potential patterns if there are too many data for meaningful review in a single plot e.g. by treatment arm for each site. The y-axis may be logarithmic or natural, depending on the endpoints being visualized. Means (and 95% CI) and Median and IQ range will be included where possible on the plots – this may not be appropriate when using relative day on the x-axis, depending on the number of participants who had measurements taken on each day, but should be possible for all plots using Nominal Day on the x-axis. If the data are presented on the log scale, then the geometric mean and 95% CI will be displayed, instead of the mean and 95% CI.

Of general note, since the protocol is not written using an estimand framework, this SAP will describe primary analyses for both primary and secondary endpoints. In addition, some pre-planned sensitivity and supplementary analyses are described.

In this context a sensitivity analysis is an additional analysis carried out on the same endpoint to determine how robust inferences based on the endpoint are to limitations in the data and assumptions behind the model used in the primary analysis. The analyses could use different transformations of the raw data, using different variance-covariance structures and different methods for handling missing data.

A supplementary analysis to support any clinical inference could use different methods of statistical analysis and/or different, but related, endpoints.

8.1 Statistical analysis of the primary efficacy endpoint(s)

The primary efficacy endpoints are PaO_2/FiO_2 ratio, P(A-a) O_2 gradient and a/A PO_2 ratio and of interest are comparisons between treatments of:

- Change from baseline to nominal D6in PaO_2/FiO_2 ratio (units=mm Hg)
- Change from baseline to nominal D6 in P(A-a) O_2 gradient (units=mm Hg)
- Change from baseline to nominal D6in a/A PO_2 ratio (unitless)
- Change from baseline to nominal D15in PaO_2/FiO_2 ratio
- Change from baseline to nominal D15 in P(A-a) O_2 gradient
- Change from baseline to nominal D15in a/A PO_2 ratio

Mapping of results to nominal days in the event that a participant is discharged from hospital before nominal D6 and/or nominal D15 is described in Section 3.2.1.3.

Residual plots, including plots against potential covariates, will be produced to investigate the validity of the assumptions behind the analyses for each of the 3 primary endpoints and if necessary, sensitivity analyses will be carried out (Section 8.1.3). Results from the most appropriate analysis will be presented.

8.1.1 Derivations of primary efficacy endpoint(s)

Measurements to derive PaO_2/F_iO_2 ratio (measure of severity of hypoxemia), P(A-a) O_2 gradient (always positive and a reflection of the difficulty oxygen has at crossing the alveolar capillary membrane and increases with age and F_iO_2 related to determine if it is normal.) and a/A PO_2 ratio are to be taken at Baseline, and at least at pre-dose (for arm A) on Day 6 and Day 15 (or at discharge, whichever comes first). Measurements for Arm B can be taken at any time, but preferably at the same time on each Day for every participant to allow for diurnal variation, at least on Days 1, 6, 15 (or at discharge, whichever comes first). Where possible, more frequent measurements will be taken in both arms, preferably daily.

eCRFs indicate that FiO_2 will be taken daily, however PaO_2 and $PaCO_2$ only at Screening/Day-1, nominal D1, nominal D6 and nominal D15.

Therefore, the derived parameters should be calculated using the data from the arterial blood gas (ABG) eCRF and not from Vital Signs.

Calculation

PaO_2 is the partial pressure of oxygen in the **arterial blood** and provided on the CRF. The Alveolar Gas Equation provides an estimate of PAO_2.

$PAO_2 = (P_{atm} - P_{H20})FiO_2 - PaCO_2/RQ$

(=partial pressure of oxygen in the **alveoli**)

Where:

P_{atm} is atmospheric pressure at sea level = constant 760 mm Hg

P_{H2O} is partial pressure of water in alveoli= constant 45 mm Hg

F_iO_2 is fractional concentration of O_2 in inspired air, from eCRF (or 21% (0.21) if participant is not on supplemental oxygen

$PaCO_2$ is partial pressure of carbon dioxide in alveoli from eCRF

RQ = respiratory quotient = constant 0.82 (for typical human diet)

P(A-a) O_2 gradient = A-a gradient = $PAO_2 - PaO_2$

a/A PO_2 ratio = PaO_2/PAO_2

Given that ABG is only measured on specified days, change from baseline to each of these days will be calculated for each of these endpoints for each participant [For example, (D6 - Baseline), (D15 - Baseline)].

Note:

All the above calculations should be carried out and used to verify the result for variables that are directly collected on the eCRF. Any discrepancy should be listed but the ones on the eCRF should be primarily used.

Increasing values for PaO_2/F_iO_2 ratio indicates improvement over time (normal considered ~470 mmHg)

Decreasing values of P(A-a) O_2 gradient indicates improvement over time (normal A-a gradient is (Age/4) +4) and in addition increases with higher FiO_2

No similar statement for a/A PO_2 is appropriate.

8.1.2 Primary analysis of the primary efficacy endpoint(s)

Summary descriptive statistics for each endpoint and change from baseline for each endpoint, including to follow-up, will be provided by treatment and Nominal Day (D1(Baseline), D2, D6, D15 and Follow-Up).

Each endpoint (PaO_2/FiO_2 ratio, P(A-a)/O_2 gradient, a/A PO_2 ratio) will be analyzed separately using a Mixed Model Repeated Measures (MMRM) analysis with fixed effects for Baseline, treatment, Nominal Day (using Nominal Days, D6 and D15), Baseline*Nominal Day interaction and treatment*Nominal Day interaction where Nominal Day is categorical. Participant will be fitted as a random effect and an unstructured covariance will be used, unless it fails to converge,

when other covariance structures will be considered. Mean change from baseline to D6 and D15 for each treatment arm and differences between treatments in mean change from baseline to D6 and D15, their 95% confidence intervals and p-values will be estimated directly from the model.

The primary analysis will not adjust for missing data, other than how they are handled in MMRM under the assumption that the data are missing at random. This assumption implies that the behaviour of the post dropout observations can be predicted from the observed endpoints, and therefore the response can be estimated without bias using exclusively the observed data.

The following results from the model will be tabulated:

- LSMEANS (adjusted for baseline levels) and 95% confidence intervals for change from baseline by treatment and Nominal Day for each endpoint (PaO_2/FiO_2 ratio, P(A-a)/O_2 gradient, a/A PO_2 ratio)
- Estimates of the differences between treatments in mean change from baseline, 95% confidence interval and p-value at D6 and D15 for each endpoint (PaO_2/FiO_2 ratio, P(A-a)/O_2 gradient, a/A PO_2 ratio).
- Mean percentage change from baseline will be estimated from the results of the MMRM model for D6 and D15 for each Treatment Arm, together with 95% confidence intervals.

Plots of the results from the MMRM will be provided for LSMEANS (adjusted) of change from baseline and 95% CI by treatment on each Nominal Day of Interest and the mean differences of the LSMEANS between treatments and their 95% CI. Distribution plots for the estimated mean differences (Arm A -Arm B) between treatments will be provided for each nominal day where the x-axis will represent the range of differences between treatments and the area under the curve will present the probability of the mean difference in means being greater than specified values.

8.1.3 Sensitivity analyses of the primary efficacy endpoint(s)

In order to explore the validity of assumptions behind the primary analyses and after review of residual plots, the data for each endpoint (data only come from the set of positive real numbers) (PaO_2/FiO_2 ratio, P(A-a)/O_2 gradient, a/A PO_2 ratio) may be first log transformed and each endpoint will be analysed separately using a MMRM model with fixed effects for Baseline, treatment, Nominal Day (D1,D6, D15), Baseline*Nominal Day interaction and treatment*Nominal Day interaction where Nominal Day is categorical. Participant will be fitted as a random effect and an unstructured covariance will be used, unless it fails to converge, when other covariance structures will be considered.

Mean change from baseline to D6 and D15 for each treatment arm and differences in the means on the log scale between Arm A and Arm B (Arm A- Arm B) for each VISIT (Day 6, 15) will be estimated directly from the models.

The following results from the model will be first back-transformed and then tabulated:

- LS geometric means (adjusted for baseline) and 95% confidence intervals for by treatment and Nominal Day for each endpoint (PaO_2/FiO_2 ratio, P(A-a)/O_2 gradient, a/A PO_2 ratio) to provide geometric LSMEANS(adjusted for baseline) and 95% confidence limits

- Estimates of the treatment differences on the log scale between treatments and 95% confidence interval at D6 and D15 for each endpoint (PaO_2/FiO_2 ratio, P(A-a)/O_2 gradient, a/A PO_2 ratio) to provide the ratio of geometric means and 95% confidence limits. The p-values for these differences will also be provided in the table.

These analyses will be repeated using the alternate baseline (section 3.2.2.2) to include participants with a baseline soon after dosing.

An additional sensitivity analysis will repeat both the primary analysis and sensitivity analysis above using LOCF for participants with missing data on either D6 or D15 that was not due to discharge:

- due to death prior to D15, which could mean both D6 and D15 are missing or just D15. The impact of both being missing will be explored using residuals, as the LOCF method will necessarily mean that the change from baseline for these participants is nominally 0, as the baseline value will be carried forward

To explore the impact of age, sex, weight and ethnicity on the results, the analyses described above and in section 8.1.2 will be repeated including sex, age (in years), BMI and weight and ethnicity as additional covariates.

8.2 Statistical analysis of the secondary efficacy endpoint(s)

There are several secondary endpoints that cover each of the secondary objectives and a list is provided in the table listing the secondary endpoints in Section 2.2.1.2. Some of these endpoints are different derivations from the same collected data. In addition, some of the endpoints collected for one objective, may be the inputs to derivations for endpoints for the same objective or for another objective.

Data types for the secondary efficacy endpoints fall into the following main categories: continuous (for example, change from baseline (including the various ordinal categorical endpoints), number of days with ‘condition’), ordinal categorical, binary (‘responder’ Y/N), time to event or duration of event.

Table 8.1 describes the analyses of each secondary endpoint listed in Section 2.2.1.2 and identifies which efficacy objective (2-6) the endpoint is addressing. Brief details of any derivations and analysis methods used are provided. Further details are included in the mock TFL shell document. In the event the data for some of these endpoints indicate that analysis through fitting models is not appropriate, for example too few events for a formal analysis on response or mortality rates, or time to event analyses, the analyses will not be carried out and only listings, figures and summaries provided.

Some patients may withdraw from the study or be transferred to another hospital prior to discharge through medical improvement. If data have been collected from another hospital post discharge from the first hospital, these data will be listed. This will be for variables or derived variables which would have included data from these patients. For example, duration of stay in hospital. However, for variables only including data up to a relative day prior to discharge/transfer from the first hospital, these patients will be included in the analysis/summary

for that period but set as missing for the other periods. Residual plots will be reviewed to determine any influence these participants may have had on the results.

Table 8.1

Endpoint	Derivation	Analysis
Objective 2<br><br>Incidents of participants reporting each severity rating in 6-point ordinal scale at D1, D6, D15 and D28	Percentages for each ordinal score on each day are based on N in each treatment arm on D1. An additional category (score=7) to be created to indicate participants who have withdrawn from the study for any reason or transferred to another hospital. Add label for score category=7 as 'Withdrawn'. Participants who die (score=1) or are discharged from hospital due to medical improvement (score=6) or are withdrawn (score=7) remain in both the numerator and denominator for these categories on subsequent days by using LOCF for discharge, death and withdrawal. Only use LOCF for participants whose final recorded score is 1, 6 or 7. For the interim, all other subjects whose final recorded score during the period up to 28 days is not equal to 1, 6 or 7, impute a score of 8 for their remaining days (up to D28). This is to indicate the percentage of participants who are still on treatment without imputing what their future values may be.<br><br>Check that ordinal score matches whether participant has been discharged or has died	PROC FREQ to create **summary table of actual counts and percentages for each of the 6-points on the ordinal scale on each nominal day (D1, D6, D15 and D28) for each treatment**<br><br>**Stacked 100% bar chart indicating a snapshot of counts in each category (color coded for scores 1-7 (including 8 in the interim analyses)) on each relative day by treatment arm. A separate graph for each treatment, placed side by side.**<br><br>**<u>For final analysis:</u>**<br><br>Logistic regression for data from specified nominal days (D1, D6, D15 and D28). Fixed effect of Score*Treatment indicating whether the pattern across scores is different between treatments on each day.<br><br>Note: If D1 is pre-dose and therefore baseline, exclude from table and plot.

Endpoint	Derivation	Analysis
Objective 2<br>Change from baseline in 6-point ordinal scale to Day 6, Day 15 and Day 28	Change from baseline at each relative day for each participant. If a participant has died (score=1) or discharged from hospital (score=6), these values will carry forward onto subsequent days. For participants who are missing values after any day for any other reason, the value will remain missing. For participants who miss some intermediate values, the latest value prior to the missing value will be carried forward until the day where data are recorded again.<br>The additional scores derived for the previous endpoint will not be used in change from baseline<br>(One line per day per participant dataset)	**Summary table providing n, mean change from baseline, SD change from baseline, median, minimum and maximum on each specified nominal day by treatment arm**<br><br>**Graph of change from baseline over time for each participant by treatment, and the average lines for each treatment.**<br>For final analysis: MMRM fitting response variable change from baseline against fixed effects for specified nominal days, treatment and treatment*nominal day interaction. Baseline score as covariate and baseline score*nominal day as additional terms. Participant as random effect.
Objective 2<br>Time to at least a 2-point improvement or discharge sustained up to (D15 for interim analysis 1) D28 on the 6-point ordinal scale during 28-day assessment period (=Response)<br>Note: for both the interim analyses and final analyses up to D15 and D28 respectively, if the 2-point change occurred on D15 or D28, and the	Create TTE dataset for cumulative response rate analysis with 2 variables each for analysis up to D15 and up to D28 with one line per participant providing time to response and censor status. Censor status=0 for patients with response and =1 on relative day for: patients who have: withdrawn from treatment and/or study; discharged from first hospital for reasons other than improvement; have no response by analysis date (censor=1 at analysis	**Kaplan-Meier analysis using PROC LIFETEST.**<br>**Strata ACTARM.**<br>**Request Failure plot adjusting axis and heading titles to reflect responder and not failure**<br>**Output statistics:**<br>**Appropriate statistics from analysis as indicated in mock shell**

Endpoint	Derivation	Analysis
patient remained in hospital, the patient will be classified as a responder, even though the 2-point change had not been ‘sustained’.	date). If Time to Response >15 and censor=1 for analysis up to D15, Time to Response=15. If Time to Response >28 and censor=1 for analysis up to D28, Time to Response=28. For patients who have died Time to Response is set to D15 or D28 respectively and censored at this time. Note: Treatment will be merged into the TTE dataset. In addition to other variables that may be used as covariates in final analysis.<br>(one line per participant dataset)	
Objective 2<br>Percentage of participants reporting at least a 2-point improvement sustained to D28 or discharge	Use D28 response indicator variable defined above	PROC FREQ/PROC LOGISTIC(if covariates are added)<br>Model: Response=ACTARM +covariates (if added)<br>Output percentage response on each treatment.<br>Odds ratio and 95% confidence interval
Objective 2<br>AUEC/Time using the 6-point score recorded daily – up to D15, D28 and all data recorded	For each participant calculate the area under the curve and divide by 15 for up to D15 and by 28 for up to D28 (higher average score per day the better the response). Use last value brought forward for any missing values until next data entry. For discharge, carry forward score=6 for each day post discharge day. For death, carry forward score=1 for each day post death day.	Analyse AUEC/Time using analysis of variance with ACTARM as a two-level factor and baseline score as a covariate

Endpoint	Derivation	Analysis
Objective 2<br>Percentage of participants who record at least 2-point improvement from baseline or discharge (response) between D1-D6, D7-D15 and D16-D28	Responder classified as above. Create indicator variable (3 levels) if the relative day of START of response is between D1 and D6, between D7 and D15 or between D16 and D28. Each responder participant can only be within one of these categories. Participants who have a missing value are classified as non-responders	**Frequency table and percentage responders in each of the categories defined by the indicator variable, by treatment. Exact 95% confidence intervals for each percentage**
Objective 2<br>Percentage of participants not deteriorating according to the ordinal scale by 1 point between D1and D6, between D7 and D15 and between D16 and D28 – not deteriorating corresponds to Stable + Responders	A participant is classified as Stable + Response if at no time CfB < -1. Create indicator variable (3 levels) if the relative day of START of Stable or Response falls between D1and D6, between D7 and D15 and between D16 and D28 for each participant	Frequency table and percentage responders in each of the categories defined by the indicator variable, by treatment. Exact 95% confidence intervals for each percentage
Objective 2<br>6-point ordinal Scale at D6 and D15 and D28 in relation to D-Dimers and complement C5a levels at baseline		Box-whisker plot of baseline D-Dimers and complement C5a for each score (x-axis) reported on each of D6, D15 and D28
Objective 2<br>Change from baseline in SOFA score (https://www.mdcalc.com/sequential-organ-failure-assessment-sofa-score)	Change from baseline to D6 and D15 in SOFA score	Summary statistics for raw data and change from baseline

Endpoint	Derivation	Analysis
to Day 6, Day 15 (or at discharge, whichever comes first)		
Objective 2<br>Number of days with fever (defined as 37.1ºC or more) during 28-day assessment period	Create indicator variable for each participant on each day where temperature is above 37.1 ºC<br>Create variable with total number of days where temperature was above 37.1 ºC for each participant<br>Create variable for each participant giving total number of days where temperature was recorded	Summary statistics to provide n, average number of days, SD, median, minimum and maximum where temperature was above 37.1 ºC by treatment<br>Produce line plot of temperature over time for each patient together with average line per treatment group.<br>Temperatures are due to be taken daily until discharge – indicate the number of subjects on each day in each treatment arm
Objective 2<br>Time since randomization until absence of LAST fever (defined as 37. 1ºC or more) for more than 48 h without antipyretics (use last fever to account for possibility that participants have intermittent fever per this definition)	Antipyretic use in last 24 hours is collected on eCRF on every day. Use highest temperature recorded in last 24 hours, not the 7-10am temperature to identify the date when the last fever ended per definition.<br>Create variable which contains number of days between this date and date of randomisation for each participant. If a participant has had no episodes of the fever, the number of days is 0.<br>Create indicator variable (censor) to indicate if participant has recovered from the last fever at time of analysis (0=recovered, 1=fever is ongoing at time of analysis). If a participant has had no fever, (i.e. time=0), censor=0.	Kaplan-Meier analysis using PROC LIFETEST.<br>Strata ACTARM.<br>Request Failure plot adjusting axis and heading titles to reflect responder and not failure<br>Output statistics:<br>Appropriate statistics from analysis as indicated in mock shell

Endpoint	Derivation	Analysis
Objective 2<br>Duration of hospital stay<br><br>Duration of hospital stay of survivors	Calculate duration of hospital stay in days (Hospital Discharge date-Hospital Admission date)<br>Create indicator variable to flag survivors	Summary statistics: n, nmissing, mean, SD, median, minimum, maximum<br>By treatment arm
Objective 2<br>Number of days requiring supplemental oxygenation after randomisation to Day 28 (or discharge, whichever comes earlier)	Number of days where Suppl O_2 = 'Y' from Vital parameters	Summary statistics: n, nmissing, mean, SD, median, minimum, maximum<br>By treatment arm
Objective 2<br>Time since randomization until improvement in oxygenation (defined as independence from supplemental oxygen) during 28 day assessment period (or discharge, whichever comes earlier)	Independence from supplementary oxygen means no further 'Y' after a 'N'	Kaplan-Meier analysis using PROC LIFETEST.<br>Strata ACTARM.<br>Request Failure plot adjusting axis and heading titles to reflect responder and not failure<br>Output statistics:<br>Appropriate statistics from analysis as indicated in mock shell
Objective 2<br>Number of days with hypoxia (defined as SpO2 <93% breathing room air or the dependence on supplemental oxygen after randomisation to Day 28 (or discharge, whichever comes earlier)	Use SpO2 (%) and Supp O2 (Y/N) from Vital parameters to derive indicator variable which flags each day where this condition holds.<br>Derive variable per participant that gives total number of days with condition	Summary statistics of variable with Total number of days: n, mean, SD, median, minimum, maximum<br>By treatment arm

Endpoint	Derivation	Analysis
Objective 3<br>Duration of ventilator free days after randomisation to Day 28 (or discharge, whichever comes earlier)	Use data from Vital parameters (include all days, where NIV, IV and ECMO='N')	Summary statistics of variable with Total number of days: n, mean, SD, median, minimum, maximum<br>By treatment arm
Objective 3<br>Duration of invasive mechanical (including ECMO) and non-invasive mechanical ventilation in ventilated participants	Complement of above (exclude participants who are not ventilated i.e. do not include these with a duration of 0)	Summary statistics of variable with Total number of days: n, mean, SD, median, minimum, maximum<br>By treatment arm
Objective 3<br>Time since randomization until first use of high-flow oxygen devices or non-invasive mechanical ventilation or invasive mechanical ventilation or ECMO in non-ventilated participants (i.e. excluding participants who are ventilated within 24h prior to or after randomization) during 28-day assessment period	Use Vital Parameter CRF and the method of supplementary oxygen variable. Use the date from this CRF to identify when first use occurred.<br>Indicator variable to identify sub-group to be included.<br>Censor if never used any of these ventilation methods at Day 28	Kaplan-Meier analysis using PROC LIFETEST.<br>Strata ACTARM.<br>Request Survival plot adjusting axis and heading titles to reflect progression and not response<br>Output statistics:<br>Appropriate statistics from analysis as indicated in mock shell
Objective 3<br>Time since randomization to progression to ARDS	Randomisation date<br>Date of ARDS diagnosis<br>Derive time variable (days) from date of randomization to date of ARDs diagnosis for each participant. For participants who	Kaplan-Meier analysis using PROC LIFETEST.<br>Strata ACTARM.<br>Request Survival plot adjusting axis and heading titles to reflect progression and not response

Endpoint	Derivation	Analysis
	do not progress to ARDs, time is from date of randomization to date of discharge from hospital or 28 days, whichever is earlier.<br>Derive Censor variable indicating whether Progression to ARDS (censor=0, otherwise censor=1)	Output statistics:<br>Appropriate statistics from analysis as indicated in mock shell
Objective 3<br>Duration of ICU stay in participants that enrolled in trial that were on invasive or non-invasive mechanical ventilation or high-flow oxygen devices within 24h prior to or after randomization during 28-day assessment period<br>Duration of ICU stay in participants that enrolled in trial that were on invasive or non-invasive mechanical ventilation for less than 24h prior to or after randomization during 28-day assessment period	Use DATES and Vital Signs dataset to calculate variable giving total number of days in ICU for sub-group of patients	Summary statistics of variable with Total number of days: n, mean, SD, median, minimum, maximum<br>By treatment arm

Endpoint	Derivation	Analysis
**Objective 6**<br>**All-cause mortality rate at 28 days post randomisation (all treated participants)**<br><br>All-cause mortality rate at 28 days post randomisation (excluding participants that required invasive mechanical ventilation or ECMO, non-invasive mechanical ventilation or high-flow oxygen devices within 24 hours prior to or after randomisation)<br><br>All-cause mortality rate at 28 days post randomisation (excluding participants that required invasive mechanical ventilation or ECMO within 24 hours prior to or after randomisation)<br><br>All-cause mortality rate at 28 days post randomisation (excluding participants that required invasive mechanical ventilation or ECMO, non-invasive mechanical ventilation or high-flow oxygen devices within 24 hours prior to or after randomisation)<br><br>All-cause mortality rate at 28 days post randomisation (only including participants that required invasive	**Using 6 point ordinal scores, DATES and VITAL SIGNS and SuppO2 variables :**<br>Derive indicator variable identifying each sub-group of patients identified in the Endpoint column<br>Derive Mortality variable identifying whether a patient has died within 28 days of randomisation	For each mortality sub-group:<br>PROC LOGISTIC<br>Model: Mortality=ACTARM<br>Output percentage died on each treatment and 95% confidence interval.<br>Odds ratio and 95% confidence interval

Endpoint	Derivation	Analysis
mechanical ventilation or ECMO within 24 hours prior to or after randomisation)<br><br>All-cause mortality at follow up 12-22 weeks post-randomisation for all treated participants		
Objective 5<br>Incidents of participants in each category of the 6-point ordinal scale at follow-up 12-22 weeks post randomisation	6-point ordinal score	PROC FREQ to create summary table of actual counts and percentages for each of the 6-points on the ordinal scale at follow-up by treatment arm
Objective 5<br>Incidents of lung fibrosis on chest high-resolution computed tomography (HRCT) scan at follow up 12-22 weeks post-randomisation<br><br>Incidents of lung function abnormalities at follow up 12-22 weeks post-randomisation	HRCT results<br><br>Lung function results – dataset should list all abnormalities in a consistent way across participants, including 'None'	Summarise Left lower, Left middle and Left Upper Total Scores by treatment<br>Summarise Right lower, Right middle and Right Upper Total Scores by treatment<br>Provide 100% stacked bars for each of the above 6 areas, coding by score (normal, reticular, bronch and honeycomb) – a separate graph for each treatment
Objective 5<br>Results from the 6-minute walk test		

Endpoint	Derivation	Analysis
Objective 5<br>Incidents of participants in each category of the WHO performance scale	WHO score	Summary statistics of WHO Score by treatment at follow-up
Objective 4<br>Incidence of nosocomial bacterial or invasive fungal infection during 28-day assessment period<br><br>Note: Participants with viral respiratory infection are at risk of secondary bacterial infections. As part of clinical routine care, sputum or BAL samples will be collected in participants suspected of secondary bacterial pneumonia and checked for presence of bacteria. Measurements of procalcitonin levels will be performed at least 3x/week until 14 days or hospital discharge	Procalcitonine will be concentrations. Concentrations are to be taken 3 times a week. The actual day each week may differ between participants. Allocate 1, 2 or 3 to each concentration taken in chronological order in each week.<br><br>Replace 'Not Dectectable' with the lower limit of quantification and include in the summary statistics.<br><br>Data for bacteria will be presence/absence at each day tested (if collected)	Summarise procalcitonine concentrations by occasion (1,2,3) within each period of 7 relative days post first-dose for each treatment.<br><br>Summary statistics to include n, nmissing, Mean, SD, Median, Minimum, Maximum. n in each case will be N-Not Done(nmissing).<br><br>Provide line plots over relative day by treatment. A separate line for each participant<br><br>Depending on distribution of data the above summary statistics will be provided using the log scale, and presented after back transforming
No specific objective<br>Change in ferritin levels between D1 and D6 and between D1 and D15 (or discharge in each case)<br><br>Change in CRP levels between D1 and D6 and between D1 and D15 (or discharge in each case)	Laboratory values – derive change from D1 (i.e the last pre-dose value) to D6 and D15 or day of discharge	Summarise change from D1 in concentrations to D6 and to D15 (or day of discharge) for each treatment.<br><br>Summary statistics to include n, nmissing, Mean, SD, Median, Minimum, Maximum. n in each case will be N-Not Done(nmissing).

Endpoint	Derivation	Analysis
		Provide line plots over relative by treatment, for both absolute values and change from D1. A separate line for each participant

9 PHARMACOKINETICS AND PHARMACODYNAMICS

9.1 Pharmacokinetics

Pharmacokinetic data are only collected pre-dose on Days 1, 2 and 6 in Group A. Concentration data will be listed and summarized.

9.2 Pharmacodynamics

Plasma and serum samples were collected for summary and exploratory analysis as appropriate (e.g. compare change from baseline between the two treatment arms). Listings, summaries graphs and figures for raw values and change from baseline (if appropriate) over time will be generated. Associations with response may be explored.

10 SAFETY ANALYSES

All safety summaries and listings will be presented by treatment group and overall based on the SS as defined in in Section 3.5 unless otherwise stated.

10.1 Extent of exposure

Details of study treatment administration will be listed, including administration of the concomitant antibiotics, by treatment arm and participant, ordered by date of administration within participant. This listing will include all medications that are considered as SOC for Arm B. The actual endpoints to be listed will be defined in the TFL shells, but at a minimum should contain date and time of each dose of Zilucoplan® and concomitant antibiotics for participants on Arm A, and for each of the SOC medications and concomitant antibiotics for participants on Arm B.

Frequency counts and percentages for the number of doses of study treatment received and summary statistics for the duration of exposure to study treatment will be presented overall and broken down by age (<75yr, >=75yrs) and sex. Duration of exposure will be calculated as follows:

$$Duration\ of\ Exposure = (Date\ of\ Last\ Dose - Date\ of\ First\ Dose)$$

Antibiotics after discharge for the Zilucoplan® arm were not collected by day (due to be taken for 14 days after discharge) in the CRF. Hence for Day 28-31 just the information of ‘were antibiotics’ taken (Y/N) will be listed, but the detail of compliance in this period will not be able to be captured.

10.2 Adverse events

An AE is any untoward medical occurrence in a participant or clinical investigation participant administered a pharmaceutical product that does not necessarily have a causal relationship with this treatment. An AE can therefore be any unfavorable and unintended sign (including an abnormal laboratory finding), symptom, or disease temporally associated with the use of a medicinal (investigational) product, whether or not related to the medicinal (investigational) product.

AEs are further characterised as pre-treatment or treatment emergent.

A pre-treatment AE are events whose onset date and time is prior to the first administration of study drug (Zilucoplan® for Arm A and IV antibiotic for Arm B).

A TEAE is defined as any AE with a start date/time on or after the first dose of study treatment up until the D28 visit. Any AE with an onset date later than the D28 visit for a participant will not be considered as treatment-emergent and therefore will not be included in the tabulations of TEAEs. Where dates are missing or partially missing, AEs will be assumed to be treatment-emergent, unless there is clear evidence to suggest that the AE started prior to the first dose of study treatment. Missing or partially missing dates for AEs will be handled as described in Section 4.2.3. All AEs for a participant will be recorded in the eCRF from the time of informed consent until completion or early study discontinuation.

For each AE the following information should be provided in the eCRF: AE term (verbatim), date and time of onset, whether or not the AE was classified as an SAE, relationship to study treatment, severity, action taken with study treatment, other action taken, outcome, date and time of outcome and whether the AE led to study treatment discontinuation or to study discontinuation.

COVID-19 infection is a very recent syndrome, on which few data are available. Therefore, normal symptoms and natural disease course symptoms that will not be reported as adverse events are dyspnea, coughing, malaise, fever, drop in oxygen saturation, progression to respiratory failure, progression to ARDS, drop in blood pressure. However, progression to multi-organ failure may have other etiologies and will be reported as an SAE.

The number and percentage of participants who experience TEAEs will be summarized by MedDRA preferred term (PT) and treatment.

Summaries of TEAEs may include the following:

- Incidence of overall TEAEs (overview including number and percentage of participants with any TEAEs, serious TEAEs, discontinuations due to TEAEs, drug-related TEAEs, severe TEAEs and TEAEs leading to death; event counts will also be included)
- Incidence of TEAEs
- Incidence of TEAEs by maximum intensity
- Incidence of TEAEs by maximum CTCAE grade
- Incidence of TEAEs by maximal relationship
- Incidence of non-serious TEAEs above reporting threshold of 5% of study participants

Summary tables will contain counts of study participant, percentages of study participants in parentheses and the number of events where applicable. A participant who has multiple events in the same SOC and PT during a given treatment will be counted only once in the participant counts for that treatment, but all events will be included.

In summaries including relationship, the following relationships will be summarized: Any,'Not related', 'Related'. eCRF entries of 'Not applicable' and 'Unlikely' will be classified as 'Not

Related'. eCRF entries of 'Possibly', 'Probably' and 'Definitely' will be classified as 'Related'. Participants who experience the same event multiple times will be included in the most related category for tabulations by maximum relationship. Events with missing relationship will be considered as 'Related' but recorded as missing in the listings.

In summaries including intensity using CTCAE v5.0 grades, the following intensity categories will be summarized: 'Grade 1 -Mild', 'Grade 2 - Moderate', 'Grade 3 -Severe', 'Grade 4 – life threatening', 'Grade 5 – Death'. Participants who experience the same event multiple times will be included in the most severe category for tabulations by maximum intensity. Events with missing intensity will be considered as 'Severe' events for summary purposes but recorded as missing in the listings.

Adverse event summaries will be ordered alphabetically by System Organ Class (SOC) and decreasing frequency of PT within SOC in the group column for tables including event counts. For tables including only number and percentage of study participants, summaries will be ordered alphabetically by SOC and decreasing incidence of PT within SOC in the group column.

Listings of AEs and TEAEs will include the following:

- All AEs
- Incidence of all TEAEs
- All Related TEAEs
- All Serious AEs
- Discontinuation due to AEs

All listings (except incidence of all TEAEs) will be presented by study participant and treatment and will include the onset date/time and outcome date/time of the event, the event duration (derived), time to onset (derived), pattern of event, intensity, relationship, action taken, outcome and AEs that led to discontinuation.

The listing of incidence of all TEAEs will be presented by treatment and will include intensity, relationship, serious, number of participants reporting at least one TEAE within SOC/PT, number of individual occurrences of TEAE and site-participant number.

10.3 Clinical laboratory evaluations

Clinical chemistry and hematology parameters may be summarized graphically and listed by treatment and timepoint for both absolute values and changes from baseline.

10.4 Vital signs, physical findings, and other observations related to safety

10.4.1 Vital signs

Vital signs measurements maybe summarized graphically and listed by treatment and timepoint including changes from Baseline.

10.4.2 Electrocardiograms (if applicable)

ECG data maybe listed.

11 REFERENCES

None at this time.

12. APPENDICES

None at this time.

13. AMENDMENT(S) TO THE STATISTICAL ANALYSIS PLAN (SAP) (IF APPLICABLE)

This is the first version of the SAP

STATISTICAL ANALYSIS PLAN SIGNATURE PAGE

This document has been reviewed and approved per the Review and Approval of Clinical Documents Standard Operating Procedures. Signatures indicate that the final version of the Statistical Analysis Plan (SAP) or amended SAP is released for execution.

Review and Approval of Clinical Documents
for reviewer(s)/approver(s) not captured in ECMS

Project code/Study Number:	Compound Name:	Indication (if applicable):
ZILU-COV	**Zilucoplan®**	

Document(s) submitted for: (check one)

	**Review**
X	**Approval**

Document(s) submitted: **Statistical Analysis Plan**

Version Date: **18December2020**

Submitted by: [redacted] (Printed Name and Date) **SSI - CTStat** (Department and Function)

Document distributed to: (Add rows as needed)

Role[a]	Name[b]
SPhys	[redacted]
Statistical Rep	[redacted]

[a] As defined within **sop-af-009924**
[b] Please name the person or persons to review or approve the document(s).

Signed by:

(Printed Author Name)

(Author Signature) (Date)

Signed by: [redacted]

(Printed Author Name)

[redacted] 18 DEC 2020

(Author Signature) (Date)

Note: For an <u>approval</u> not captured in ECMS, documentation of the approval (eg, printed Email) should be appended to this form.
