# Supplementary material for: Efficacy and safety of the investigational complement C5 inhibitor zilucoplan in patients hospitalized with COVID-19: an open-label randomized controlled trial
Source: Respir Res. 2022 Aug 9;23:202. doi: 10.1186/s12931-022-02126-2 (PMC9361275; doi:10.1186/s12931-022-02126-2)
Supplement: Supplementary file 3 — Additional file 3. Overview secondary and exploratory endpoints. [file 12931_2022_2126_MOESM3_ESM.pdf]

The SAP was approved prior to the final version of the protocol (v6). The protocol amendment did not affect the conduct of the analyses, so the SAP was not amended. However, some secondary endpoints were moved to exploratory. This table describes each endpoint and if it was conducted.

Objective	Endpoint	Secondary endpoint in v6 protocol	Conducted
1) To study if early intervention with Zilucoplan® affects clinical outcome defined by duration of hospital stay, 6-point ordinal scale, time to defervescence, supplemental oxygen use, SOFA score	Incidents of participants reporting each severity rating in 6-point ordinal scale at D1, D6, D15 and D28	N	Y
	Change from baseline in 6-point ordinal scale to Day 6, Day 15 and Day 28 (by phone call) weeks post randomisation	N	Y
	Time to at least a 2-point improvement or discharge sustained up to D28 on the 6-point ordinal scale during 28-day assessment period	Y	Y
	Percentage of participants reporting at least a 2-point improvement sustained to D28 or discharge	N	Y
	AUEC/Time using the 6-point score recorded daily – up to D15, D28 and all data recorded	N	N - not conducted as was a planned exploratory analysis for if a clear trend in clinical score had been observed
	Percentage of participants who record a 2-point improvement from baseline or discharge at D6, D15 and D28	N	
	Percentage of participants not deteriorating according to the ordinal scale by 1 or 2 points on D6, D15 and D28	N	
	6-point ordinal Scale at D6 and D15 and D28 in relation to D-Dimers and complement C5a levels at baseline	N	N- not conducted. No clear and pre-defined definition of 'high' vs 'low' C5/d-dimer at baseline
	Change from baseline in SOFA score to Day 6, Day 15 (or at discharge, whichever comes first)	Y	Y

1)continued:<br>To study if early intervention with Zilucoplan® affects clinical outcome defined by duration of hospital stay, 6-point ordinal scale, time to defervescence, supplemental oxygen use, SOFA score	Number of days with fever (defined as 37.1ºC or more) during 28-day assessment period	Y	Y
	Time since randomization until absence of LAST fever (defined as 37. 1ºC or more) for more than 48 h without antipyretics (use last fever to account for possibility that participants have intermittent fever per this definition)	N	Y
	Duration of hospital stay	N	Y
	Duration of hospital stay of survivors	N	Y
	Number of days requiring supplemental oxygenation after randomisation to Day 28 (or discharge, whichever comes earlier)	Y	Y
	Time since randomization until improvement in oxygenation (defined as independence from supplemental oxygen) during 28-day assessment period (or discharge, whichever comes earlier)	N	Y
	Number of days with hypoxia (defined as SpO2 <93% breathing room air or the dependence on supplemental oxygen after randomisation to Day 28 (or discharge, whichever comes earlier)	N	Y
2) To study if early intervention with Zilucoplan® affects	Duration of ventilator free days after randomisation to Day 28 (or discharge, whichever comes earlier)	N	Y

progression to mechanical ventilation and/or ARDS<br>And Duration of ICU stay	Duration of invasive mechanical (including ECMO) and non-invasive mechanical ventilation in ventilated participants	N	N - Not conducted
	Time since randomization until first use of high-flow oxygen devices or non-invasive mechanical ventilation or invasive mechanical ventilation or ECMO in non-ventilated participants (i.e. excluding participants who are ventilated within 24h prior to or after randomization) during 28-day assessment period	N	N - Time of day of ventilation was not routinely collected and hence the 24hr window could not be accurately calculated
	Time since randomization to progression to ARDS	N	Y
	-Time since randomization to progression to ARDS according to D-dimers and complement C5a at randomization	N	N - Omitted from the SAP, not conducted
	Duration of ICU stay in participants that enrolled in trial that were on invasive or non-invasive mechanical ventilation or high-flow oxygen devices within 24h prior to or after randomization during 28-day assessment period	N	N - Time of day of ventilation was not routinely collected and hence the 24hr window could not be accurately calculated
	Duration of ICU stay in participants that enrolled in trial that were on invasive or non-invasive mechanical ventilation for less than 24h prior to or after randomization during 28-day assessment period	N	
3) To study if treatment with Zilucoplan®	All-cause mortality rate at 28 days post randomisation (all treated participants)	Y	Y

affects all-cause mortality rate at day 28 and at 12-22 weeks post-randomisation	All-cause mortality rate at 28 days post randomisation (excluding participants that required invasive mechanical ventilation or ECMO, non-invasive mechanical ventilation or high-flow oxygen devices within 24 hours prior to or after randomisation)	Y	Y
	All-cause mortality rate at 28 days post randomisation (excluding participants that required invasive mechanical ventilation or ECMO within 24 hours prior to or after randomisation)	Y	Y
	All-cause mortality rate at 28 days post randomisation (excluding participants that required invasive mechanical ventilation or ECMO, non-invasive mechanical ventilation or high-flow oxygen devices within 24 hours prior to or after randomisation)	N	N - Typo in SAP - this was a repeat of second mortality grouping
	All-cause mortality rate at 28 days post randomisation (only including participants that required invasive mechanical ventilation or ECMO within 24 hours prior to or after randomisation)	Y	Y
	All-cause mortality at follow-up 12-22 weeks post-randomisation for all treated participants	Y	Y
4) To study if treatment with Zilucoplan® has a favourable effect on long term at follow up 12-22 weeks post-randomisation	Incidents of participants in each category of the 6-point ordinal scale at follow-up 12-22 weeks post randomisation	Y	Y
	Incidents of lung fibrosis on chest high-resolution computed tomography (HRCT) scan at follow up 12-22 weeks post-randomisation	N	Y

	Incidents of lung function abnormalities at follow up 12-22 weeks post-randomisation	N	Y
	Results from the 6-minute walk test	Y	Y
	Incidents of participants in each category of the WHO performance scale	Y	Y
5) To study if early intervention with Zilucoplan® affects the rate of nosocomial infection	Incidents of nosocomial bacterial or invasive fungal infection during 28-day assessment period	Y	Y
	Note: Participants with viral respiratory infection are at risk of secondary bacterial infections. As part of clinical routine care, sputum or BAL samples will be collected in participants suspected of secondary bacterial pneumonia and checked for presence of bacteria. Measurements of procalcitonin levels will be performed at least 3x/week until 14 days or hospital discharge	N	Y
6) Change from baseline in specific labs	Mean change in ferritin levels between D1 and D6 and between D1 and D15 (or discharge in each case)	N	Y
	Mean change in CRP levels between D1 and D6 and between D1 and D15 (or discharge in each case)	N	Y
