# Supplementary material for: Efficacy and safety of the investigational complement C5 inhibitor zilucoplan in patients hospitalized with COVID-19: an open-label randomized controlled trial
Source: Respir Res. 2022 Aug 9;23:202. doi: 10.1186/s12931-022-02126-2 (PMC9361275; doi:10.1186/s12931-022-02126-2)
Supplement: Supplementary file 4 — Additional file 4. Additional statistical explanation. [file 12931_2022_2126_MOESM4_ESM.pdf]

Supplementary document statistics

In a Bayesian analysis, the posterior distribution is determined containing information on both the prior belief about the treatment and the likelihood for the observed data, obtained from the trial. The posterior probability that zilucoplan has a superior improvement compared with SoC can be calculated. In calculating the approximate posterior probability for PaO_2/FiO_2 change, a vague reference prior (mean=0, sd= 10,000) is used. The corresponding vague reference prior for mortality (mean=0, sd=log(10,000)). From this the posterior probability that zilucoplan has a superior improvement compared with SoC can be calculated.
