# Supplementary figures and images for: Efficacy and safety of the investigational complement C5 inhibitor zilucoplan in patients hospitalized with COVID-19: an open-label randomized controlled trial
Source: Respir Res. 2022 Aug 9;23:202. doi: 10.1186/s12931-022-02126-2 (PMC9361275; doi:10.1186/s12931-022-02126-2)

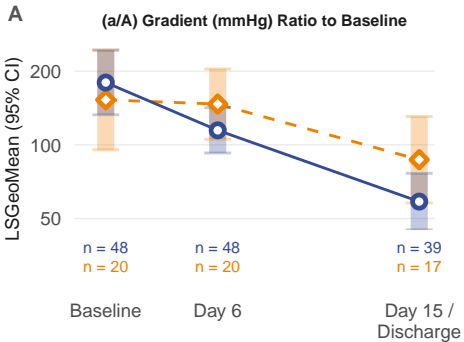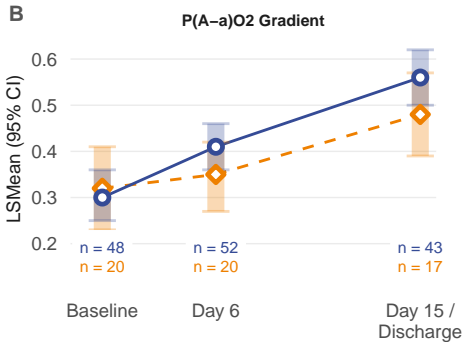

Supplement: Supplementary file 5 — Additional file 5: Figure S1. Primary Outcome. GeoMean, Geometric Mean; LSGeoMean, Least Square Geometric Means; LSMean, Least Square Means. [file 12931_2022_2126_MOESM5_ESM.pdf]

—○— Zilucoplan —◇— Control

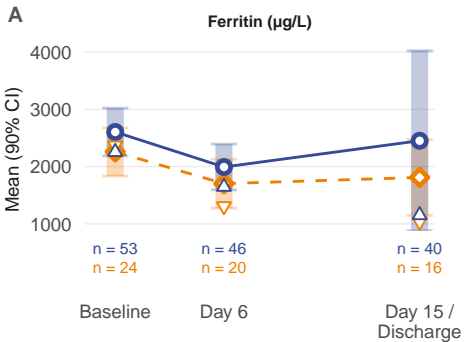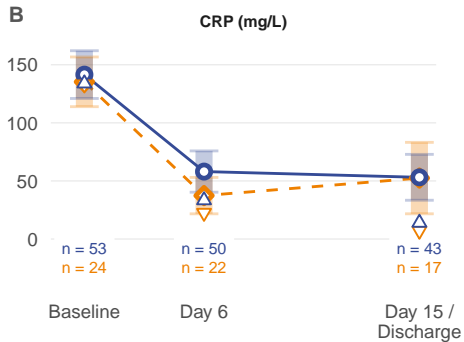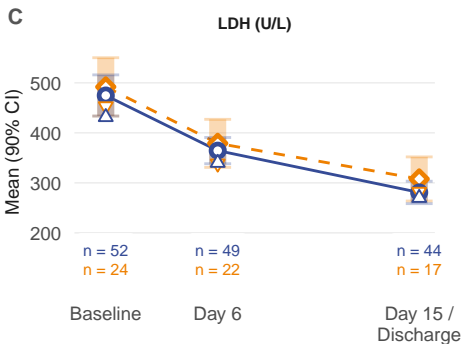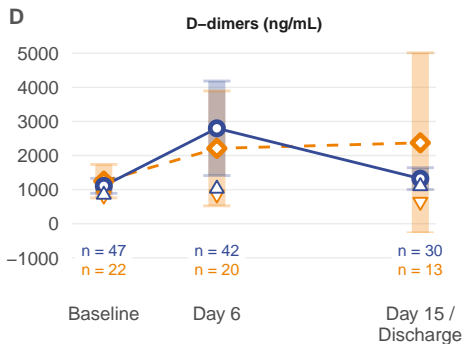

Supplement: Supplementary file 6 — Additional file 6: Figure S2. Laboratory Values. Medians are represented by triangles, with an upward point for Zilucoplan and a downward point for Control, respectively. CRP, C-reactive protein; LDH, lactate dehydrogenase. [file 12931_2022_2126_MOESM6_ESM.pdf]
