# Supplementary material for: Efficacy and safety of the investigational complement C5 inhibitor zilucoplan in patients hospitalized with COVID-19: an open-label randomized controlled trial
Source: Respir Res. 2022 Aug 9;23:202. doi: 10.1186/s12931-022-02126-2 (PMC9361275; doi:10.1186/s12931-022-02126-2)
Supplement: Supplementary file 7 — Additional file 7: Table S1. Primary and supportive endpoints in the full analysis data set. LSMean, least square mean; PaO2, arterial partial pressure of oxygen; FiO2, fraction of inspired oxygen; PaO2, partial pressure of arterial oxygen; ARDS, acute respiratory distress syndrome; CRP, C-reactive protein; CI, confidence interval; SD, standard deviation. *Based on the highest temperature in 24 h. Table S2. Follow-up endpoints. SD, standard deviation; DLCO, diffusing capacity of lung for carbon monoxide; HRCT, high-resolution computed tomography; WHO, world health organisation. 6-point ordinal scale: 2 on invasive mechanical ventilation; 3 on non-invasive ventilation or high flow oxygen devices; 4 hospitalized, requiring supplemental oxygen; 5 hospitalized, not requiring supplemental oxygen, 6 not hospitalized. [file 12931_2022_2126_MOESM7_ESM.docx]

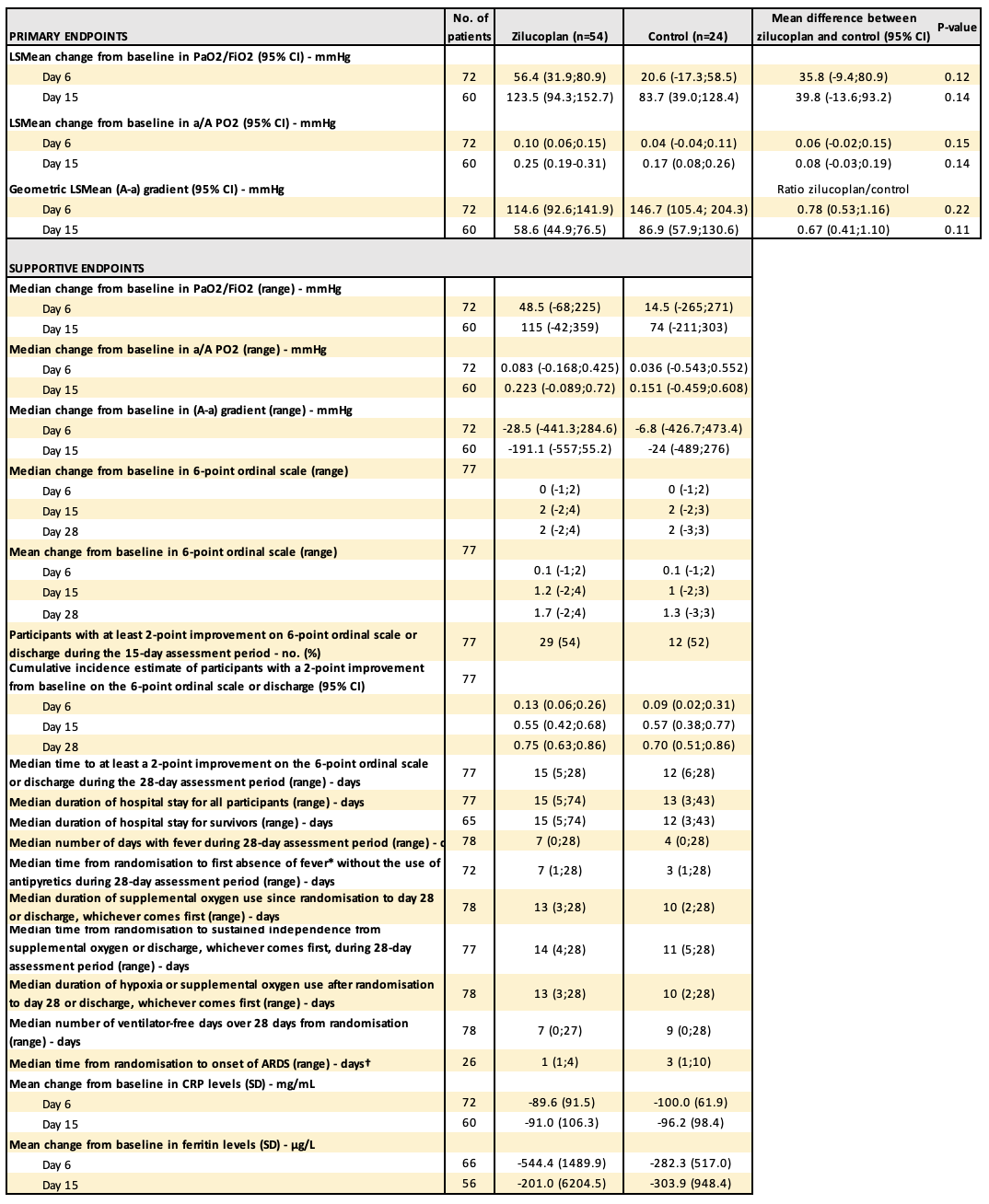


**Table S1**: **Primary and supportive endpoints in the full analysis data set.**

LSMean, least square mean; PaO_2_, arterial partial pressure of oxygen; FiO_2_, fraction of inspired oxygen; PaO_2_, partial pressure of arterial oxygen; ARDS, acute respiratory distress syndrome; CRP, C-reactive protein; CI, confidence interval; SD, standard deviation.

*Based on the highest temperature in 24 hours.

**
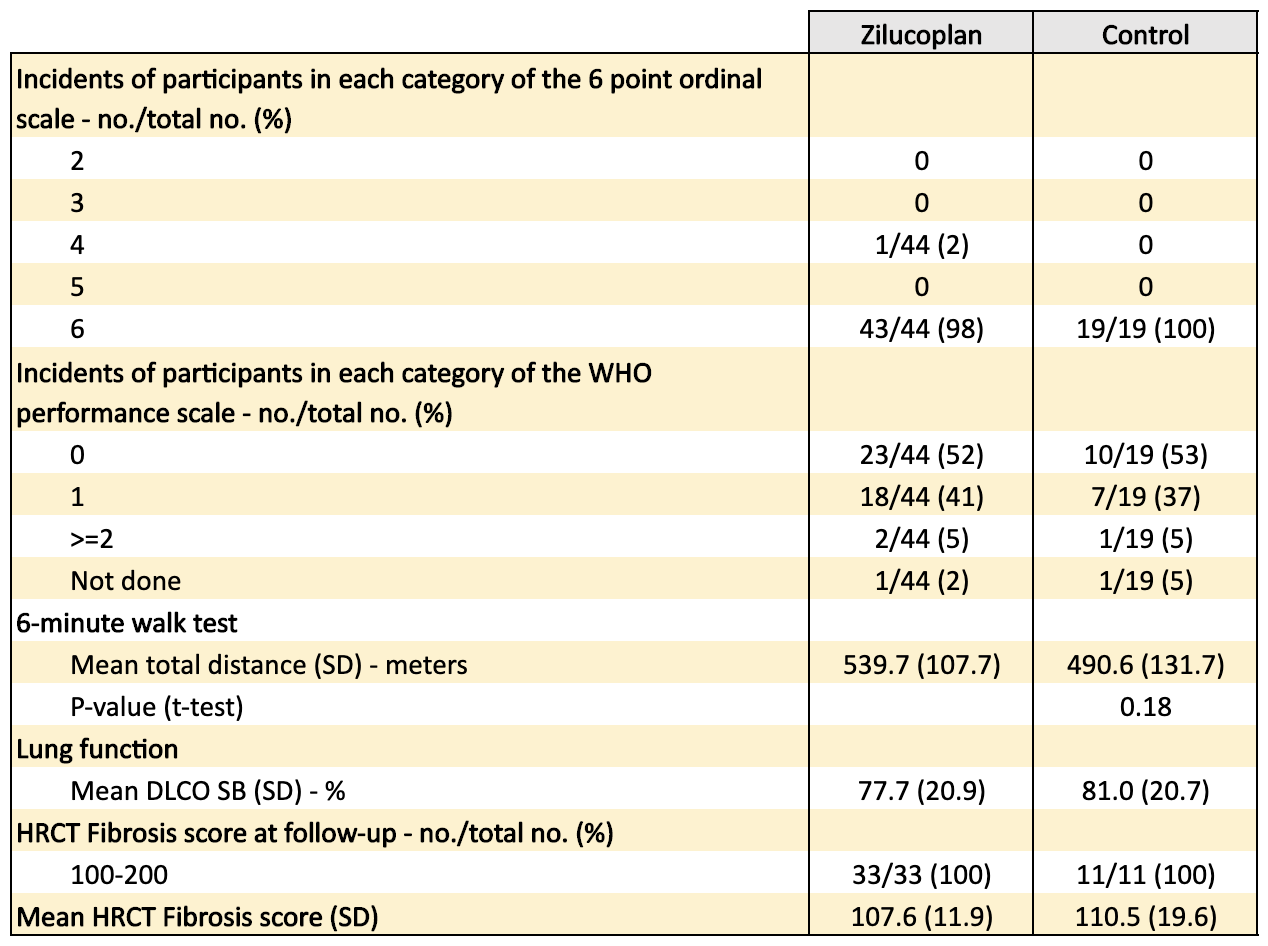
**

**Table S2**: **Follow-up endpoints.**

SD, standard deviation; DLCO, diffusing capacity of lung for carbon monoxide; HRCT, high-resolution computed tomography; WHO, world health organisation.

6-point ordinal scale: 2 on invasive mechanical ventilation; 3 on non-invasive ventilation or high flow oxygen devices; 4 hospitalized, requiring supplemental oxygen; 5 hospitalized, not requiring supplemental oxygen, 6 not hospitalized.
